# An Agent-Based Model of a Hepatic Inflammatory Response to *Salmonella*: A Computational Study under a Large Set of Experimental Data

**DOI:** 10.1371/journal.pone.0161131

**Published:** 2016-08-24

**Authors:** Zhenzhen Shi, Stephen K. Chapes, David Ben-Arieh, Chih-Hang Wu

**Affiliations:** 1 Health Care Operations Resource Center, Department of Industrial and Manufacturing Systems Engineering, Kansas State University, Manhattan, Kansas, United States of America; 2 Division of Biology, Kansas State University, Manhattan, Kansas, United States of America; Duke University School of Medicine, UNITED STATES

## Abstract

We present an agent-based model (ABM) to simulate a hepatic inflammatory response (HIR) in a mouse infected by *Salmonella* that sometimes progressed to problematic proportions, known as “sepsis”. Based on over 200 published studies, this ABM describes interactions among 21 cells or cytokines and incorporates 226 experimental data sets and/or data estimates from those reports to simulate a mouse HIR *in silico*. Our simulated results reproduced dynamic patterns of HIR reported in the literature. As shown *in vivo*, our model also demonstrated that sepsis was highly related to the initial *Salmonella* dose and the presence of components of the adaptive immune system. We determined that high mobility group box-1, C-reactive protein, and the interleukin-10: tumor necrosis factor-α ratio, and CD4+ T cell: CD8+ T cell ratio, all recognized as biomarkers during HIR, significantly correlated with outcomes of HIR. During therapy-directed *silico* simulations, our results demonstrated that anti-agent intervention impacted the survival rates of septic individuals in a time-dependent manner. By specifying the infected species, source of infection, and site of infection, this ABM enabled us to reproduce the kinetics of several essential indicators during a HIR, observe distinct dynamic patterns that are manifested during HIR, and allowed us to test proposed therapy-directed treatments. Although limitation still exists, this ABM is a step forward because it links underlying biological processes to computational simulation and was validated through a series of comparisons between the simulated results and experimental studies.

## 1. Introduction

Sepsis is initially activated by the presence and growth of pathogens in an organism. Under normal healthy circumstances, intruding pathogens are eliminated by the activation of immune cells, such as tissue macrophages and activated neutrophils, in the immune system [1, 2]. If an overwhelming immune response occurs, an unbalanced response between immune cells and cytokines may lead to unexpected harmful outcomes for patients, resulting in sepsis. In clinics, sepsis is defined as a potentially life-threatening complication of disease accompanied by symptoms such as high fevers, elevated heart rate, and altered mental status. If sepsis progresses to severe sepsis or septic shock, organ dysfunction occurs, leading to a high chance of death [3].

Severe sepsis and septic shock during an infection are the major causes of death in intensive care settings [4]. An average of 250,000 deaths per year in the United States (US) are caused by sepsis [5]. Among patients in intensive care units (ICUs), sepsis ranks as the second highest cause of mortality [6] and the 10th leading cause of death overall in the US [7]. An average of 750,000 sepsis cases occur annually, and this number continues to increase [6]. Care of patients with sepsis can cost as much as $60,000 per patient, resulting in a significant healthcare burden of nearly $17 billion annually in the US [8, 9]. Sepsis in a hospitalized patient can lead to extended hospital stays and subsequently increase financial burdens. Cross and Opal [10] discussed the lack of rapid, reliable assays available to identify the stage or severity of sepsis and to monitor the use of immunomodulatory therapy. Such assays are unavailable because of the complexity of the inflammatory response and the unpredictable nature of septic shock in individual patients; consequently increasing the difficulty of monitoring single or multiple components of inflammation with specific supportive therapies [10, 11].

A significant past focus on modeling immune responses during sepsis has emerged in an effort to explore the complicated dynamic presentation of cells, tissues, and cytokines during infection. In 2004, Kumar *et al*. [12] presented a simplified 3-equation system dynamics mathematical model (SDMM) to describe mathematical relationships between pathogen, early pro-inflammatory mediators, and late pro-inflammatory mediators in sepsis progression. In 2006, Reynolds *et al*. [13] proposed a mathematical model for acute inflammatory response (AIR) that included a time-dependent, anti-inflammatory response in order to provide insights into a variety of clinically relevant scenarios associated with inflammatory response to infection. Using a series of known and hypothesized kinetics of biological system components from the literature, mathematical models describe infectious disease processes by measuring steady states of various components in the immune system [14]. Unfortunately, these models fail to capture heterogeneous information of various components in the simulations and fail to account for deviations from various components’ aggregated behaviors [15].

The agent-based model (ABM), a powerful computational modeling technique, simulates complex nonlinear relationships between components and intuitively maps a realistic biological system by incorporating spatial effects and the stochastic nature of the immune response into model construction [16, 17]. One key element of ABM includes agents, a collection of decision-making entities classified into types based on components described in the real-world system. Each type of agent executes behaviors that can mimic the system they represent when aggregated. Implementation of a predefined set of rules allows agents to move in a designed direction and arbitrarily interact with other agents in a spatial environment. Agent behaviors are updated in various locations according to update rules executed at discrete time steps. ABM inherently captures repetitive spatial interactions between agents in a stochastic process or under a known probability distribution, making it a powerful tool to render valuable information and simulate a biological system. Implementation of ABM requires well-established technology that relies on computers to explore dynamics beyond the reach of pure mathematical methods [18, 19]. The inherent nature of computational structure allows ABM to be efficiently implemented on parallel computers [20].

An and his collaborators [21–23] developed a series of agent-based models to simulate behaviors of cells and cytokines in both the innate and adaptive immune system of a generalized inflammatory response. In 2011, Wu *et al*. [24] proposed an integrated ABM embedded with a mathematical model to simulate AIR progression at the interface between blood vessels and cells within a hypothetical generalized organ. Recently, Dutta-Moscato *et al*. [25] proposed a multi-scale agent-based *silico* model of liver fibrosis using an ABM to simulate an HIR. In addition to modeling interactions between cells, Dong *et al*. [26] proposed an ABM framework to model intracellular dynamics of the NF-*k*B signaling cascade, illustrating subsequent intercellular interactions among macrophages and T-helper cells through the up-regulation of inflammatory mediators. Their approach explored hypothetical scenarios of AIR and potentially improved the understanding of molecular behaviors that could develop and expand to emergent behavior of the entire AIR system. In addition to these related work, there are studies that presented the application of agent-based models to simulate various types of disease progression [27–30].

Existing ABMs provide evidence that agent-based modeling is a valid approach for simulating disease progression [21, 22, 24–26]. In this study, we proposed an integrated-mathematical-multi-agent-based model (IMMABM) to simulate mouse HIR caused by *Salmonella* at the tissue level. By specifying the infected species, source of infection, and site of infection, the scope of the IMMABM allowed us to improve modeling approach accuracy without loss of generality. This IMMABM required that each interaction incorporated into the model was based on actual data from observations made during experimental infections *in vivo* or measurements made *ex vivo* or *in vitro*, thereby resulting in an incorporation of 226 experimental data from 210 publications related to mouse hepatic inflammation induced by *Salmonella*. When data were not available, we extrapolated from related Gram-negative bacteria or other pathogens, keeping in mind that fidelity to actual *Salmonella* infections was necessary. Therefore, we summarized interactions among cells, tissues, and cytokines during mouse HIR and we calibrated quantitative changes in the HIR with experimental data and necessary mathematical expressions for agent modeling. We attempted to calibrate variables based on unit relationships observed in the experimental systems. A key objective of our IMMABM was to incorporate available experimental data into the computational simulation.

Simulated results from IMMABM showed that four distinct dynamic patterns emerge during mouse HIR: a healing response, persistent infection, a hyperinflammatory response, and organ dysfunction. Emerging simulations were verified through a pattern-oriented analysis found in available mouse experimental studies. Furthermore, simulated results from IMMABM determined that expression of high mobility group protein 1 (HMGB-1), C-reactive protein (CRP), interleukin 10 (IL-10): Tumor necrosis factor alpha (TNF-α) ratio, and the CD4+ T cell: CD8+ T cell ratio are highly correlated with the outcomes of mouse HIR. We also correlated mouse HIR to the initial *Salmonella* challenge level during IMMABM simulation. Most importantly, we observed that the survival rates during HIR are dependent on the time of administering antimicrobial or anti-cytokines (anti-TNF-α and/or anti-HMGB-1) treatments.

## 2. Materials and Methods

### 2.1 Simulation environment of the IMMABM for HIR

This IMMABM attempted to simulate a *Salmonella* infection in the mouse liver. The liver, enriched with resident tissue macrophages (Kupffer Cells), is recognized as a key organ of the immune system that is vital for elimination of a *Salmonella* infection [31, 32]. We chose *Salmonella* as a “targeted” pathogen strain because it is responsible for millions of deaths in developing countries every year [33]. Furthermore, immune responses to *Salmonella* infections have been investigated extensively [34–39]. Therefore, an abundance of data is available for accurate incorporation of relationships among variables (agents) in order to support our IMMABM.

### 2.2 Software platform

We used NetLogo 5.1.0 [40], a platform with a simplified programming environment, easily implemented tool sets, and well-established documentation support, in order to implement the IMMABM [41]. The primary user interface of NetLogo is comprised of two-dimensional (2D) grids, and agents can be divided into two categories: “patches” and “turtles.” "Patches" are fixed agents placed on background grids in the model workspace. “Turtles” are mobile agents that occupy a position or move freely on the surface of patches and execute certain functions or actions regulated by the simulated system. NetLogo also applies a class called “breed” to define agent types with similar behaviors or types that are controlled by the same mechanism. The concept of “breed” allows the modeler to define a class of agents with a set of common state variables and establish various functions or actions (autonomous behaviors) for agent types. The interface of NetLogo allows the modeler to set initial parameters and observe simulation results.

### 2.3 Simulation initial setting

**Fig 1:**
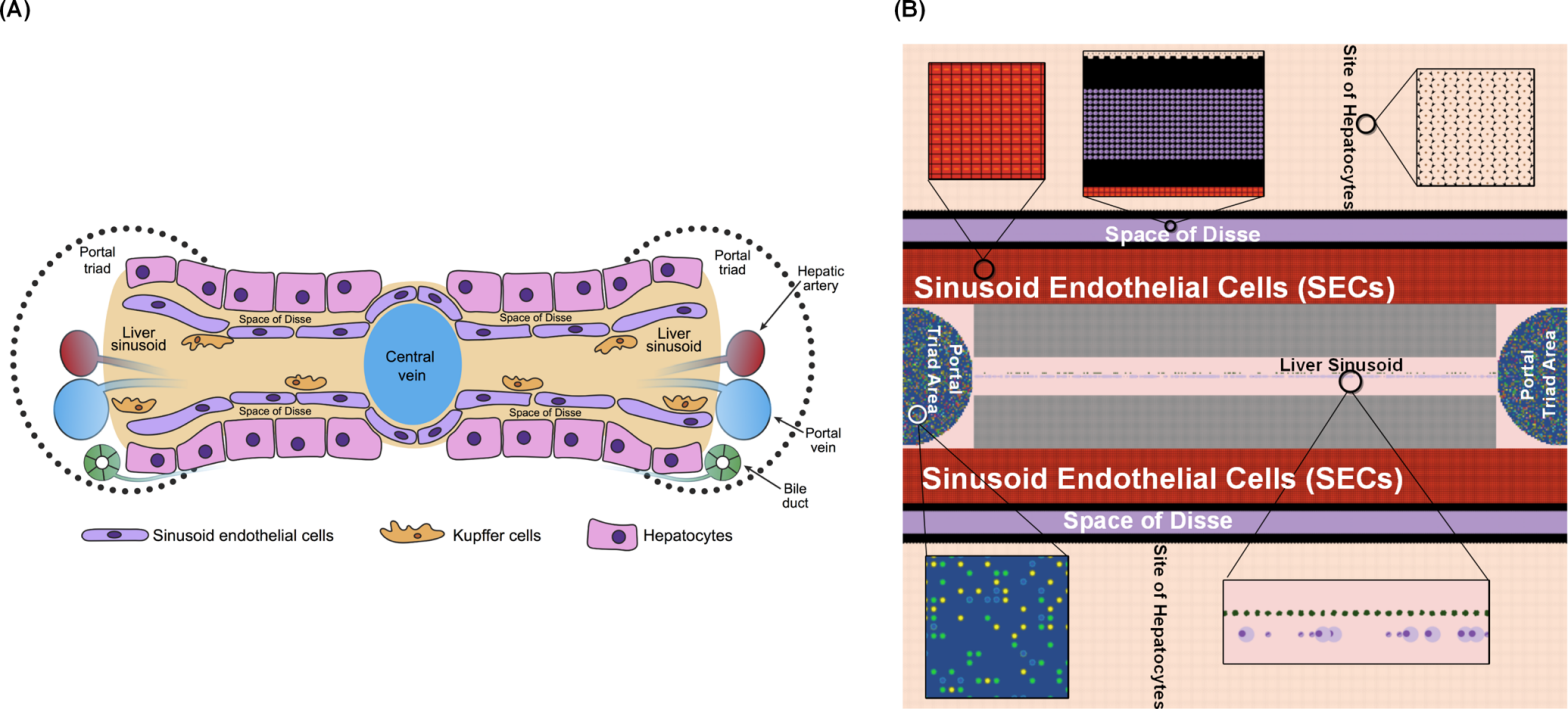
A comparision between liver structure in mouse and simulated liver structure in NetLogo at simulation step equal to 0. (A) Diagrams of 2D liver structure in mouse. (B) Simulated area of the HIR in the NetLogo interface at simulation step equal to 0.

We generated a 401 × 401 2D grid in NetLogo as the simulation interface, designed to simulate a 2D projection of a mouse liver. We focused on the cellular interactions between liver sinusoid and hepatocytes in mouse. The interface of cellular interactions is comprised of five main compartments: liver sinusoid, sinusoid endothelial cells (SECs), the space of Disse, the site of hepatocytes, and portal triad (Fig 1A) [42]. The Portal triad is a complex area including the hepatic artery, portal vein, and bile duct [42]. Blood flows from the portal triad area to the liver sinusoid, which carries blood-borne pathogens (i.e. *Salmonella*) to the site of hepatocytes. Hepatocytes are separated from the liver sinusoid by the space of Disse and sinusoid endothelial cells [42]. Kupffer Cells are distributed along sinusoid endothelial cells, and are able to ingest and kill the blood-borne *Salmonella* [43]. To mimic this liver structure, we divided the entire interface of NetLogo into five regions to represent the liver sinusoid, SECs, the space of Disse, the site of hepatocytes, and portal triad in the liver [43]. In the *silico* simulated environment, the probabilities that different agents (cells, cytokines) interact are more important than the actual physical morphology, which in *vivo* determines how these agents will interact. The choice of agents is directly comparable to the cell types and tissue organization formed in the liver. Therefore, the NetLogo setup is appropriate for this model. The initialized interface of NetLogo is shown in Fig 1B. Kupffer Cell numbers are approximately one-fourth the number of hepatocytes in the liver [42]. SEC numbers are approximately one-third the number of hepatocytes, and approximately one-eighth the number of mast cells exist compared to the number of hepatocytes [42, 44]. The initial number of hepatocytes was determined by an automated process of filling the region with hepatocytes in a 401 × 401 2D grid. For simulation size presented in this paper, the number of hepatocytes was initialized to 80,200. Considering the numeric proportion between hepatocytes, Kupffer Cells, SECs, and mast cells, we set the initial number of Kupffer Cells to 20,160, SECs to 26,466, and mast cells to 10,426. Detailed information on the initialization process of the IMMABM is provided in S1 Table.

### 2.4 Process of IMMABM development

IMMABM was developed as an agent-oriented computer program to describe agent rules and behaviors. Each agent type was defined as “breed” in NetLogo, and each “breed” in IMMABM had specific state variables. By assigning various values to the state variables, the agents were regulated to execute a series of functions based on various locations and environmental interfaces. Interactions between agents were highly stochastic, and we incorporated mathematical expressions such as logistic growth functions, mass-action kinetics, Michaelis-Menten kinetics, and decay functions to quantitatively measure complicated biological processes. Furthermore, the process of writing computer codes strictly followed conditional statement “if-then” rules. Those rules conformed to biological mechanisms of HIR.

The primary objective of our IMMABM was to incorporate available experimental data into the computational simulation. Data such as infiltration time of immune cells, replication rate of *Salmonella*, and degradation rate of associated mediators allowed us to advance the ABM application by mapping biological processes that occur during HIR to our IMMABM. By integrating experimental data and mathematical expressions derived from hypothesized kinetics, we attempted to quantitatively simulate dynamic patterns of HIR. In addition, a global variable defined as “Infection Time” in IMMABM reflected simulation execution time and mimicked kinetic associations between a series of responses. In our simulation, 1 tick (representing 1 simulation step in the simulation software) represented 1 hr in an actual biological process, and numeric counts of an agent were updated per tick to correspond to the biological response time in the experiments.

Incorporation of data from 210 publications and our experience with *Salmonella* infections and infectious disease processes motivated us to select a total of 23 essential cells and cytokines as agent types in this IMMABM. In this paper, we use italic format to highlight agent type for convenience. Each agent type, further defined as “breed”, could contain multiple entities. Among the 23 types of agent, we defined 20 primary agent types: *Hepatocyte*, *Hepatocyte Debris*, *Kupffer Cell*, *Salmonella*, *Mast Cell*, *Resting Neutrophil*, *Activated Neutrophil*, *Resting Monocyte*, *MDMI (monocyte-derived type 1 macrophage)*, *MDMII (monocyte-derived type 2 macrophage)*, *TNF-α (tumor necrosis factor-α)*, *HMGB-1 (high mobility group box-1)*, *IL-10 (interleukin-10)*, *CD4 T Cell*, *CD8 T Cell*, *B Cell*, *Antibody*, *CRP (C-reactive protein)*, *NET (neutrophil extracellular traps)*, and *Histamine*. We also defined three auxiliary agent types: *SEC (sinusoidal endothelial cell)*, *Signal*, or *Anti-Signal*. The rule system for these agents was based on the literature. A sequence of interactions among primary agents and primary agent behaviors during interactions are introduced in Section 2.4.1.

#### 2.4.1 Primary agent behaviors

*Salmonella*, a “trigger” to begin HIR, was the first agent to move and interact with *Kupffer Cells*, thereby initializing HIR. The percentage of *Salmonella* killed by *Kupffer Cells* was set from 15% to 16% of the total *Salmonella* population per hour because 90% to 95% of *Salmonella* were phagocytosed (engulfed) by *Kupffer Cells* in 6 hrs [45]. *Salmonella* that survived in *Kupffer Cells* turned *Kupffer Cells* into an “apoptotic” state and “proliferated” within *Kupffer Cells* [46, 47]. “Die” in Netlogo occurs when an agent in the simulation is forced to disappear, but “proliferate” is defined as new agent generation in the simulation. State variables associated with agent type were used to define various states of individual agents. Implementation of state variables is introduced in section 2.4.3. The maximum number of *Salmonella* that “proliferate” within one *Kupffer Cell* was limited to 50 [31]. The newly generated *Salmonella* were released to liver tissue after “apoptotic” *Kupffer Cells* “died” between 6 and 14 hrs [46]. These released *Salmonella* were assigned to a state variable “*Salmonella*NewlyReleasedFromKupfferCell” in order to express aborted interaction with *Kupffer Cells* and prepare for “proliferation” in surrounding *Hepatocytes* or *SECs* [31, 48]. When *Activated Neutrophils* or *MDMIs* were recruited to the site of infection, *Neutrophils* or *MDMIs* “killed” *Salmonella* [45, 46, 49, 50]. Experimental data showed that every neutrophil phagocytized approximately 3 to 13 *Salmonella* per hour, and every *MDMI* phagocytized approximately 1 to 7 *Salmonella* per hour [51]. In addition to immune cells, *CRP* released from *Hepatocytes* and *Antibody* released from *B Cells* also contributed to the “killing” of *Salmonella* [52–56].

*Hepatocytes* account for approximately 60% of the total number of cells in the liver [42]. In our IMMABM, *Hepatocytes* were primary locations for *Salmonella* “proliferation”, and the *Hepatocytes* become “apoptotic” once they interacted with *Salmonella* or *TNF-α* [32, 45, 48]. “Apoptotic” *Hepatocytes* released acute stress proteins such as *CRP*, or cytokines such as *TNF-α*, and *HMGB-1* [52, 53, 57, 58]. In addition, “Apoptotic” *Hepatocytes* could undergo a natural aging process or interact with infiltrating *Activated Neutrophils* [59–61]. “Apoptotic” *Hepatocytes* that interacted with *Activated Neutrophils* “died” immediately and released their interacted *Salmonella* to the liver tissue [45]. Alternatively, “Apoptotic” *Hepatocytes* underwent natural aging and became *Hepatocyte Debris* after 2 or 3 hrs [62]. In addition to death, *Hepatocyte* was also regenerated at a rate of 1.32×10^−3^ to 6.80×10^−3^ per hour to simulate proliferation of *Hepatocyte* in a mouse’s liver [63].

We modeled five primary phagocytic cells in our IMMABM, including *Kupffer Cell*, *Mast Cell*, *Activated Neutrophil*, *MDMI*, and *MDMII*. *Kupffer Cells* reside principally within the lumen of liver sinusoids, adherent to *SECs* that comprise blood vessel walls [43]. The first phagocytic cells that interacted with *Salmonella* in the liver [64–66] were *Kupffer Cells* that killed approximately 90% to 95% of the *Salmonella* population in 6 hrs; however, 5% to 10% of *Kupffer Cells* were killed by *Salmonella* during the same time period [45]. *Kupffer Cells* released cytokines such as *TNF-α* which helped recruit other phagocytic cells such as *Activated Neutrophils* to the site of infection or incurs further damage to *Hepatocytes* [32, 67]. *Kupffer Cells* also released *IL-10*. *IL-10* represents anti-inflammatory cytokines in this model and is capable of inhibiting the release of TNF-α. As typical phagocytic cells, *Kupffer Cells* “killed” various types of cell debris such as hepatocyte debris (to represent their scavenging or “clean up” function), *Antibody*-opsonized *Salmonella*, and *CRP*-opsonized cell debris [54, 55]. The apoptosis of *Kupffer Cells* occurs at a rate of 4.20 ×10^−3^ to 3.20×10^−2^ per hour [68]. Replenishment of *Kupffer Cells* came from *MDMIs* and *MDMIIs* at a rate of 6.30×10^−3^ to 7.90×10^−3^ per hour [68]. Similar to *Kupffer Cells*, *Activated neutrophils* also “killed” *Salmonella*, *Antibody*-opsonized *Salmonella*, *CRP*-opsonized cell debris, and released cytokines such as *TNF-α* or *IL-10* [54, 55, 69, 70]. Biologically, *Activated Neutrophils* were recently recognized to release *NETs* to eliminate *Salmonella* [71, 72]. *Activated Neutrophils* underwent natural aging or were “killed” by *Kupffer Cells* [43]. “Apoptotic” *Activated neutrophils* underwent apoptosis, indicated by a state variable labeled “apoptotic.” The “apoptotic” *Activated Neutrophils* were killed by *MDMIs* [73]. *MDMIs* were activated from *Monocytes* between 6 hrs to 24 hrs post-infection [74, 75]. The activation level of *Monocytes* was dependent on the existing number of *Salmonella*, *TNF-α*, *HMGB-1*, “apoptotic” *Activated neutrophil*, *CD4+ T cell*, and *CD8+ T cell*. The activation amount was calculated based on Michaelis-Menten kinetics, as discussed in Section 2.4.4. Upon activation, *Monocyte* became *MDMI* or *MDMII* [73]. *MDMI* “killed” *Salmonella* and released *TNF-α* [73], while *MDMII* “killed” “apoptotic” *Activated Neutrophils* and released *HMGB-1* and *IL-10* as mediators to resolve the inflammation [73, 76]. *MDMI* and *MDMII* helped activate T cell recruitment [77], and both *MDMI* and *MDMII* released *IL-10* when they “killed” apoptotic *CD4+ T Cell* or *CD8+ T Cell* [78].

*CD4+ T Cell*, *CD8+ T Cell*, and *B Cell* occupied spaces in the portal triad [55, 79]. Activation of *CD4+ T Cell*, *CD8+ T Cell*, or *B Cell* occurred when *MDMI* or *MDMII* were detected [77], at which point the activated *CD4+ T Cell*, *CD8+ T Cell*, or *B Cell* moved from the portal triad to the liver sinusoid [80]. *CD4+ T Cell* released *TNF-α* or *IL-10* when they interacted with phagocytic cells interacting with *Salmonella*, and *CD 4+ T Cell* improved the phagocytic rate of phagocytic cells [56]. *CD8+ T Cell* “killed” phagocytic cells that interacted with *Salmonella* [55, 56]. *CD4+ T Cell* and *CD8+ T Cell* underwent natural apoptosis, and both “apoptotic” *CD4+ T Cell* and *CD8+ T Cell* were “killed” by *MDMI* or *MDMII* [78]. *B Cell* released *Antibody* to form an *Antibody*-*Salmonella* complex, and the *Antibody*-*Salmonella* complex was killed by phagocytic cells, simulating opsonization [56]. The binding process is described in Section 2.4.2.

*TNF-α*, *HMGB-1*, and *IL-10* are cytokines released from phagocytic cells. *TNF-α* was released from *Kupffer Cell*, *Mast Cell*, “apoptotic” *Activated Neutrophil*, *MDMI*, and *Hepatocyte* [32, 43, 70, 73, 81–84]. *TNF-α* caused *Hepatocyte* to become “apoptotic” [32]. *HMGB-1* was released from *MDMII* and “apoptotic” *Hepatocyte* [85–87], and *IL-10* was released from *Activated Neutrophil*, *MDMII*, and *CD4 T Cell* [69, 73, 81, 87–89]. *IL-10* prevented secretion of *TNF-α*, *HMGB-1*, and *IL-10* from interacting with phagocytic cells or T cells [88, 90–94]. Procedurally, *TNF-α*, *HMGB-1*, and *IL-10* “died” to reflect their clearance away from the simulation. An overview of agent behaviors is provided in S1 Table.

#### 2.4.2 Agent and agent complex movement

Agent movement in IMMABM was determined by agent behaviors described in S1 Table. For example, *Resting Neutrophils* or *Resting Monocytes* moving to *SECs* were driven by *Signals* released from cytokines or cells [56, 59, 67, 73, 85, 95–97]. When *Signals* appeared on *SECs*, *Resting Neutrophils* or *Resting Monocytes* moved to *SEC* locations. Mass-action kinetics determined the number of moving *Resting Neutrophil*s or *Resting Monocytes*, as described in Section 2.4.4. Biological interaction between two agents occurred in IMMABM simulation if two agents occupied the same patch.

*Salmonella* that replicated within *Kupffer Cells*, *MDMIs*, *SECs*, and *Hepatocytes* [31, 46–48, 73] were released to nearby patches when infected cells “died”. Released *Salmonella* randomly moved to the nearest *Hepatocytes* and damaged those *Hepatocytes*.

We used a “link” breed to model movements of the *Antibody*-*Salmonella* complex or *CRP*-cell complexes because the two components of the complex need to move simultaneously. For example, when an *Antibody*-*Salmonella* complex moved to one phagocytic cell, *Antibody* and *Salmonella* moved in the same direction for the same distance [55]. The *Antibody*-*Salmonella* complex’s killing process using the phagocytic cell occurred when the distance between the complex and the phagocytic cell was less than one patch-size.

#### 2.4.3 State variable updates

Each agent type had its own state variables in the IMMABM. By assigning various values to the state variables, the agents were regulated to execute a series of functions based on locations and environment interfaces. During a simulation, some state variable are fixed through the simulation, and others change as the simulation runs. The change in values of state variables is based on the change of agent behaviors during the simulation. A detailed description of agent behaviors in *vivo* is provided in S2 Table. For example, the value of some state variables was set to either 0 or 1, and the function of these state variables was similar to a switch: 0 represented “off”, and 1 represented “on”. If a state variable was equal to 1, individual agents that had that state variable would express specific attributes or execute biological functions. For example, *Kupffer Cell* had a state variable named “kupfferCellBindToIL10”. The value of the state variable was equal to 1 when *Kupffer Cell* interacted with *IL-10*, and individual *Kupffer Cells* that had the state variable “kupfferCellBindToIL10” equal to 1 did not release *TNF-α*. *Salmonella* that proliferated within *Kupffer Cell* had a state variable named “*Salmonella*ReplicateWithinKupfferCell” equal to 1; *Salmonella* that had “*Salmonella*ReplicateWithinKupfferCell” equal to 1 generated new agents until the state variable “*Salmonella*ReplicateWithinKupfferCell” was reset to 0. *Kupffer Cell* was assigned to a state variable named “kupfferCellKillBy *Salmonella*” equal to 1 when *Kupffer Cells* interacted with *Salmonella* that had the state variable “*Salmonella*ReplicateWithinKupfferCell” equal to 1. *Kupffer Cells* had the state variable “kupfferCellKillBy *Salmonella*” equal to 1 “die” after 6 simulation ticks, and the state variable “*Salmonella*ReplicateWithinKupfferCell” of interacted *Salmonella* was reset to 0. *Resting neutrophil* were activated in order to move to *SECs* in response to signaling by *TNF-α*, *HMGB-1*, or *Salmonella* signaling, consequently becoming *Activated neutrophil*. *Activated neutrophil* moved to the “apoptotic” *Hepatocytes* with a state variable labeled “hepatocyteUndergoApoptosis” equal to 1. “Apoptotic” *Hepatocytes* that interacted with *Activated neutrophils* “died” immediately due to the killing process of “apoptotic” *Hepatocyte* by *Activated neutrophil* [49, 74]. A comprehensive description of agent rule updates is presented in S1 Table.

#### 2.4.4 Mathematical equations in programming

In order to calibrate quantitative changes in agent number during HIR, we used a standard logistic function to measure cell population increases, Michaelis-Menten kinetics to calibrate cytokine release, mass-action kinetics to calibrate the activation process of circulating neutrophils and monocytes, and a decay function to measure the natural process of apoptosis by cells or catabolism of cytokines.

For example, we calibrated the *Salmonella* population to increase using a standard logistic growth function [98] as follows:

$$\frac{dP}{dt}=k_{pg}P\left(1-\frac{P}{P_{\infty }}\right)$$ (1)

In Eq (1), *P* represents the *Salmonella* count, *K*_*pg*_ represents a constant growth rate for *Salmonella*, and *P*_*∞*_ represents maximum carrying capacity of the *Salmonella*. Growth rates and carrying capacities of *Salmonella* varied when *Salmonella* replicated within various cells. Corresponding experimental data is presented in S3 Table.

The activation process of circulating neutrophils was promoted by the pro-inflammatory mediator TNF-α, *Salmonella*, and HMGB-1 [56, 59, 67, 85, 95]. We used a mass-action kinetics equation [99] to calibrate the activation process of circulating neutrophils as follows:

$$\frac{dN}{dt}=rN_{R}(T^{*}+P^{*}+H^{*})$$ (2)

In Eq (2), *N* represents *Activated Neutrophil* count and *N*_*R*_ represents *Resting Neutrophil* count. T* denotes concentration of TNF-α, P* denotes concentration of *Salmonella*, and H* denotes concentration of HMGB-1.

The release of cytokines obeyed trafficking machinery, and cytokines were released via protein-protein interactions initiated by ligand binding to receptors [100, 101]. Therefore, we used Michaelis-Menten kinetics [102] to calibrate the cytokine release process as follows:

$$\frac{dC}{dt}=\frac{K_{max}Cell}{{Cell}_{half}+Cell}$$ (3)

In Eq (3), *C* represents cytokine count and *K*_*max*_ represents the maximum production rate of cytokines secretion by the cell. *Cell* denotes current numbers of the cell intending to release the cytokine and *Cell*_*half*_ denotes cell numbers when half the maximum production rate of the cytokine was reached in the IMMABM.

Natural cell apoptosis or cytokine catabolism occurred at every tick; thus, we assumed that the decrease in cell or cytokine counts followed a simple decay function as follows:

$$\frac{dC}{dt}=K_{c}C$$ (4)

In Eq (4), C represents cell or cytokine count and Kc represents a constant decay rate for cells or cytokines.

#### 2.4.5 Model calibration and parameter estimation

In addition to mathematical models, we calibrated experimental data such as replication rates of cells, production rate of cytokines, killing rates of *Salmonella* by phagocytic cells, activation rates of circulating neutrophils or monocytes, and apoptotic rate of cells or catabolism of cytokines from existing experimental studies. These data were incorporated into IMMABM as system parameters. We collected experimental data from studies that were most similar to our simulation setting. We also estimated parameters during simulation if data were not available from experimental studies. For example, we estimated that the CRP-opsonized debris moved to inflammatory cells (e.g. Kupffer Cell, mast cell, neutrophils, MDMI, and MDMII) with an equal chance of 0.2. An overview of estimated experimental data is provided in S3 Table.

In order to smoothly translate estimated experimental data to agent-based modeling, we made corresponding assumptions in terms of data estimation. In general, we assume the change in rate is constant because we observed changes in data of interests in most of experimental studies following linear curves. Some experimental data is comprised of multiple linear segments, and therefore we calibrated rates for each linear segment to measure various rates for multiple responding time periods. These multiple rates are explained in S3 Table. The release/secretion rates of various cytokines (TNF-α, HMGB-1 and IL-10) by inflammatory cells such as neutrophils, Kupffer Cells and monocyte-derived-macrophages are described as a function of time, by possibly incorporating the effect of decay/catabolism. Experimental data are integrated into our agent-based model as inputs by ignoring different experimental conditions/settings such as different initial loads of bacteria injection, different bacteria strains, different animal models, etc. This limitation could be reduced by additional experiments done under the same experimental conditions/settings. It was not possible to extrapolate the data for our agent-based model from one simple experimental model. The strategy we used was to focus on mouse *Salmonella* infection studies that were published in papers available in the NCBI. When necessary, we used data from broader systems such as Gram-negative infections (i.e. *E*. *coli*) or even Gram-positive bacterial infections. Therefore, we are aware that some of these assumptions may not be correct.

In IMMABM, we used agent count to represent cell number with the awareness that cytokine production rate has a unique experimental unit compared to cell number. Thereby, cytokine production rate had to be transformed into an agent number in order to make the experimental data consistent in IMMABM. Therefore, we used one agent count to represent one real experimental unit. For example, we estimated that one phagocytic cell can bind 1.23×10^−17^ g IL-10 from experimental data [103]. Therefore, we used one IL-10 agent count to represent 1.23×10^−17^ g IL-10 in real experiments. Similarly, 1.25×10^−11^ g CRP could bind to one phagocytic cell based on our calibration [104]. Thus, we used one CRP agent count to represent 1.25×10^−11^ g CRP in real experiments. Data showed that 2.82×10^−17^ g TNF-α damaged one hepatocyte per hour [105], so we used one TNF-α agent count to represent 2.82×10^−17^ g TNF-α in order to transform experimental units into the agent count. Unfortunately, however, *NET* structure is fragile, thereby making it difficult to quantify *NETs* in experiments [106]. The rate at which NETs kill *Salmonella* was also difficult to establish [107]. Therefore, since neutrophil elastase (NE) is required for *NET* formation and NE is an essential component of *NET* [71], we used the rate at which NE kill *Salmonella* to substitute for the rate at which *NETs* kill *Salmonella*.

## 3. Results

### 3.1 Statistical analysis

Results are expressed as mean ± standard error (SE). Data normality was checked using both histogram and quantile-quantile (Q-Q) plot. For normally distributed data, group comparisons were performed using one-way analysis of variance (ANOVA). For non-normally distributed data, Mann-Whitney *U* tests were conducted to compare groups. All tests were performed using R 3.1.2. [108]. A *P* < 0.05 was considered statistically significant at the significance level α = 0.05.

### 3.2 Change in selected indicator levels observed under various *Salmonella* loads

**Fig 2:**
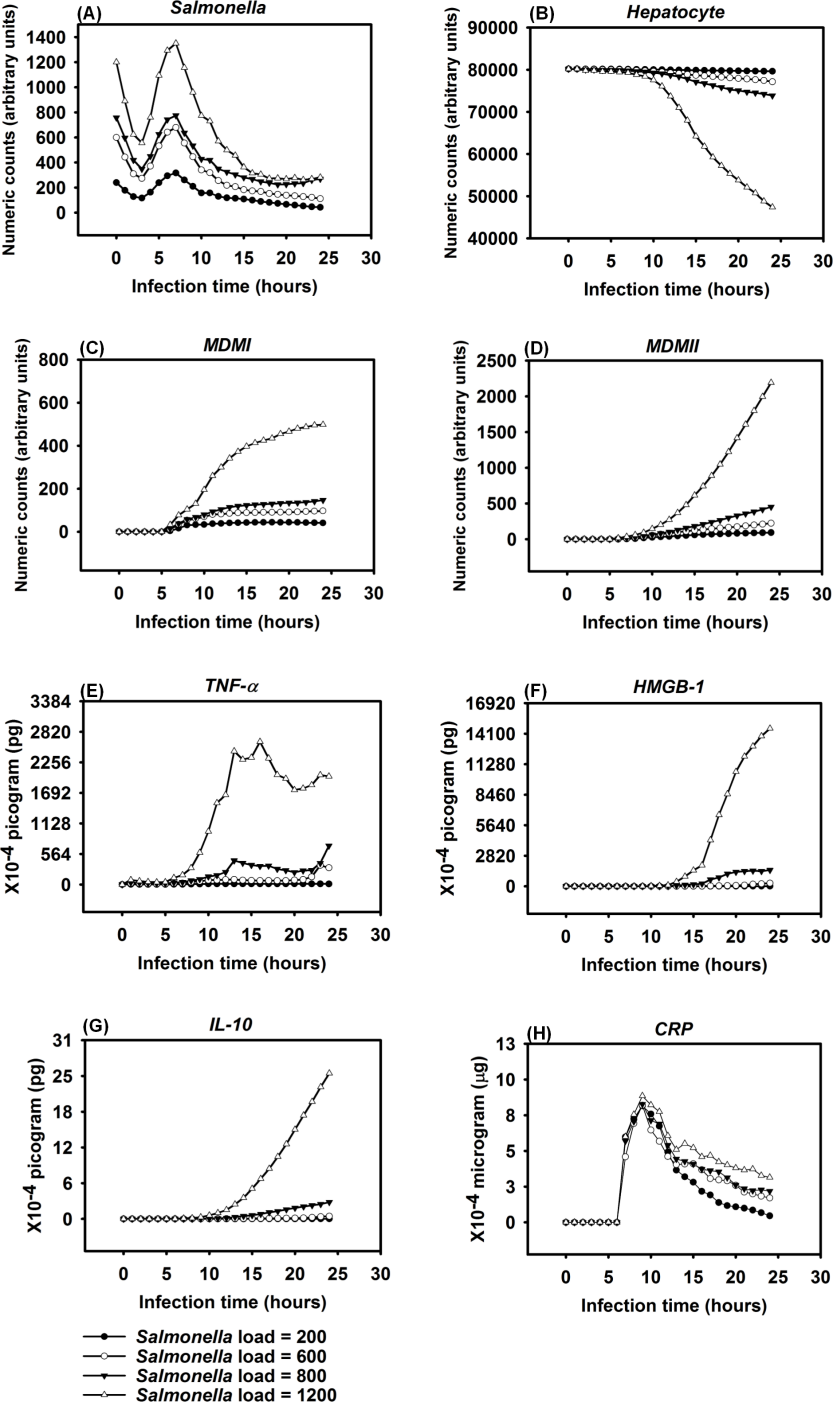
Response of different variables (agents) over the first 24 hrs after *Salmonella* infection (load) of 200 counts, 600 counts, 800 counts, and 1200 counts. Mean counts of indicators were measured at each simulation time point (replications = 100).

The IMMABM generated quantitative results by running simulations with various initial *Salmonella* counts (equivalent to infection dose). The input data, converted as described to mathematical expressions and incorporated into the computer code, assembled cellular and molecular variables in order to generate a hypothetical immune response. Clinical and experimental data showed that the risk of patients dying from sepsis is significantly correlated to the initial dose of pathogen [109, 110]. Therefore, we hypothesized that HIR would have a higher likelihood of progressing to septic shock and death if the infection was initially high. To test this hypothesis, the IMMABM in *silico* simulations were carried out using *Salmonella* doses of 200 counts, 600 counts, 800 counts, and 1200 counts, and 100 replicated runs for each proposed *Salmonella* dose to explore the possible stochastic nature of the model. Results from these simulations were initially generated to identify dynamic patterns of indicators in HIR with various initial *Salmonella* doses, as shown in Fig 2.

One significant finding from the simulations was that outcomes of HIR were highly correlated to initial *Salmonella* “counts” (the *in silico* equivalent to colony forming units, CFU, although there is not a 1:1 correlation between counts and CFU). We observed that *Salmonella* counts, phagocytic cell (*MDMI* and *MDMII*) counts, and pro- and anti-inflammatory cytokine (*TNF-α*, *HMGB-1* and *IL-10*) counts increased as *Salmonella* infection (load) increased. Specifically, the number of phagocytic cells and the concentration of inflammatory cytokines significantly increased (based on one-way ANOVA tests with significance level α = 0.05 and *P* < 0.05) when *Salmonella* infection (load) increased from 800 counts to 1200 counts. A significant decrease (based on one-way ANOVA tests with significance level α = 0.05 and *P* < 0.05) in hepatocyte counts was also observed when *Salmonella* infection (load) increased from 800 counts to 1200 counts. The dose-response hypothesis test initially indicated that the HIR was correlated to *Salmonella* infection, which was consistent with experimental outcomes [109].

### 3.3 Dynamic patterns of HIR resulting from *Salmonella* infection

We found four identifiable patterns in simulated HIR. Corresponding changes in the interface of NetLogo simulation were captured. The simulated results and changes in the interface of NetLogo are shown in Figs 3–11.

**Fig 3:**
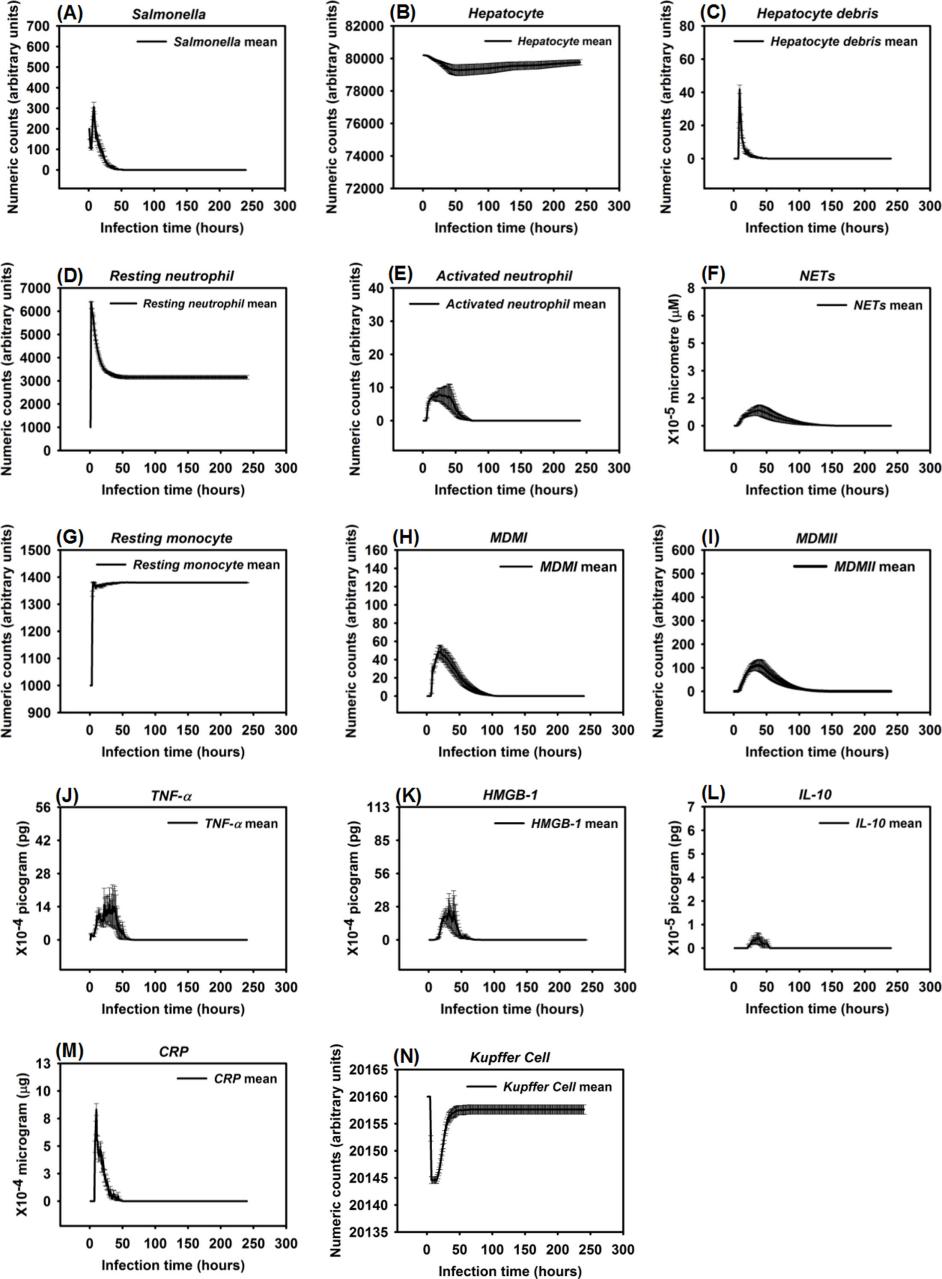
Healing response after *Salmonella* infection (load) of 200. (Mean counts ± SE) of indicators were measured at each simulation time point (replications = 100).

**Fig 4:**
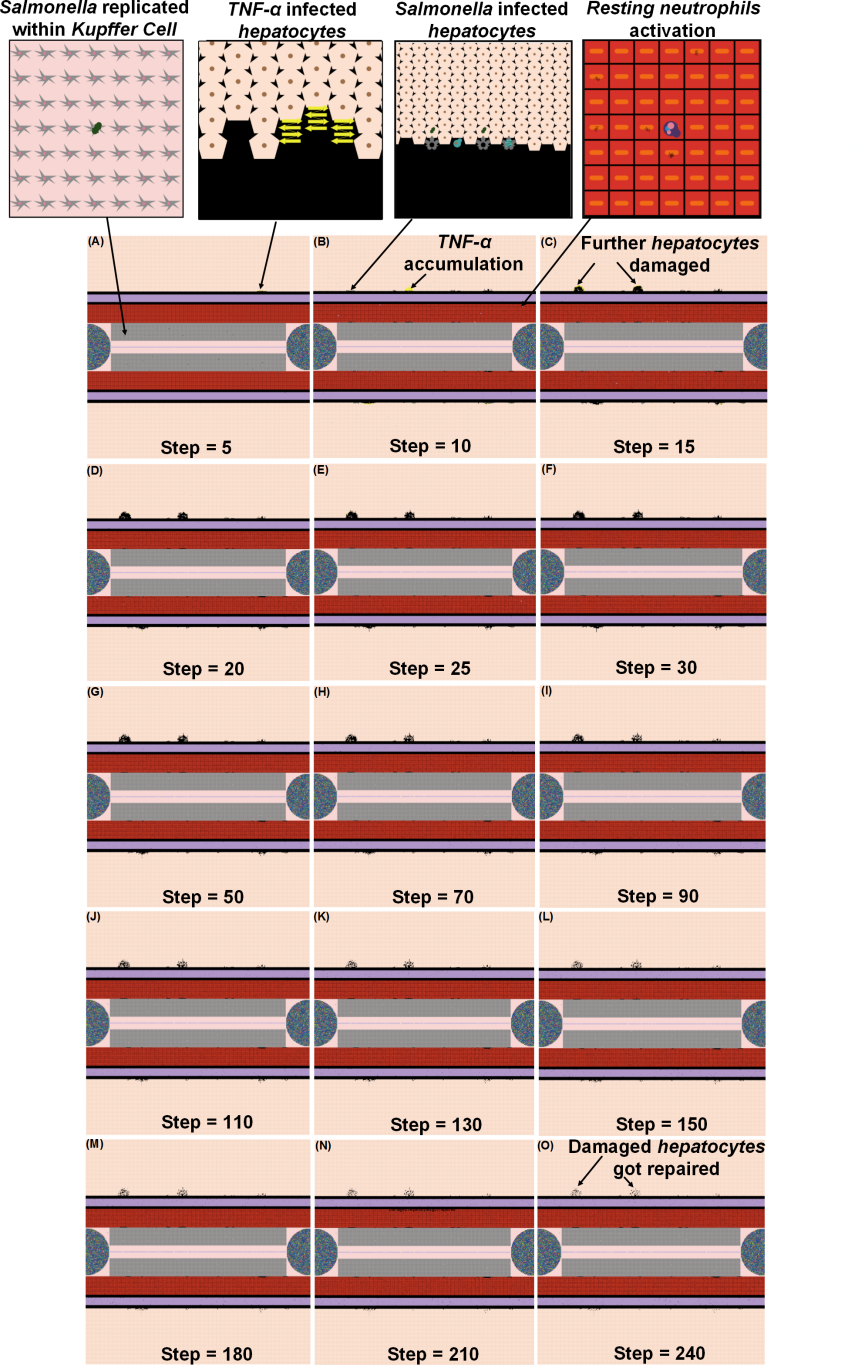
Examples of the NetLogo interface at selected time points (5–240 hrs) after infection with 200 *Salmonella*. Note: 1 step is equivalent to 1 hr. post infection.

**Fig 5:**
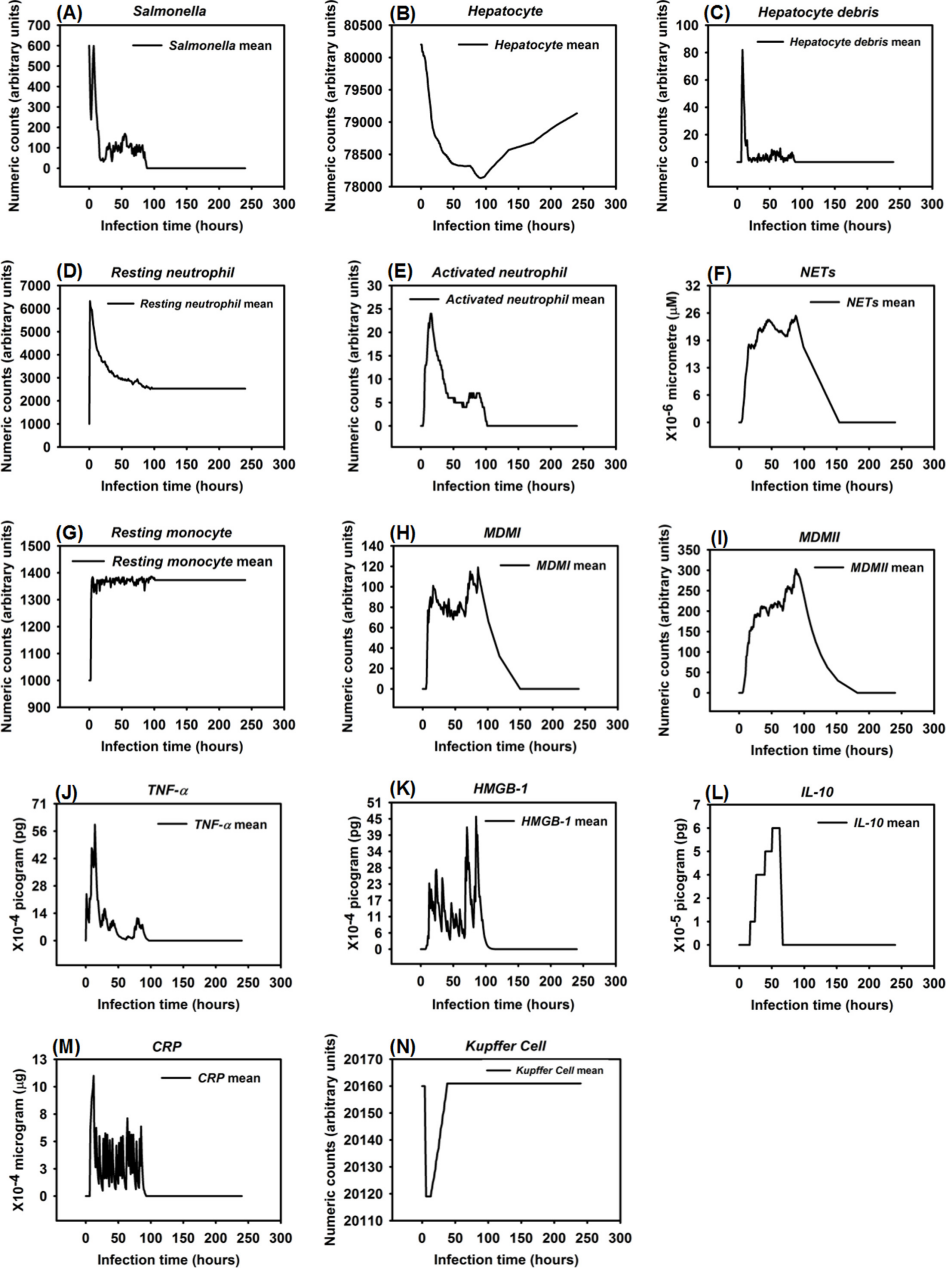
Persistent infection after *Salmonella* infection (load) of 600. Counts of different variables (agents) were measured at each simulation time point of one selected simulation.

**Fig 6:**
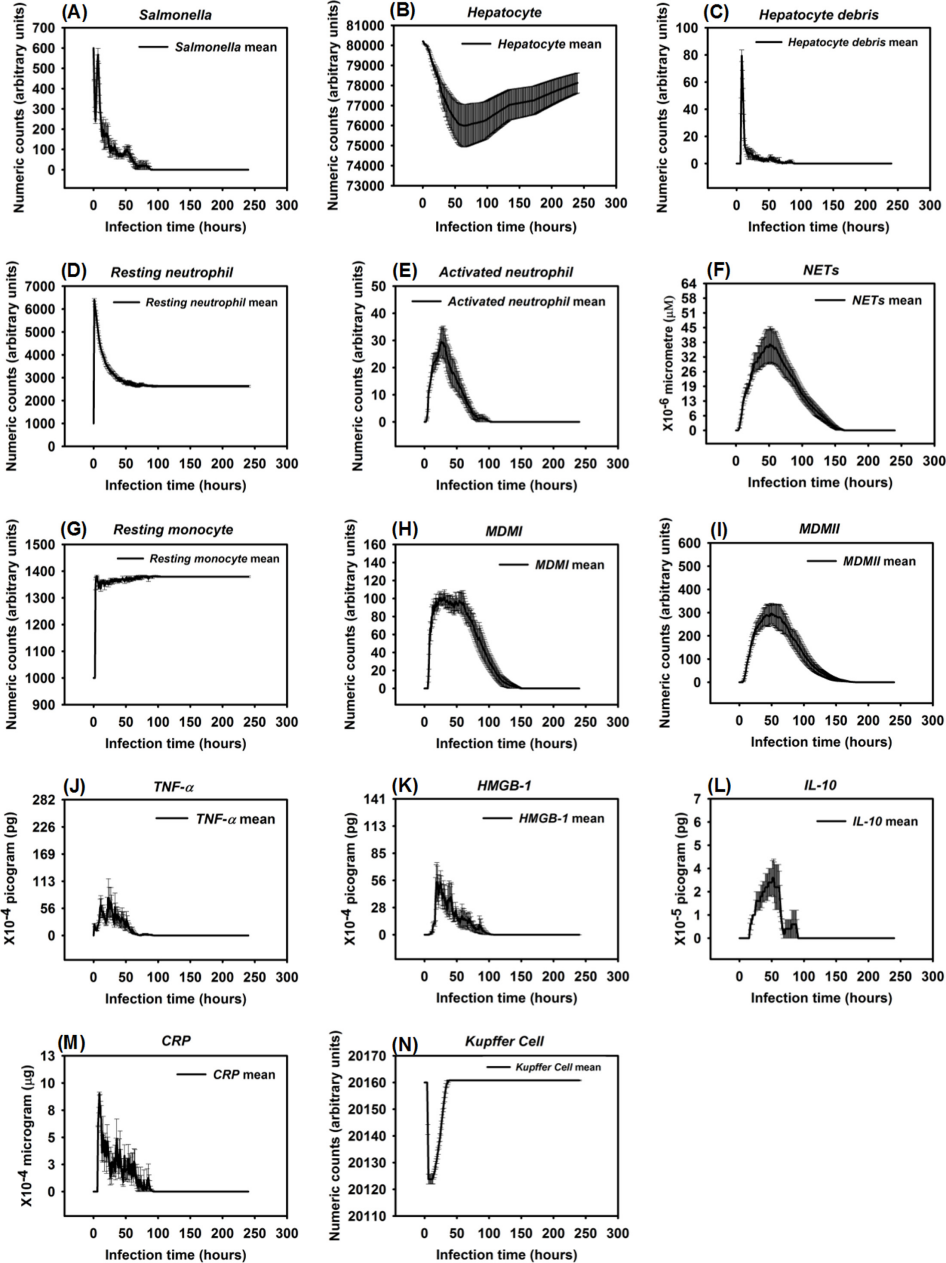
Persistent infection after *Salmonella* infection (load) of 600. (Mean counts ± SE) of different variables (agents) were measured at each simulation time point (replications = 10).

**Fig 7:**
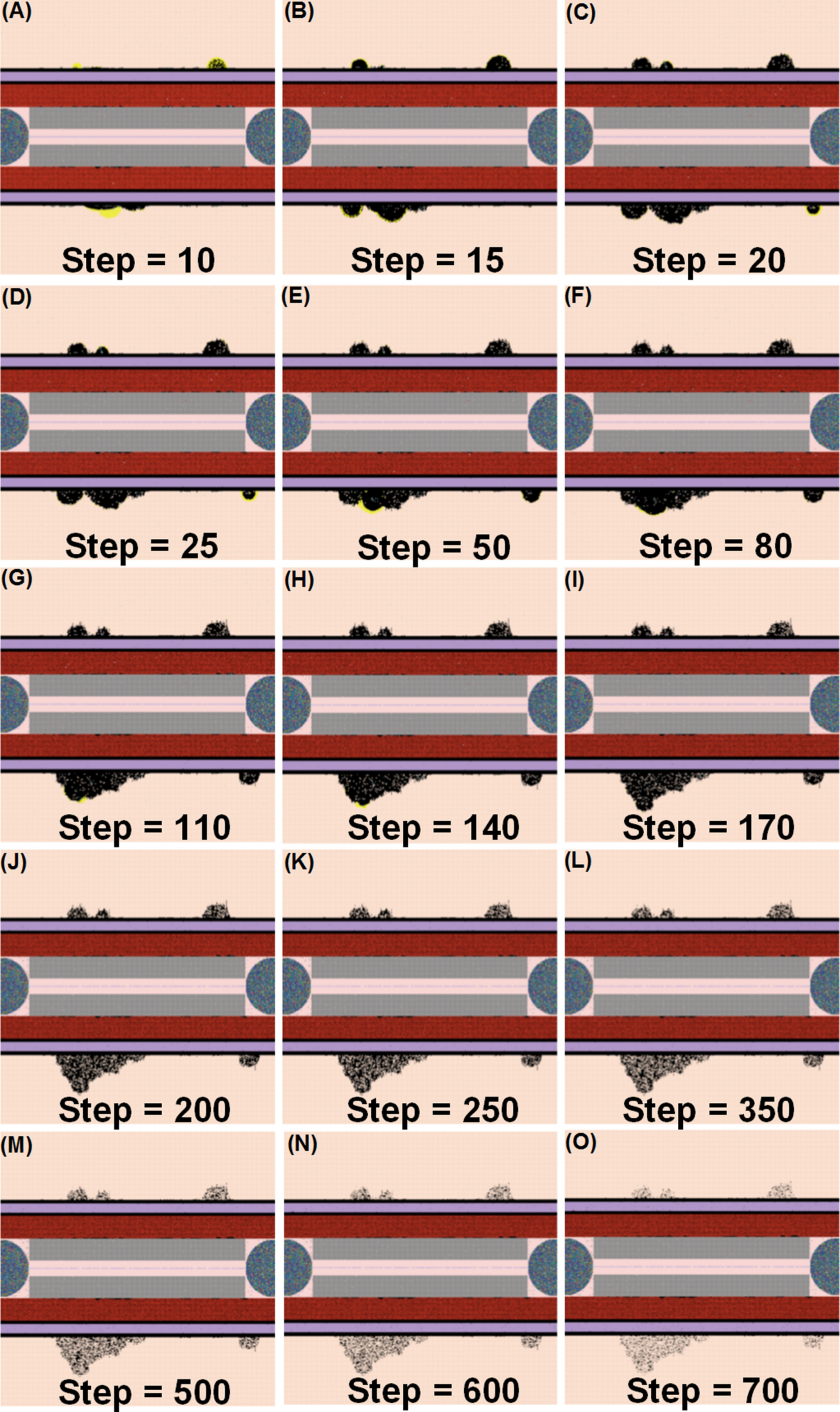
Examples of the NetLogo interface at selected time points (10–240 hrs) after infection with 600 *Salmonella*. Note: 1 step is equivalent to 1 hr. post infection.

**Fig 8:**
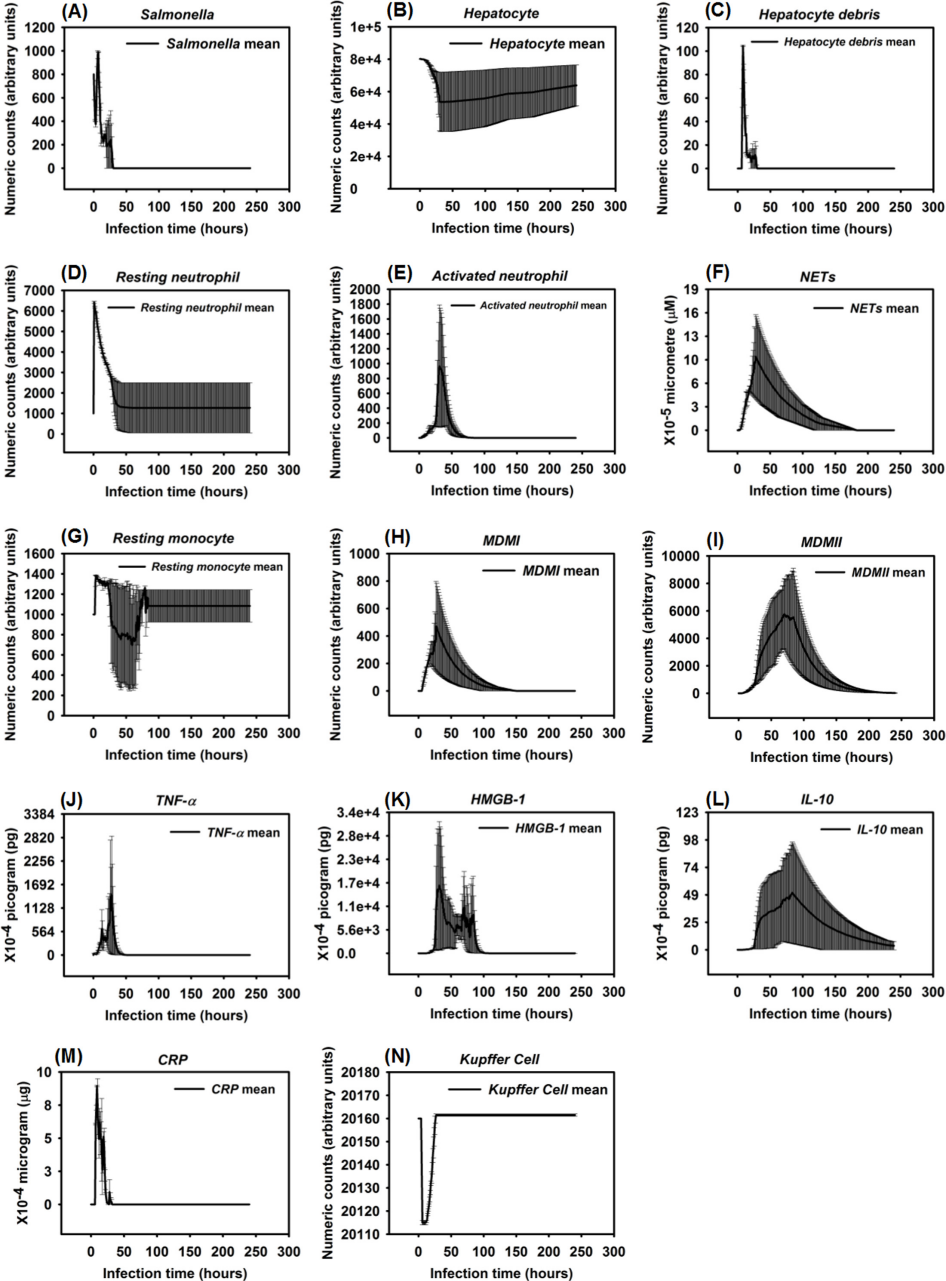
Hyperinflammatory response after *Salmonella* infection (load) of 800. (Mean counts ± SE) of indicators were measured at each simulation time point (replications = 10).

**Fig 9:**
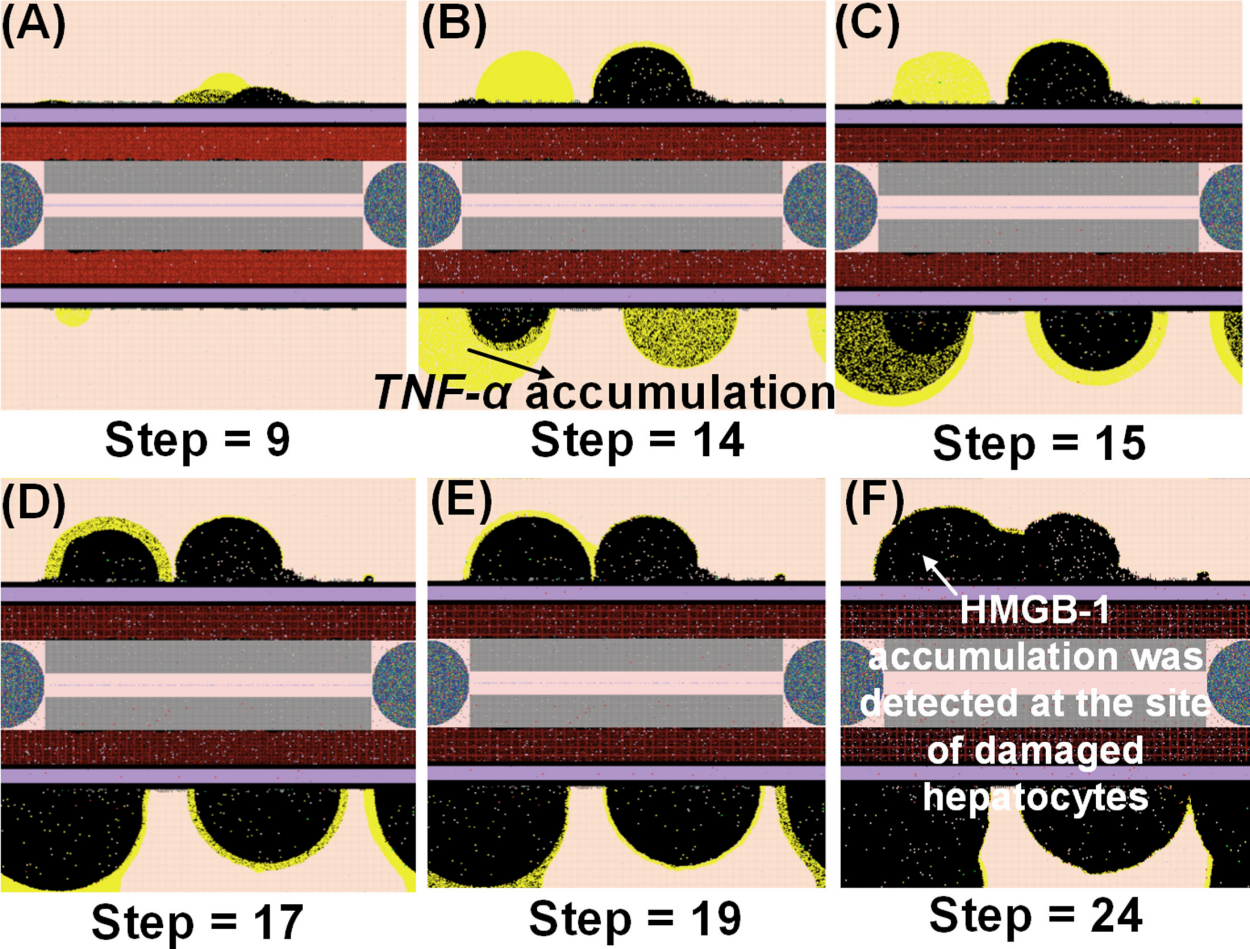
Examples of the NetLogo interface at selected time points (9–24 hrs) after infection with 800 *Salmonella* showing the HMGB1 (green) or TNF-alpha (yellow) that accumulate in situ. Note: 1 step is equivalent to 1 hr. post infection.

**Fig 10:**
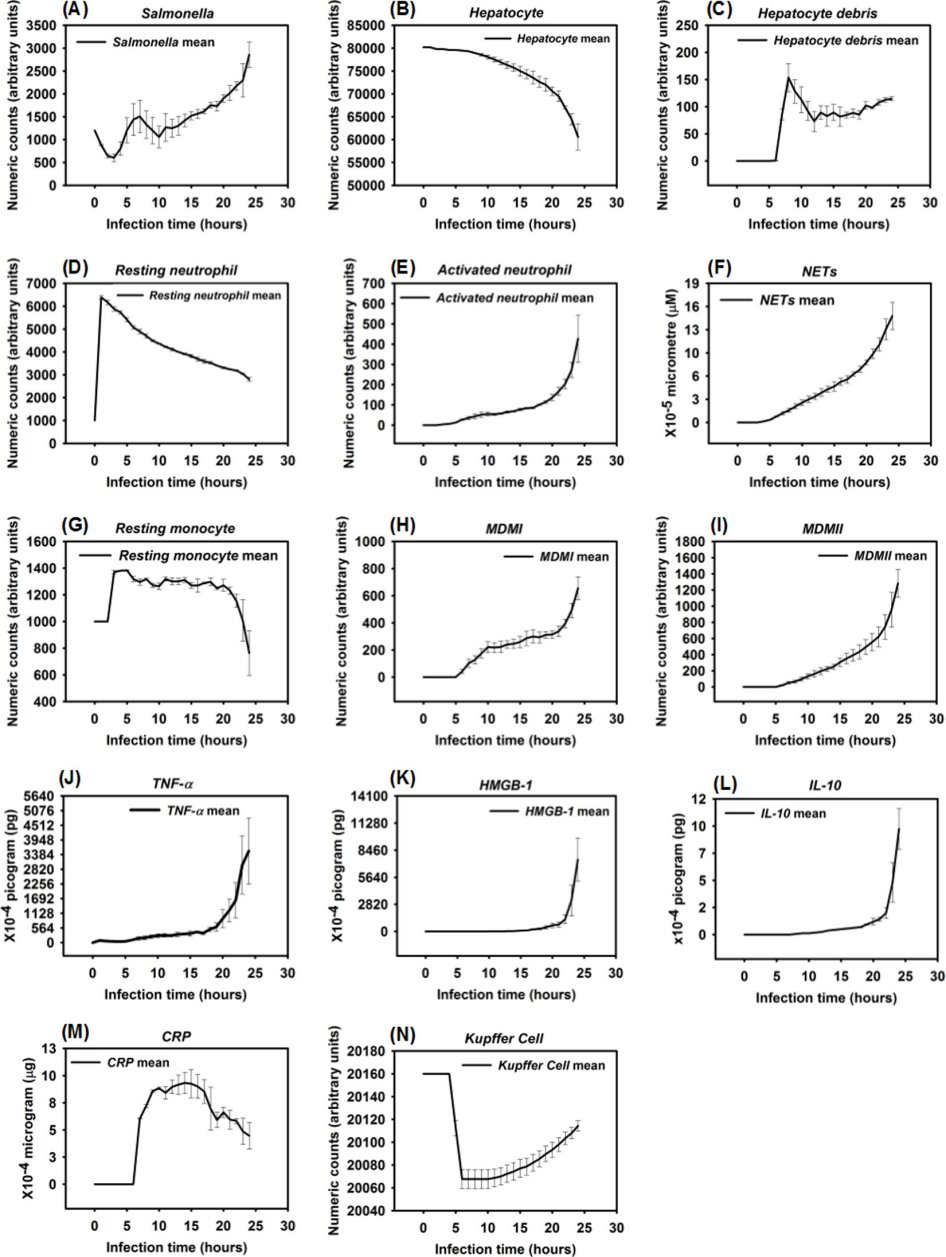
Organ dysfunction after *Salmonella* infection (load) of 1200. (Mean counts ± SE) of indicators were measured at each simulation time point (replications = 10).

**Fig 11:**
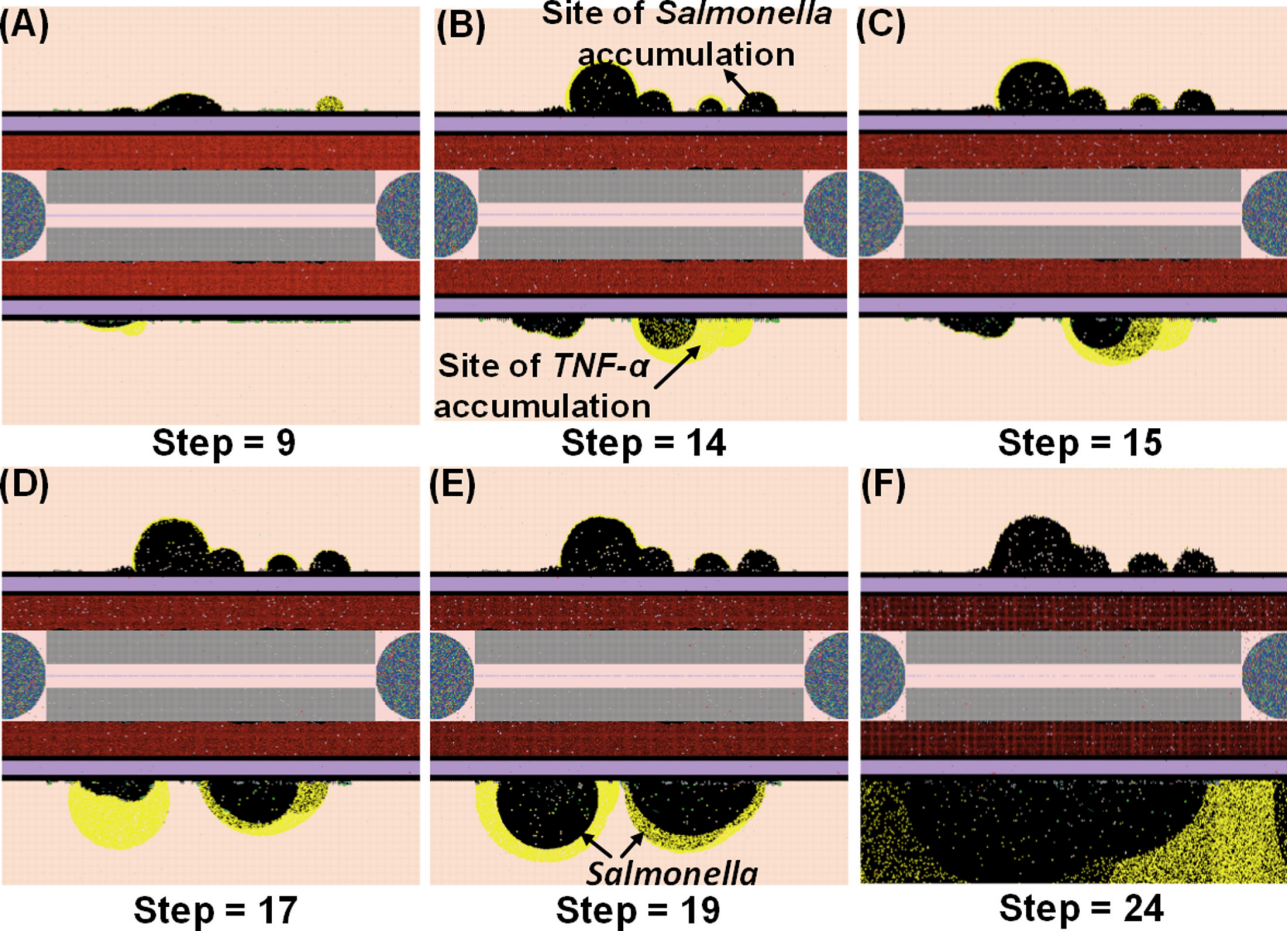
Examples of the NetLogo interface at selected time points (9–24 hrs) after infection with 1200 *Salmonella*. Accumulation of *Salmonella* bacteria (black areas) and TNF-alpha (yellow areas) in *situ*. Note: 1 step is equivalent to 1 hr. post infection.

When the initial infection with *Salmonella* was 200 counts, the number of *Hepatocyte Debris* and *CRP* increased for the first 18 hrs of simulation but then progressively decreased to 0, demonstrating no additional pathology at later stages of the simulation. The *Salmonella* counts, *Activated Neutrophil*, *NET*, *TNF-α*, *HMGB-1*, *MDMI*, and *MDMII* levels in the simulation sharply increased at the beginning of the infection but progressively decreased as the infection progressed. We inferred that this combination of variables is similar to a host curing an infection, so we referred to it as a healing process (Fig 3). We detected that a small number of hepatocytes (less than 0.3% of total hepatocyte counts) were damaged at simulation step 15 (Fig 3B). We also found that only a few neutrophils and monocytes (less than 200 cell counts) were activated when the initial *Salmonella* infection was 200. Ultimately, damaged hepatocytes were replaced with new (healthy) hepatocytes as the simulation proceeded (Fig 4).

Experimental studies in mice have shown early expression of pro-inflammatory cytokines in response to *Salmonella* infection [111]. A comparison of the peak level of *HMGB-1* to the peak level of *TNF-α* reveals that the peak level of *HMGB-1* is higher and that the time required to reach maximum concentrations of *TNF-α* was less than the time required for *HMGB-1* (average of 9 hrs versus 24 hrs post infection) [82, 112, 113]. Our simulated results recapitulated this *TNF-α* and *HMGB-1* pattern. We found that the peak level of *TNF-α* ranged from 1.40 × 10^−3^ to 2.64 × 10^-3^pg. Because we modeled liver dimensions based on the model size (401 × 401 2-D grid), we assumed that *TNF-α* secretion was proportional to the model size and that intensity of *TNF-α* secretion was proportional to the initial challenge of *Salmonella* dose. Under those two assumptions, this response paralleled *TNF-α* levels (160 to 210 pg) found in a mouse model responding to 10^7^ CFU *Escherichia coli* (a medium dose) [31, 82]. Similarly, the kinetics and amounts of secreted *HMGB-1* correlated with the peak level of an *HMGB-1* response seen in experimental observations if model size was taken into account [112]. We observed that the increase in *HMGB-1* levels began later in our model compared to production kinetics observed in *in vitro* stimulation assays [112], However, kinetics of our model were consistent with the delayed contribution *HMGB-1* is proposed to have during sepsis [114].

Recruitment of monocytes to the liver rose sharply around 24 hrs after infection in our model, which is consistent to approximately 1 day in an actual experimental system [115]. There was approximately a 50% *Salmonella* decrease within 6 hrs after initiation of HIR (Fig 3A), paralleling kinetics previously observed in mice [116]. During actual infections, the decrease in bacterial load correlated with the influx of neutrophils [116]. We observed a similar trend in the simulation (Fig 4). We used *CRP* levels and *Hepatocyte Debris* to reflect the level of tissue damage that occurred after infection. *CRP* is released by the liver in response to stress, infection, and/or damage [52, 54, 117], and the debris simulates dead and dying hepatocytes. Our simulated results showed that *CRP* rose initially after infection, but *CRP* concentration fell sharply after the infection was cured as part of the “Healing Response”. A similar pattern of *CRP* concentrations was identified in healthy patients infected by bacteria in clinical cases [118].

In some simulation replications, when the initial *Salmonella* infection was 600, the outcome more closely resembled a persistent infection, defined as the state in which *Hepatocyte Debris*, *CRP*, and *Salmonella* levels initially declined but subsequently increased to much higher levels before the infection was resolved at approximately 90 hrs. (Fig 5). Under this condition, *Activated Neutrophil* numbers declined along with the decline in bacterial numbers and *NET* values did not return to baseline for approximately 50 more hrs. We also observed oscillations in levels of cytokine mediators *TNF-α* and *HMGB-1* as the infection was resolved (Fig 5). Moreover, this resolution correlated with oscillating *Salmonella* numbers during the waning 25 to 60 hrs of the infection. Others have observed oscillatory patterns in host responses to other types of bacteria in mouse infections [119]. Therefore, we were reassured that the simulation captured the essence of a real infection. The *CRP* pattern during persistent infection (Fig 6) was significantly distinct from the *CRP* pattern observed in the healing response (Fig 3). As shown in Fig 6, the *CRP* level rose initially after the infection and waxed and waned for another 2 to 3 days. On the 4^th^ day after infection, *CRP* levels diminished sharply and damaged hepatocytes began their recovery, similar to the *CRP* pattern reported in a clinical study [118].

Detectable hepatocyte damage began at simulation step 10 (10 hrs post infection), and a significant increase in hepatocyte damage was observed beginning at stimulation step 15 (15 hrs post infection). Hepatocyte damage was persistently observed for 7 days. As the persistent infection proceeded, a large area of hepatocyte damage, which would translate to liver damage in an animal model, was observed (Fig 7). Our simulated results paralleled hepatocyte damage seen *in vivo* after experimental infections where recovery (or “healing”) of hepatocytes was detected after 7 days and continued for approximately 30 days [120]. These data are consistent with the idea that a persistent infection will induce a higher mortality rate compared to a healing response because acute tissue damage is more detrimental to the host (Fig 6B). Remarkably, we observed that oscillations in agent counts were damped when calculated mean values of the agent counts for simulation replications (Fig 6). The oscillations we observed in a single simulation run (Fig 5) of IMMABM indicate that the individuals with persistent infection could have identifiable oscillated patterns during HIR.

HIR could also result in a pattern we termed as a hyperinflammatory response (Figs 8 and 9). During this type of response, *Salmonella* counts dropped within the first 24 hrs of HIR (Fig 8A). However, a significant elevation in phagocytic cells (Fig 8E, 8H and 8I) and inflammatory cytokines was observed (Fig 8J, 8K and 8L) compared to the healing and the persistent infection responses, causing severe hepatocyte damage that could lead to death (Fig 8B). Interestingly, we observed that the ranges of agent counts in the hyperinflammatory response (Fig 8) were more variable compared to the healing and persistent infection responses (Figs 3 and 6). This made it difficult to accurately predict outcomes in this type of HIR. However, we suggest that when the mean values of *TNF-α*, *HMGB-1* and *IL-10* exceeded the mean values identified in the hyperinflammatory response (Fig 8J, 8K and 8L), this serves as a warning signal of HIR progression to a hypothetical death status as the simulation proceeded. In a few simulation replications, we observed that all the hepatocytes were killed or damaged (*Hepatocyte* count = 0) within the first 48 hrs of infection during HIR characterized as hyperinflammatory (data not shown). These data suggest that a hyperinflammatory response could lead to a higher mortality rate compared to a persistent infection because of the acute and severely damaged hepatocytes observed.

The last pattern of HIR that we observed was characterized by progressively increasing *Salmonella* counts. Under this condition, *Salmonella* and inflammatory cytokines continued to rise as the simulation proceeded. Therefore, we classified the combined pattern of increasing *Salmonella* counts and inflammatory cytokine counts (*TNF-α*, *HMGB-1*, and *IL-10*) as “organ dysfunction”, as shown in Figs 10 and 11. Organ dysfunction appeared to be so problematic because in HIR the liver contained less than 1/4 of the healthy hepatocytes after 24 hrs compared to the number present at the time of the initial infection (Fig 10). Specifically, the simulation stopped under the condition that no more healthy hepatocytes existed. We only calibrated the data of organ dysfunction for the first 24 hrs of HIR because healthy hepatocytes died out at 24 hrs of simulation in some replications. According to our simulations, a sign of organ dysfunction might be characterized by continued increases in *Salmonella*, *TNF-α* and, *HMGB-1* counts and continued high levels of CRP. The acute rise and a slow decrease in *CRP* levels observed in our model were consistent with CRP concentration patterns identified in patients with septic shock [121]. This adds validity to the simulated results from our IMMABM.

To conclude, we found that a healing response, where *Salmonella*, other phagocytic cells, and inflammatory cytokines quickly fell below threshold levels, was more likely to occur when the initial *Salmonella* load was low. We identified a persistent infection pattern if inflammatory responses were active (characterized as when *Salmonella* and inflammatory cell levels oscillate during infection). However, if the initial *Salmonella* load was high, a hyperinflammatory response or organ dysfunction was most likely to occur, leading to the death of infected individuals. In addition, when these simulated results were compared to experimental data, the simulations paralleled indicator patterns reported in actual mouse experiments [112, 115, 116, 118–120, 122]. It also became clear that predicting a final outcome from the emerging dynamic patterns of HIR became more difficult when initial *Salmonella* loads were above 500 counts (See Section 3.4).

### 3.4 Outcome assessment

**Fig 12:**
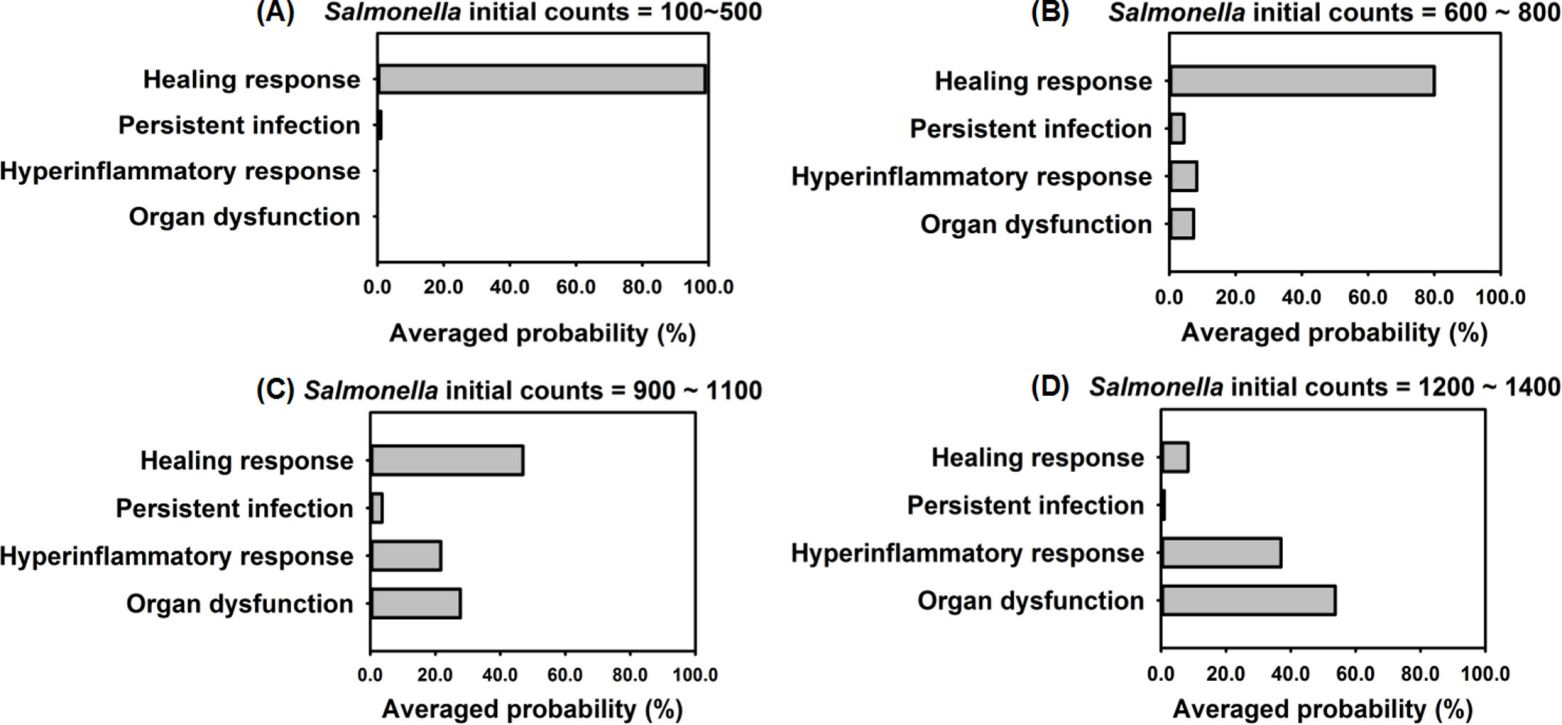
Probabilities of leading to healing response, persistent infection, hyperinflammatory response, and organ dysfunction when the *Salmonella* initial loads range from 100 to 1400 counts. This experiment excluded adaptive immunity in IMMABM (i.e. excluded CD4+ T cells, CD8+ T cells, B cells, and antibody).

To examine how the model behaved when parts of the immune response were absent we ran IMMABM simulations with and without acquired immunity using initial *Salmonella* doses ranging from 100 counts to 3200 counts. We thought that this would be an effective test of the model since we hypothesized that the absence of acquired immunity would negatively impact the host’s ability to heal. In these experiments, doses were increased in increments of 100 counts with 100 replications per dose for a total of 4500 replications in the IMMABM. In the absence of acquired immune components, HIR outcomes clearly skewed toward a healing response at doses less than 500 counts. However, as the initial *Salmonella* doses increased, it became clear that the dynamic patterns of HIR could diverge in the health outcomes (healing response *vs*. persistent infection *vs*. hyperinflammatory response *vs*. organ dysfunction). For example, when the initial *Salmonella* load was 800 counts, all four dynamic patterns of HIR could emerge. Nevertheless, when initial *Salmonella* counts were below 500, the healing response was identified over 98% of the time. However, when the initial *Salmonella* count exceeded 1300 counts, only hypothetical death status (hyperinflammatory response or organ dysfunction) was identified from IMMABM simulations. In order to compare potential survival and mortality rates of HIR under various initial *Salmonella* challenge loads, we generated a probability histogram that ended with the healing response, persistent infection, hyperinflammatory response, or organ dysfunction of HIR against various *Salmonella* initial loads (Fig 12). The probability of HIR ending in each possible outcome clearly changes as the dose increases from 100 counts to 1400 counts (Fig 12).

**Fig 13:**
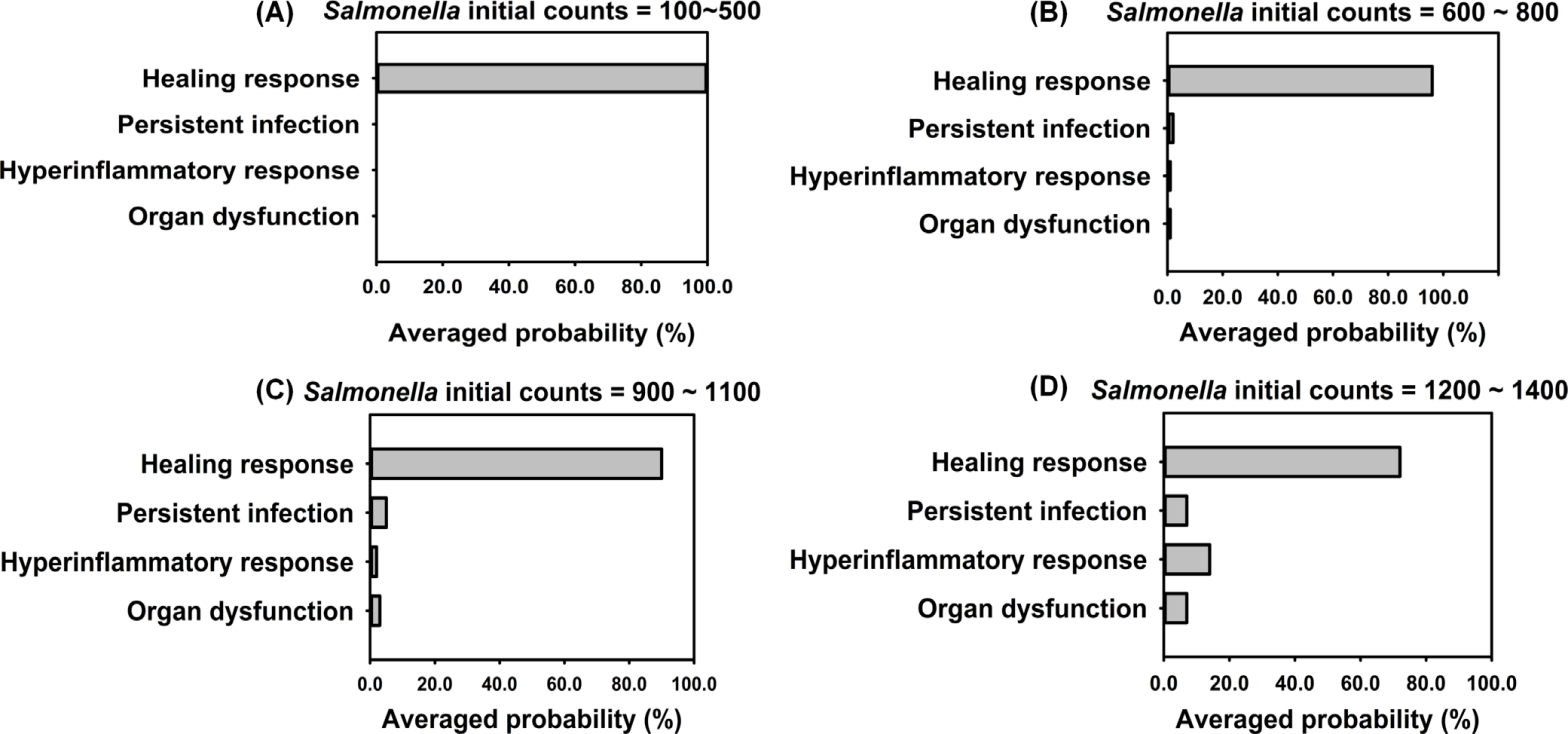
Probabilities of leading to heading response, persistent infection, hyperinflammatory response, and organ dysfunction in the presence of Acquired Immunity. *Salmonella* initial loads range from 100 to 1400 counts.

When CD4+ T cells, CD8+ T cells, B cells, and antibody were added to IMMABM, there continued to be a dose response as seen in Fig 12. These data show the importance of innate immunity in the control of *Salmonella* infections [123, 124]. However, the likelihood of organ dysfunction dropped compared to when acquired immune components were missing (Fig 13). It took substantially higher challenge doses to induce more severe infections (S2 Fig). Therefore, the model seemed to accurately reflect the relative contributions of innate and acquired immunity during *Salmonella* infections [39, 125, 126].

As *Salmonella* initial loads increased from 1800 to 3200, the chance of a hyperinflammatory response was significantly higher than organ dysfunction (S2 Fig). Although both conditions can be detrimental to the host, the model reflects subtle changes in the infection and the rapid and overwhelming pro-inflammatory response induced by a high initial loads of *Salmonella*. Interestingly, the overwhelming pro-inflammatory response damages hepatocytes at an early stage of HIR results in few *Salmonella* replications within hepatocytes. It is also interesting to note that in other Gram-negative bacterial infections, the absence of T cells results in prolonged neutrophila (hyperinflammtion) in the lung [2]. Again, suggesting that the model is beginning to reflect the *in vivo* situation.

The experimental data and stochastic processes embedded in IMMABM were essential to a computational simulation because an HIR is an inherently stochastic process. Experimental data show that cellular and soluble mediator interactions and concentrations change and their levels are dependent on location and time. For example, the *Salmonella* killing rate by one neutrophil can range from 2.94 to 12.94 *Salmonella*/per neutrophil/hr according to a human model [51]. The data illustrated in Figs 12 and 13 suggests that IMMABM was able to capture the stochastic nature of the host response during HIR by showing that interactions among agents and the outcomes of the simulations varied for each run. Consistent with its embedded stochastic nature, the IMMABM allowed us to determine the probability of each possible outcome in individuals, thereby allowing reasonable predictions of HIR outcomes. For example, the simulated results in Figs 12 and 13 demonstrated as *Salmonella* initial loads increased, the probability increased that HIR would end with hyperinflammatory response or organ dysfunction. In contrast, lower initial challenge doses were more likely to be identified as healing response or persistent infection.

### 3.5 Biomarkers of HIR

As described in Section 3.3, *HMGB-1* and *CRP* emerged as biomarkers for HIR because their expression patterns closely correlated to HIR outcomes. Similar to our simulated results, persistent elevation of *HMGB-1* and *CRP* was also observed in experimental studies [121, 127, 128].

**Fig 14:**
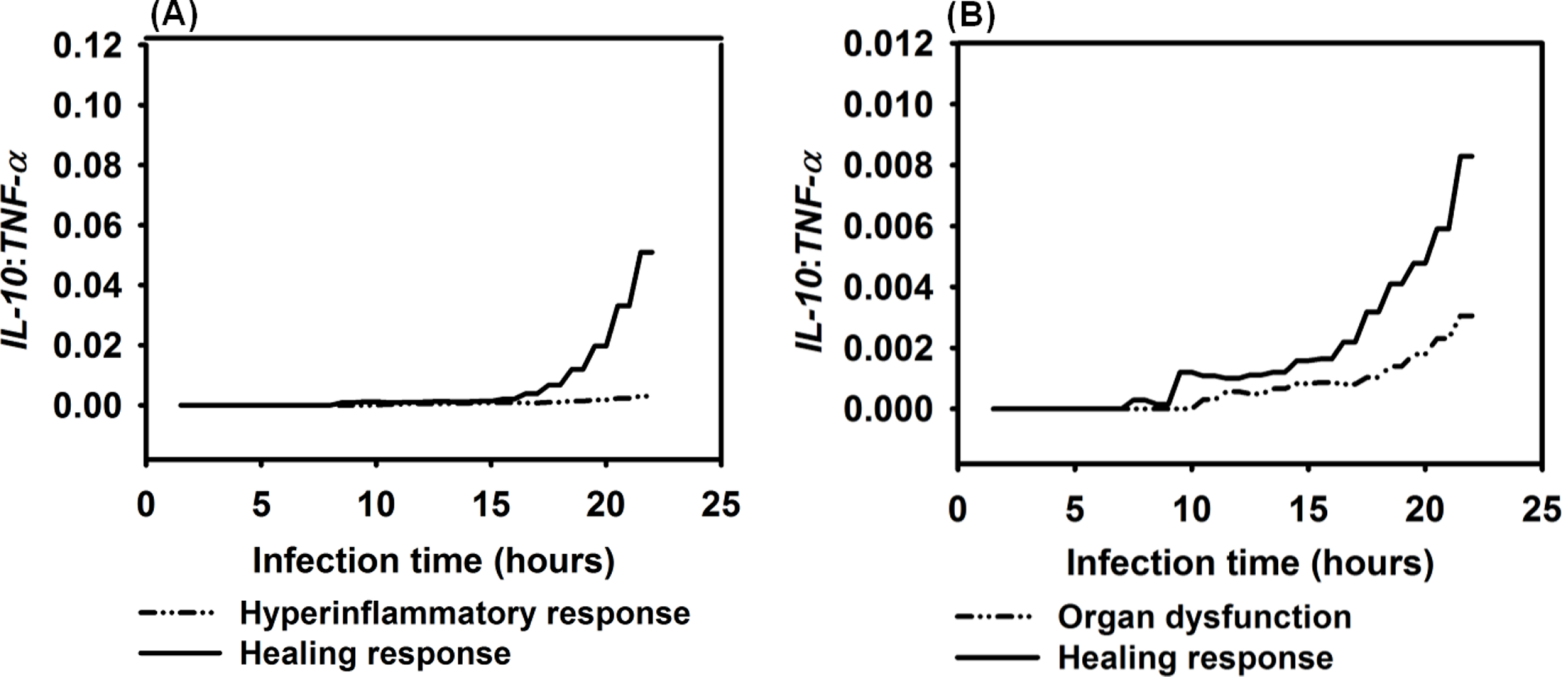
Comparison of *IL-10*: *TNF-α* ratio among healing response, hyperinflammatory response, and organ dysfunction responses *vs*. infection time. Mean values of *IL-10*: *TNF-α* ratios were measured at each simulation time point (replications = 10).

In clinical practice, the *IL-10*: *TNF-α* ratio is one recommended biomarker used to monitor the progression of sepsis [129, 130]. Therefore, we calculated the average *IL-10*: *TNF-α* ratio for a healing response, a hyperinflammatory response, and organ dysfunction during infection in 10 simulation runs. Results in Fig 14 show that the average *IL-10*: *TNF-α* ratios in both the hyperinflammatory and organ dysfunction were significantly lower compared to the healing response (*P* = 0.0061 and *P* = 0.0152, respectively, using the Mann-Whitney *U* tests). Therefore, the *IL-10*: *TNF-α* ratio in the IMMABM accurately captured the elevated *IL-10*: *TNF-α* ratio associated with patients’ healing process [129].

**Fig 15:**
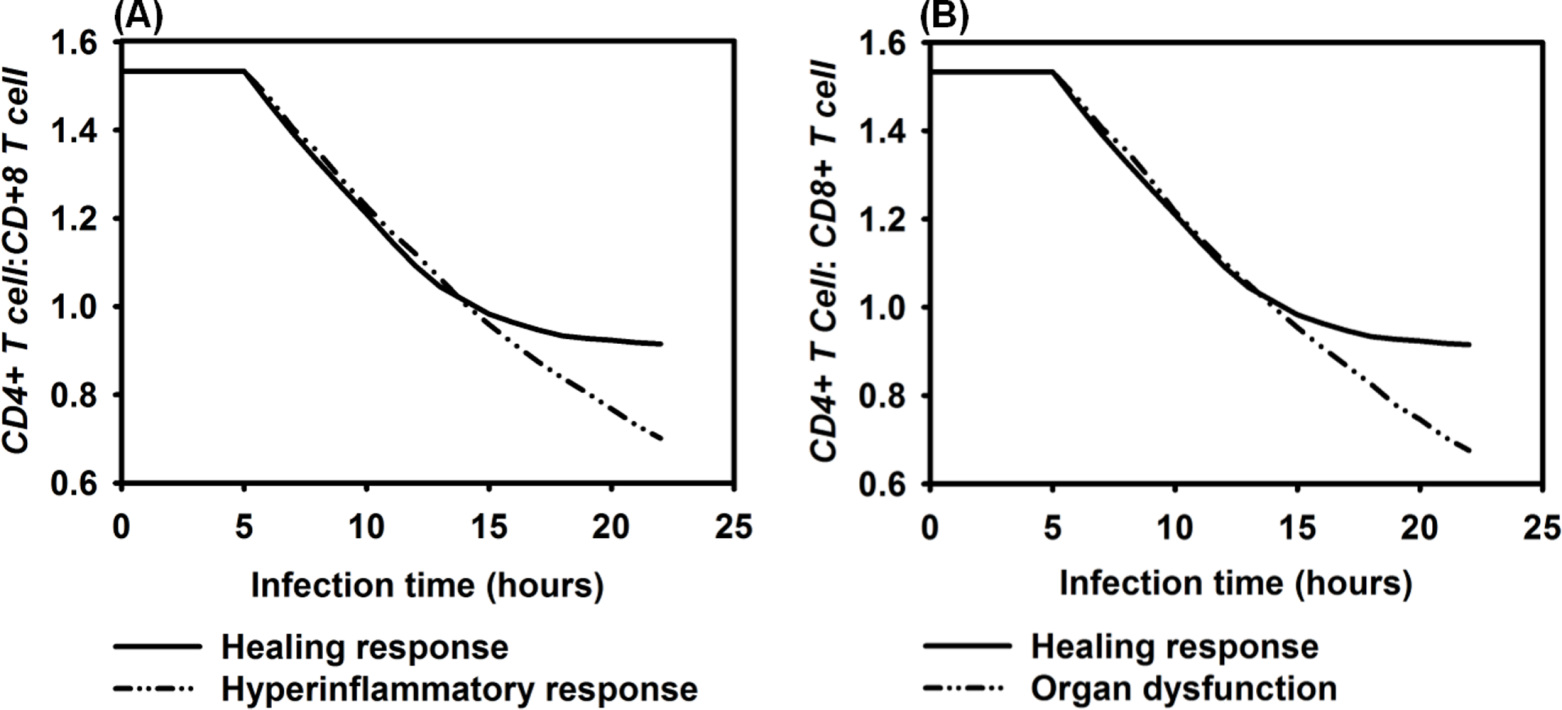
Comparison of *CD4+ T Cell*: *CD8+ T Cell* ratio among healing response, hyperinflammatory response, and organ dysfunction response *vs*. time after infection. Mean values of *CD4+ T cell*: *CD8+ T cell* ratios were measured at each simulation time point (replications = 10).

The ratios of *CD4+ T cell*: *CD8+ T cell* are relatively lower in patients with sepsis, compared to non-sepsis patients [131, 132]. The results in Fig 15 show that in IMMABM there was a significantly lower *CD4+ T cell*: *CD8+ T cell* ratio when the HIR progressed to hyperinflammatory (*P* = 0.0083) or organ dysfunction responses (*P* = 0.0041) after 15 hours of infection. Therefore, this basic clinical measure of T cell response [131, 132] also appears to be accurately reflected in the IMMABM.

**Fig 16:**
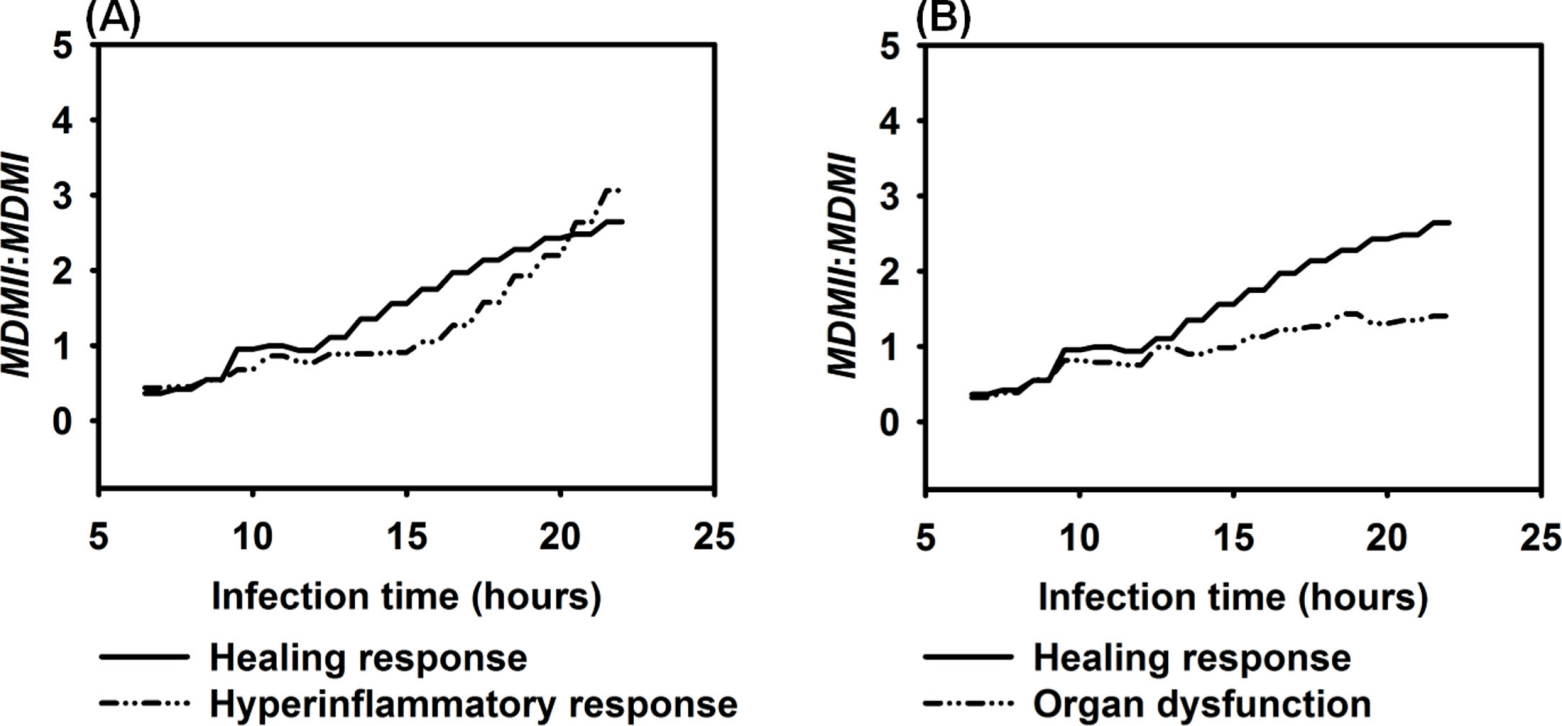
Comparison of *MDMII*: *MDMI* ratio among healing response, hyperinflammatory response, and organ dysfunction response *vs*. infection time. Mean values of *MDMII*: *MDMI* ratios were measured at each simulation time point (replications = 10).

Our simulated results showed that the *MDMII*: *MDMI* ratio was less significantly correlated to the outcomes of HIR compared to the *IL-10*: *TNF-α* ratio and the *CD4+ T cell*: *CD8+ T cell* ratio. During the healing response, the *MDMII*: *MDMI* ratio was not significantly higher (*P* = 0.2623) than the ratio during hyperinflammatory response but it was significantly elevated (*P* = 0.0019) compared to the ratio in organ dysfunction, as shown in Fig 16. Although it is clear that *MDMI* polarization is common in bacterial infections [133], it is less clear if macrophage polarization is associated with host dysfunctional responses. Therefore, it is possible that our simulated data reflect the *in vivo* ambiguity. Alternatively, *MDMII*: *MDMI* ratio may not be appropriate in the liver compartment. Refinement of the model will be necessary to help resolve this. In spite of this, the IMMABM in its current format, has identified some biomarkers that reflect the *in vivo* situation (Table 1). This suggest that the IMMABM is beginning to function in a useful manner by paralleling actual host responses.

**Table 1:** Relationship between dynamic patterns of hepatic inflammatory response and dynamic patterns of essential biomarkers in IMMABM.

Dynamic patterns of hepatic inflammatory response	Dynamic patterns of *CRP*	Dynamic patterns of *HMGB-1*	*IL-10*: *TNF-α* (ratio)	*CD4+ T cell*: *CD8+ T cell* (ratio)
**Healing Response**	Sharply increase and smoothly decay	Smoothly decay	Low	High
**Persistent Infection**	Oscillating decay	Oscillating decay	Medium	High
**Hyperinflammatory Response**	Sharply increase and smooth decay	Significantly elevated and decay	High	Low
**Organ Dysfunction**	Sharply increase and slow decay	Significantly elevated	High	Low

### 3.6 Therapy-directed experiments *in silico*

**Fig 17:**
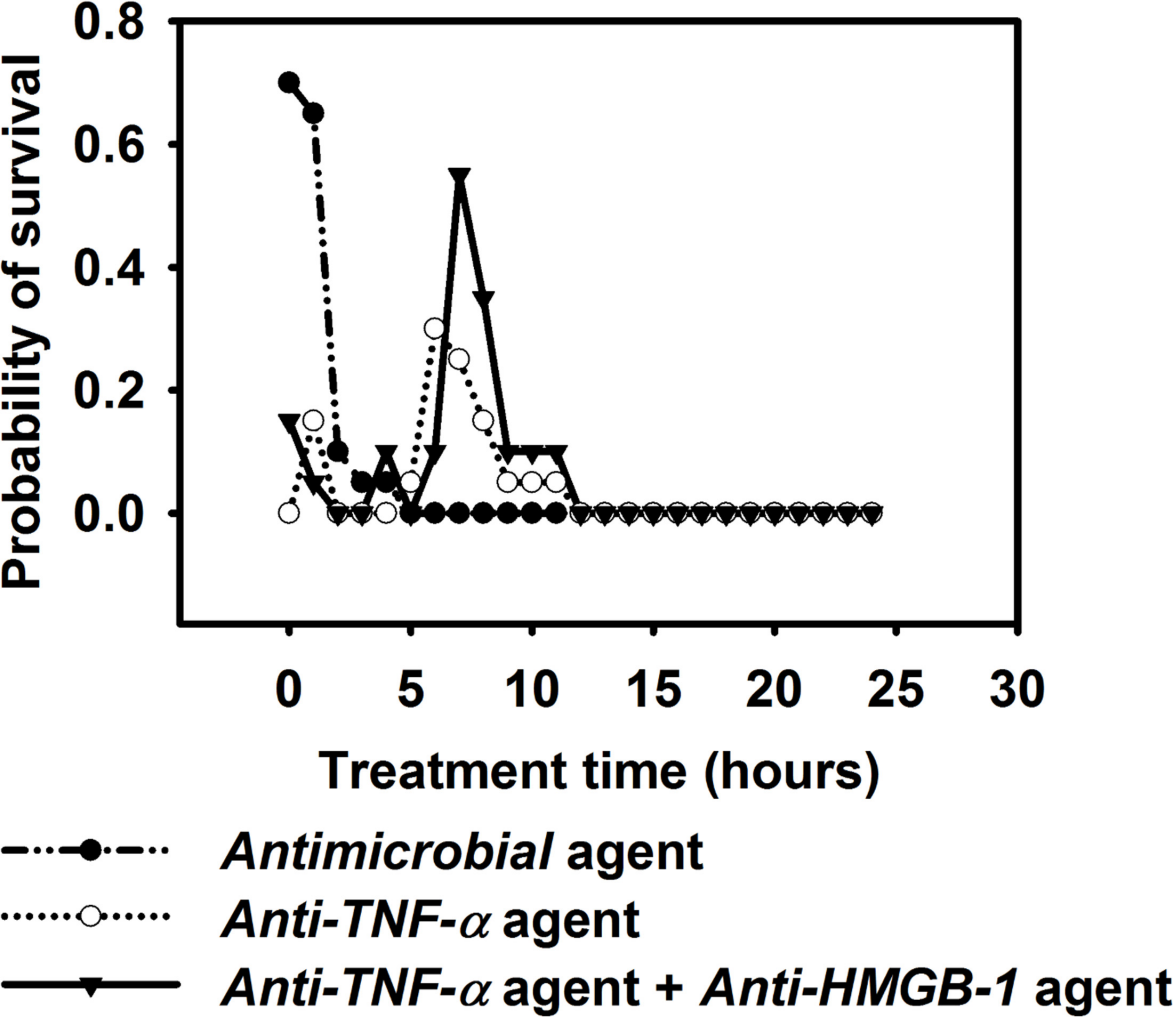
Assessment of therapy in IMMABM. **Hypothetical *antimicrobial* agents, *anti-TNF-α* agents, and a combination of *anti-TNF-α* and *anti-HMGB-1* agents were administered to determine their impact in HIR.** The “Probability of survival” label represents the probability of HIR ending with a healing response. We assume 1 *antimicrobial* agent kills 1 CFU *Salmonella*, 1 *anti-TNF-α* agent degrades 2.82×10^−5^ pg *TNF-α*, and 1 *anti-HMGB-1* agent degrades 2.82×10^−5^ pg *HMGB-1*. 200 *antimicrobial* agents, 1000 *anti-TNF-α* agents, or 800 *anti-HMGB-1* agents were incorporated each time in corresponding experiments. The administration of the treatment therapies was done one time in IMMABM starting at 0 hour to 24 hours (abscissa). 20 simulation replications were conducted for each treatment regimen (1500 simulation replications were conducted for this experiment).

We designed an experiment using a hypothetical antimicrobial agent (i.e. an antibiotic that could kill Salmonella), an anti-TNF-α agent (i.e. an antibody therapy), and a combination of anti-HMGB-1 agent and anti-TNF-α agent. We incorporated these treatments into the IMMABM, and compared the effects of the three hypothetical treatments to the outcomes of HIR. Simulated data showed that the treatment effectiveness was highly correlated with treatment start time during the simulation (Fig 17). Specifically, antimicrobial agents caused significant improvement in the survival rates when started during the first hour after infection (beginning of HIR). Interestingly, current recommendations are to administer appropriate antibiotics within 1 hour of a diagnosis of severe sepsis or septic shock [134, 135]. In contrast, the optimal treatment window for anti-TNF-α agents was between 6 hours and 8 hours after infection (Fig 17), which may explain why anti-TNF-α treatment did not effectively improve survival for patients in some clinical studies [136, 137]. The combination of anti-HMGB-1 and anti-TNF-α was more effective in improving the survival rates when treatment was started between 7 hours and 11 hours after infection, compared to using only anti-TNF-α (Fig 17).

Our simulated results demonstrated that effectiveness of anti-agent treatments has a specific time window. Administration of the anti-TNF-α and/or anti-HMGB-1 at an early stage could interfere with the further recruitment of phagocytes. This could lead to organ dysfunction because there would be insufficient numbers of phagocytes to ingest and kill intruding *Salmonella*. In contrast, administration of anti-TNF-α and/or anti-HMGB-1 at a later stage could result in an overwhelming hyperinflammatory response because TNF-α and HMGB-1 levels remained persistently elevated (Figs 8 and 10). A persistent elevation of TNF-α and HMGB-1 can induce a further infiltration of neutrophils and macrophages to the site of infection, which cause an uncontrolled HIR. This could explain why an *anti-HMGB-1* agent and/or an *anti-TNF-α* treatment become less effective as the infection proceeds. We used a fixed amount of *anti-TNF-α* and *anti-HMGB-1* agent for our treatment, which fails to compromise an elevated level of TNF-α and HMGB-1 at a later stage of infection. Furthermore, the chance of HIR ending with a hyperinflammatory response or organ dysfunction was higher as the initial loads of *Salmonella* increased (Figs 12 and 13). This indicates that higher amounts of anti-agents could be necessary during treatment as *Salmonella* infection levels increase.

The insights provided by the therapy-directed experiment suggest that various doses of anti-agent treatment (e.g. increase doses of anti-agent as the infection proceeds) at selected time points could improve the survival rates for septic individuals. Moreover, these data suggest additional experimental directions for the development of effective treatment bundles in experimental animals and/or in preclinical trials.

## 4. Discussion

This paper describes an IMMABM developed to simulate HIR in a mouse infected with *Salmonella*. The IMMABM described interactions between selected agents as a representation of a HIR during *Salmonella* infection and required the understanding of key cellular and molecular processes of HIR at the tissue level. Most importantly, the IMMABM was validated through a series of comparisons between simulated results and experimental studies.

Four distinct dynamic patterns (healing response, persistent infection, hyperinflammatory response, and organ dysfunction) were identified during the IMMABM simulation. One significant finding from the simulations was that the outcomes of a HIR were highly correlated to the initial *Salmonella* counts. When the initial *Salmonella* counts were below 900, hepatic infection had 97% probability to develop into a healing response during 100 simulation runs (S2 Fig). When the initial *Salmonella* counts were between 1000 and 3200 counts, the outcomes of HIR were uncertain (S2 Fig). As the initial counts of *Salmonella* increased, HIR had a higher probability to end with hyperinflammatory or organ dysfunction responses. Furthermore, *CRP*, *HMGB-1*, the *IL-10*: *TNF-α* ratio, and the *CD4+ T cell*: *CD8+ T cell* ratio emerged as biomarkers during HIR. If *CRP* and *HMGB-1* were persistently elevated, HIR was more likely to end in a hyperinflammatory or organ dysfunction response. If the *IL-10*: *TNF-α* ratio or *CD4+ T cell*: *CD8+ T cell* ratio dropped significantly during HIR, a hyperinflammatory or an organ dysfunction response would occur. In the therapy-directed experiment, we observed that antimicrobial intervention significantly improved the survival rates during the first hour. Anti-TNF-α agents and/or anti-HMGB-1 agents improved survival rates when administered at a later stage of infection, and it became clear that efficacy was dependent on time of administration.

### 4.1 Insights into simulated results

This IMMABM began to capture the essence of adaptive immunity during HIR. T cell activation occurs within 24 hrs of HIR *in vivo* [138]. Therefore, we incorporated adaptive immunity, including essential lymphocytes such as CD4^+^ and CD8^+^ T cells, as well as B cells, into our simulation. We found that incorporation of T cells and other acquired immune components could induce changes to the course of the infection. Indeed, in immunocompetent mice, the hyperinflammatory response that we identified in the simulations paralleled observations made during mouse sepsis [138]. In addition, we found that antibodies released during HIR failed to significantly affect organ dysfunction based on the release rate of antibodies and the binding amount of antibody to one *Salmonella* we calibrated [139–141].

During dose response simulations, we observed HIR is correlated to initial loads of *Salmonella*. Furthermore, we calibrated the probability of HIR ending in each possible outcome under a range of initial *Salmonella* loads (Figs 12 and 13). Recent studies found that a high bacterial load is significantly associated with worse outcomes [110]. Our study reported the first probability histogram that ended with various patterns under a range of initial *Salmonella* loads. The probability histograms describe the relationship between probability of ending with each HIR pattern and *Salmonella* challenge levels. Moreover, we observed that HIR could end in various outcomes (healing response *vs*. persistent infection *vs*. hyperinflammatory response *vs*. organ dysfunction) with the same initial loads of *Salmonella*. These observations could explain the reason why effectiveness of therapeutic intervention is different from patient to patient.

Our results suggest that levels of TNF-α, HMGB-1, CRP, and IL-10: TNF-α ratios, and CD4+ T cell: CD8+ T cell ratios could be recognized as biomarkers for HIR because these indicators and/or ratios have distinct patterns between a healthy state (i.e. a healing response) and hypothetical death state (i.e. hyperinflammatory response or organ dysfunction). Specifically, our simulation results showed that averaged IL-10: TNF-α ratios were significantly elevated in the healing response from 8 to 14 hours compared to the ratios in the hyperinflammatory response and during organ dysfunction (*P* = 0.0061 and *P* = 0.0152, respectively, using the Mann-Whitney *U* tests, as shown in S1 Fig). These data suggest that IL-10: TNF-α ratios could be recognized as a predictive marker for HIR at an early stage because HIR would lead to a more likely death state if a lower ratio of IL-10: TNF-α is observed during the first 14 hrs of infection. Although the averaged IL-10: TNF-α ratios were elevated in both the hyperinflammatory response and in organ dysfunction compared to those in a healing response, IL-10: TNF-α ratios were observed to approach similar values seen during a healing response after the first 14 hrs of infection in some replications. A possible reason for this is that because TNF-α level dropped at a late stage of HIR, and IL-10 levels were increasing due to hyperinflammatory responses. It appears that severe hepatocyte damage was mostly caused by a persistent elevation of inflammatory cytokines such as *HMGB-1*(Figs 8K and 10K). This would indicate that our model is beginning to accurately reflect biological situations since this parallels the *in vivo* experience [113, 127, 128, 142] where a persistent elevation of *HMGB-1* in patients with severe sepsis and mice with organ damage have high HMGB-1 concentrations. Therefore, HMGB-1 can be another predictive marker for HIR progression at a late stage. To conclude, our simulated results suggest that clinicians should use a combination of predictive markers at different stages (IL-10: TNF-α ratios at an early stage *vs*. HMGB-1 at a late stage). This approach could be more accurate to predict the progression of HIR.

Strategies for sepsis treatment have been discussed extensively in recent years [10, 134–136, 143–146], but no general agreement exists regarding efficacy of these strategies. This lack of consensus is due to the complex nature of what causes sepsis to progress, including different clinical and experimental settings, and heterogeneous groups of patients with infections caused by various microorganisms [147]. Our simulated results suggest that the anti-agent treatments are effective within a constrained time window (Fig 17). These findings support contentions that timing of therapy is critical to success [11, 148, 149]. As initial loads of *Salmonella* increase, the probability of a hyperinflammtory response or organ dysfunction increases (Figs 12 and 13). Therefore, based on the modelling we predict that a higher dose of anti-agent treatment might be necessary during a more severe infection. Moreover, individuals with HIR should be classified into multiple groups based on the extent of infection. Therefore, different amounts of anti-agents should be administered to different groups of patients at multiple treatment time points. Given the consistent outcome between our simulations and existing studies [10, 134–137], it suggests that our IMMABM is beginning to accurately reflect some aspects of HIR. IMMABM can provide an initial *silico* test for proposed therapeutic agents. The addition/inclusion of different immune components can also be done to provide insights about how outcomes can change. We saw distinct changes in the outcome of HIR when we added acquired immune components. Although experimental studies have shown that sepsis in humans is not a model of veterinary sepsis and implementation of an animal sepsis model to human medicine must be further validated [148], IMMABM modeling techniques could be applied to human medicine and the IMMABM can be refined as human data becomes available in the future. As the IMMABM is constructed now and by future refinements, this tool will allow us to explore various types of treatments to evaluate their possible effectiveness and could help in the design of future preclinical experiments.

### 4.2 Model simplification and generalizations

Previously, ABM has been employed to describe complex and nonlinear biological immune processes responding to infection [41]. Compared to traditional differential equation models, ABM is more similar to the description and representation of a true biological system because it can incorporate stochastic and spatial processes of cell interactions in a host-pathogen system. The IMMABM in this study simulated dynamic patterns of essential variables and captured quantitative changes in dynamic patterns of an HIR using various doses of *Salmonella* “infection”. Furthermore, the IMMABM allowed us to simulate the distribution of changes, reflected as dynamic patterns, and provided insights into the probability of those changes. The “therapy” experiment demonstrated that this ABM could begin to provide insights about possible treatments prior clinical trials, which could save time and resources by eliminated ineffective therapies from lab experiments and more importantly, clinical trials.

Although ABMs are advantageous compared to mathematical models [15], they are limited as an “instructive” tool and still cannot represent real immune responses in disease progression because they fail in one-to-one mapping of components and processes to biological systems. Since every intermediate biological processes of HIR cannot be simultaneously incorporated into the IMMABM, reasonable assumptions and simplifications of biological responses must be made when building an ABM. For example, in our IMMABM, we did not model that *Salmonella* replicates within neutrophils even though we know that they are a primary replication site *in vivo* [31]. Therefore, this type of *Salmonella* replication could be considered for inclusion in a future model. ABM assumptions can also be more complicated than just leaving out some biological responses because there are conflicting data or the biology is very complex.

We modeled that *TNF-α* induced apoptosis of hepatocytes because *TNF-α* secretion from activated *Kupffer Cells* induces apoptosis in hepatocytes [32]. However, another study showed hepatocyte apoptosis was induced by *TNF-α* only in combination with the transcriptional inhibitor actinomycin D (ActD) [150]. Because our model considered a general concept of HIR at the current stage, we modeled that *TNF-α* would induce hepatocyte apoptosis if *TNF-α* were bound to hepatocytes.

Mast cells release many biologically active molecules and chemical substances, such as protease and IL-6, which decrease or increase survival rates of septic patients [151–153]. *Salmonella* that bind to mast cells eventually die because of the substances secreted by mast cells. Therefore, in order to simplify our ABM, we considered only some main functions of mast cells during inflammation. For many years, mast cells were believed to phagocytize *Salmonella* [154]. However, a recent experiment showed [155] that mast cells bind to *Salmonella*, making them unable to phagocytize *Salmonella*. Therefore, we did not model that mast cells phagocytize *Salmonella*. Instead, we modeled that *Salmonella* binds to mast cells which initiates the release of *TNF-α* from the engaged mast cells.

T cell subpopulations have been reported to express *IL-10* under various conditions [156], making it difficult to estimate *IL-10* production. Because *IL-10* levels released from T cells varies due to the type or concentration of stimuli, we modeled that CD4^+^ T cells produce *IL-10* and we estimated the average release rate of *IL-10*. We did not differentiate helper T cells to specific types (*e*.*g*. Th2 or Treg) identified in biological process.

Plasma cells secrete antibody [79], but we did not incorporate this specific B cell population into our ABM. We modeled that B cells released antibody knowing that this does not mimic the real biological system. Likewise, when antibody is released from plasmas cells, T_H_ cells define the isotype of the antibody [79]. However, we did not model specific antibody isotypes in our model. Furthermore, we ignored the fact that antibody opsonization also induces stimulation of the release of various cytokines and the type of FcR engagement can alter cell function [157, 158]. We also did not incorporate that antibody-opsonized *Salmonella* are phagocytized better by neutrophils and macrophages compared to *Salmonella* alone [79].

We did not model natural killer cells in our ABM and we ignored effects of other pro-inflammatory cytokines such as *IL-1*, *IL-12*, and *IL-8*. Also, biological immune responses to infection are recognized as a series of complex processes including intracellular signal transductions (including activation of gene transcription) and intercellular interactions between cells. These biological processes can be developed over time and will evolved as our understanding of these processes becomes more sophisticated [41]. Therefore, our IMMABM is still under development and has the potential to incorporate many of the variables that we have left out at the present time.

One final important consideration is that an ABM requires a high level of computational effort in order to simulate the detailed interactions between classes of agents in the HIR. This is particularly true because the ABM is designed to describe the aggregated level of components by simulating individual agent behavior and interactions. These processes are occurring in parallel and require extensive computational effort and high computational efficiency [41]. An average of 18 minutes was required to run 300 simulation steps in one replication of IMMABM for a healing and persistent infection response in the IMMABM. The computational times were significantly more for scenarios with hyperinflammatory or organ dysfunction responses. In those cases, computational efficiency required an average of 10 minutes per simulation step (an average of 50 hours if the run has 300 simulation steps, or a total 208.3 days (approximation) for 100 replications). Therefore, one of the factors that limits the number of variables included in an ABM is the computer power available. This limitation directs us to improve computer power by implementing an object-oriented programming and parallel computing in the future.

## 5. Future Research

Limitations of current ABMs provide opportunities for future enhancements. A major step forward will include the addition of one or more of the sophisticated cellular and molecular pathways discussed in Section 4.

The activation of the coagulation cascade is characteristically seen in patients with sepsis [159]. Activated protein C (APC), as an endogenous protein with the ability to modulate coagulation, has currently been approved to be the only pharmacologic therapy in the treatment of severe sepsis [159, 160], highlighting the importance of coagulation and fibrinolysis in sepsis [160]. Thus, modeling complement cascades of inflammatory responses and possible progression to coagulation episodes during sepsis would also help the understanding of both inflammation and coagulation and associated therapeutic targets during sepsis progression. An explicit modeling of coagulation cascades needs to incorporate to IMMABM in order to describe hemostasis during sepsis.

Mediator-directed treatments could be incorporated into this IMMABM in order to implement pre-clinical treatment tests *in silico*. Initial *silico* simulation of IMMABM allowed us to recognize that a combination of anti-TNF-α and anti-HMGB-1 agents could significantly improve survival rates in HIR. Furthermore, we also observed that the time drugs were administered also impacts HIR outcomes. This not only provides evidence that the core IMMABM is sound, it also provides hope that it can be developed into an effective tool to assist in physicians in their clinical decision-making process.

Current ABMs also require computational resources. For the current 401 × 401 2D grid simulation size, the average simulation implementation time ranged from 18 mins to 50 hrs per replication. Computational time exponentially increased as the number of interactions among agents increased because of the numerous repetitive interactions. Therefore, another future direction of ABM research could be to reduce this computational hurdle by designing new and efficient computational algorithms.

## Supporting Information

S1 Fig Comparison of IL-10: TNF-α ratio among healing response, hyperinflammatory response, and organ dysfunction responses vs. the first 14 hrs of infection time. Mean values of IL-10: TNF-α ratios were measured at each simulation time point (replications = 10).
(TIF)

S2 Fig Probabilities of leading to heading response, persistent infection, hyperinflammatory response, and organ dysfunction in the presence of Acquired Immunity. *Salmonella* initial loads range from 200 to 3200 counts.
(TIF)

S1 Table Agent Behaviors and Agent Update Rules in IMMABM. (DOCX)

S2 Table Agent Types and Agent Behaviors in IMMABM Based on Biological Behaviors (Agent types in “Agent Behavior(s)” are highlighted in Italic format, except terminology Salmonella enterica serovar Typhimurium (Salmonella) is Italic format in both “Biological Behavior(s)” and “Agent Behavior(s)”). (DOCX)

S3 Table Experimental data and value of system parameters in IMMABM. (DOCX)
