# Supplementary figures and images for: An Agent-Based Model of a Hepatic Inflammatory Response to Salmonella: A Computational Study under a Large Set of Experimental Data
Source: PLoS One. 2016 Aug 24;11(8):e0161131. doi: 10.1371/journal.pone.0161131 (PMC4996536; doi:10.1371/journal.pone.0161131)

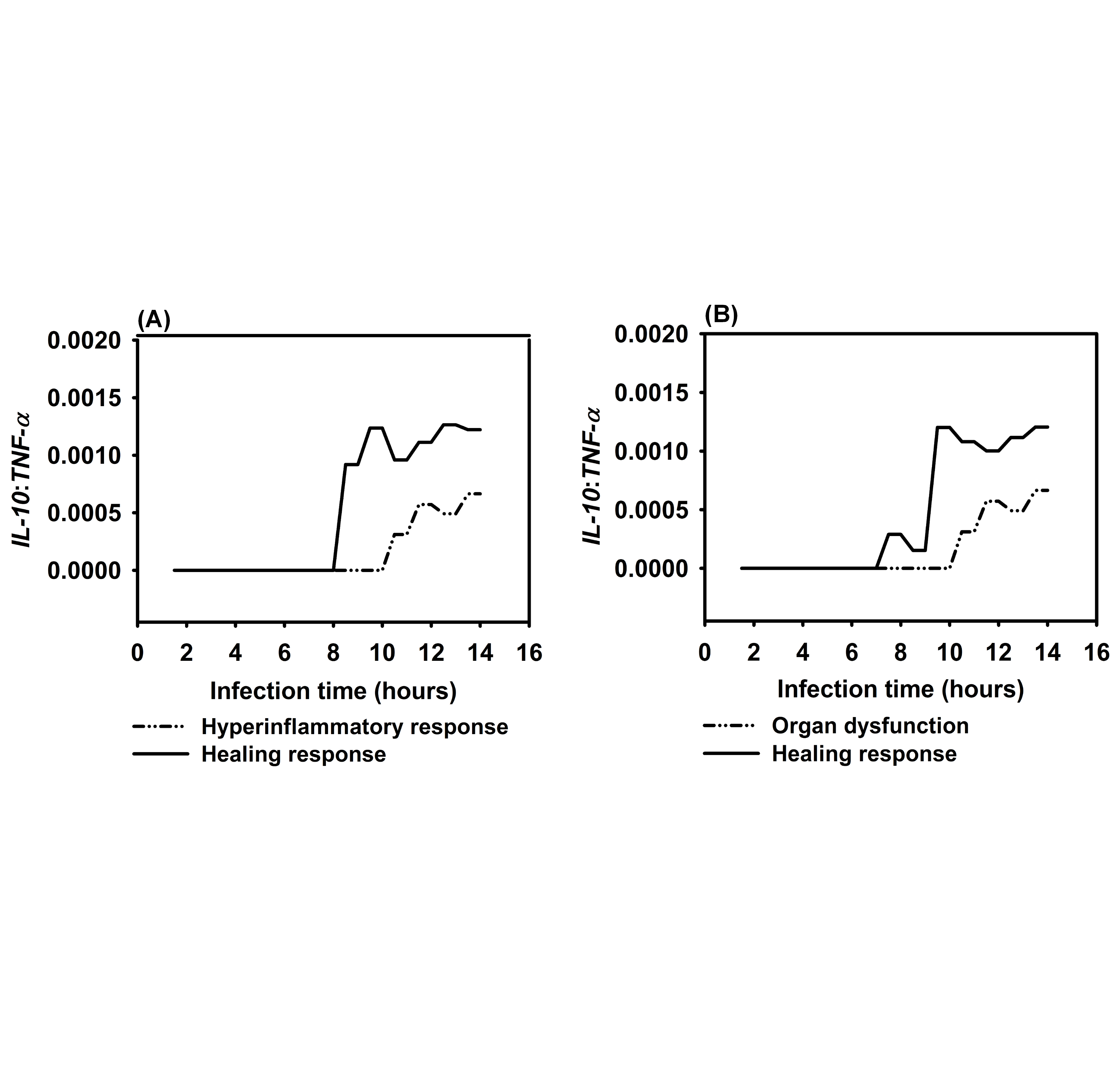

Supplement: S1 Fig — Mean values of IL-10: TNF-α ratios were measured at each simulation time point (replications = 10). (TIF) [file pone.0161131.s001.tif]

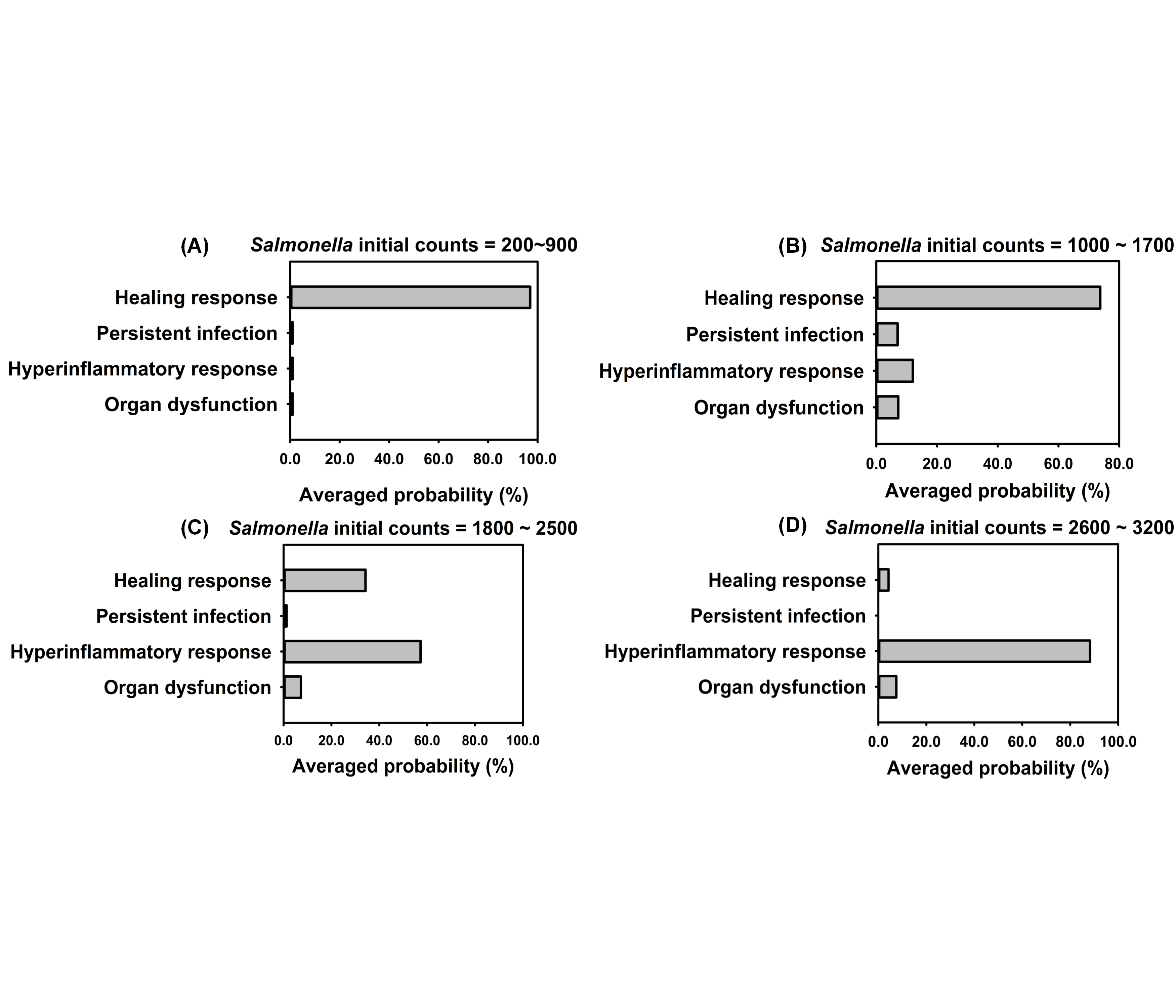

Supplement: S2 Fig — Salmonella initial loads range from 200 to 3200 counts. (TIF) [file pone.0161131.s002.tif]
