# Supplementary material for: An Agent-Based Model of a Hepatic Inflammatory Response to Salmonella: A Computational Study under a Large Set of Experimental Data
Source: PLoS One. 2016 Aug 24;11(8):e0161131. doi: 10.1371/journal.pone.0161131 (PMC4996536; doi:10.1371/journal.pone.0161131)
Supplement: S1 Table — (DOCX) [file pone.0161131.s003.docx]

Table S1. Agent Behaviors and Agent Update Rules in IMMABM

Note: Data in this table are from Table S3.

| WORLD  The world is a 401 units x 401 units square. |
| --- |
| Initialization (The initialization is setup under normal conditions)   1. Hepatocytes are created. The initial number of hepatocytes is 80,200. 2. Sinusoidal endothelial cells (SECs) are created and parallel arrangement in a two-dimensional world. They are structural agents which model the boundary of the liver sinusoid. The initial number of SECs is 26,466, which is approximately 1/3 of hepatocyte population. 3. The liver sinusoid is created. They are modeled as “patches” with boundary lined by SECs. Liver sinusoid is separated from the hepatocytes by the space of Disse. 4. The space of Disse is created as “patches” to model locations between hepatocytes and the liver sinusoid. 5. Kupffer Cells are created adherent to SECs in the liver sinusoid. The initial number of Kupffer Cells is 20,160, which is approximately 1/4 of the hepatocyte population. Kupffer Cells are in a parallel arrangement in a two-dimensional world. 6. Mast cells are placed in the space of Disse. The initial number of mast cells is 10,426, which is approximately 1/8 of the hepatocyte population. Mast cells are in a parallel arrangement in a two-dimensional world. 7. Circulating neutrophils are created in the liver sinusoid, which take the place of being normally formed in the bone marrow. The initial number of circulating neutrophils is 1000. Circulating neutrophils are circle shaped with “multi-lobed nuclei” inside. 8. Circulating monocytes are created in the liver sinusoid, which take the place of being normally formed in the bone marrow. The initial number of circulating monocytes is 1000. Circulating monocytes are by circle shaped with “circle-shaped nuclei” inside. 9. The portal triad is represented as “patches” to model the site of T cells and B cell generation. 10. Circulating CD4^+^ T cells are created in portal triad. The initial number of circulating CD4^+^ T cells is 739, which is approximately 14% of total lymphocytes in the lymph node. CD4^+^ T cells are represented as yellow circles in the portal triad. 11. Circulating CD8^+^ T cells are created in portal triad. The initial number of circulating CD8^+^ T cells is 482, which is approximately 9% of total lymphocytes in the lymph node. CD8^+^ T cells are represented as green circles in portal triad. 12. Circulating B cells are created in portal triad. The initial number of circulating B cells is 3,235, which is approximately 60% of total lymphocytes in the lymph node. B cells are represented as blue circles in the portal triad. |
| ABM RULES (1 TICK REPRESENTS 1 HR DURING SIMULATION)  AGENTS  Kupffer Cell. (Called *KupfferCell* in the ABM.)   1. Phagocytose 90 ~ 95% *Salmonella* over 6 ticks. 2. 5% ~ 10% of the Kupffer Cells are killed by *Salmonella* from 4 tick - 6 ticks. [Active] Kupffer Cells are killed by *Salmonella*. [Inactive] Kupffer Cells are killed by *Salmonella* if interact with CD4^+^ T cells. 3. Phagocytose hepatocyte debris. 4. Phagocytose activated circulating neutrophils in the liver sinusoid. 5% of the activated circulating neutrophils are phagocytosed by Kupffer Cells per tick. Activated circulating neutrophils are defined as circulating neutrophils that are attracted to SECs. 5. [Inactive] Secretes TNF-α. Activated if it interacts with *Salmonella*, hepatocyte debris, or activated circulating neutrophils. Rate of TNF-α released from Kupffer cells is 2.09×10^-5^ ~ 2.30×10^-4^ pg/Kupffer Cell/tick. TNF production is computed with Michaelis-Menten Kinetics:   TNF-α[new] = ((maximum production rate * Kupffer Cell [who release TNF-α])/(Kupffer Cell count at which the reaction rate is half of maximum production rate + Kupffer Cell [who release TNF-α])) × Kupffer Cell [who release TNF-α]. Maximum production rate is equivalent to 2.30×10^-4^ pg/Kupffer Cell/tick.   1. [With 14.3% probability] Bind to IL-10. [Inactive] Produce TNF-α. 2. Kupffer Cells are replenished at a rate of 0.63% ~ 0.79% of monocyte-derived-macrophage type I or monocyte-derived-macrophage type II per tick. 3. [Inactive] Secretes IL-10. Activated if they ingest apoptotic hepatocytes. Rate of IL-10 released from Kupffer Cells is 4.98×10^-6^ pg/Kupffer Cell/tick. IL-10 production is computed with Michaelis-Menten Kinetics:   IL-10 [new] = ((maximum production rate * Kupffer Cell [who release IL-10])/(Kupffer Cell count at which the reaction rate is half of maximum production rate + Kupffer Cell [who release IL-10])) × Kupffer Cell [who release IL-10]. Maximum production rate is equivalent to 4.98×10^-6^ pg/Kupffer Cell/tick.   1. [With 16.7% probability] Apoptotic Kupffer cells interact with CRP. 2. [With 20% probability ] CRP-opsonized apoptotic Kupffer cells are phagocytized by Kupffer cells, or mast cells, or monocyte-derived-macrophage type I, or monocyte-derived-macrophage type II, or neutrophils.    1. If CRP-opsonized apoptotic Kupffer Cells are phagocytized by Kupffer Cells, [Inactive] secretes TNF-α. [Increase] phagocytic rate.    2. If CRP-opsonized apoptotic Kupffer Cells are phagocytized by monocyte-derived-macrophage type I, [Active] secretes TNF-α. [Increase] phagocytic rate.    3. If CRP-opsonized apoptotic Kupffer Cells are phagcytized by neutrophils, [Inactive] neutrophil adhere to SECs. 3. CRP-opsonized apoptotic Kupffer Cells die if the number of ticks is higher than 1 tick, the lifespan of apoptotic cells. Reset states of interacted agents. 4. [With 20% probability] Kupffer Cells phagocytize CRP-opsonized *Salmonella*, CRP-opsonized apoptotic mast cells, CRP-opsonized apoptotic monocyte-derived-macrophage type I, CRP-opsonized apoptotic monocyte-derived-macrophage type II, CRP-opsonized apoptotic neutrophils, or CRP-opsonized apoptotic hepatocytes. [Inactive] secretes TNF-α. [Increase] phagocytic rate. 5. [Inactive] Kupffer Cells which phagocytose *Salmonella* undergo apoptosis. [Active] If they interact with CD8^+^ T cells. Kupffer Cells die if the number of ticks is higher than 4.   *Salmonella*. (Called *Salmonella* in the ABM.)   1. [Inactive] 90 ~ 95% *Salmonella* are phagocytized by Kupffer Cells over 6 ticks. [Active] if they interact with Kupffer Cells. 2. Phagocytosed *Salmonella* are killed by Kupffer Cells if the total number of ticks is higher than the lifespan of *Salmonella* in Kupffer Cells. The lifespan of *Salmonella* in Kupffer Cells is generated from a random-generator with a uniform distribution from 0 to 6 ticks. 3. *Salmonella* survive and replicate within apoptotic Kupffer Cells. The maximum number of *Salmonella* in one Kupffer Cell is 50. The replication rate is 0.42 ~ 1.04/tick. *Salmonella* population is computed with the logistic equation:   *Salmonella* [new] =replication rate * *Salmonella* [old] *(1-*Salmonella* [old]/*Salmonella* carrying capacity in Kupffer Cells). *Salmonella* carrying capacity in Kupffer Cells = 4558000.   1. Apoptotic Kupffer Cells die and release *Salmonella*. Released *Salmonella* interact with SECs, hepatocytes, NETs, antibody, CRP or normal inflammatory cells (including Kupffer Cells, mast cells, neutrophils, monocyte-derived-macrophage type I). The chance of interaction is dependent on the random movement of the *Salmonella* agent. 2. [Inactive] Released *Salmonella* infect SECs. [Active] if they interact with SECs. The replication rate is 0.32/tick. The maximum number of *Salmonella* in one SEC is 3. The *Salmonella* population is computed with a logistic equation:   *Salmonella* [new] =replication rate * *Salmonella* [old] *(1-*Salmonella* [old]/*Salmonella* carrying capacity in SECs). *Salmonella* carrying capacity in SECs=67000.   1. [Inactive] Released *Salmonella* from killed (dead) SECs move to mast cells, hepatocytes or interact with nearby inflammatory cells (including neutrophils and MDMIs). [Active] if SECs die. Infected SECs die after 1 tick. 2. [Inactive] Released *Salmonella* infect and replicate within healthy hepatocytes. [Active] if released *Salmonella* interact with hepatocytes. Replication rate is 0.05 ~ 0.26/tick. State variable named “hepatocyteInteractWith*Salmonella*” is updated from 0 to 1. The maximum number of *Salmonella* in one hepatocyte is 3. *Salmonella* population is computed with a logistic equation:   *Salmonella* [new] =replication rate * *Salmonella* [old] *(1-*Salmonella* [old]/*Salmonella* carrying capacity in hepatocytes). *Salmonella* carrying capacity in hepatocytes=817000.   1. *Salmonella* are released from apoptotic hepatocytes and infect nearest hepatocytes. The chance of interaction is dependent on the random movement of the *Salmonella* agent. 2. [Inactive] Released *Salmonella* are phagocytosed by neutrophils. [Active] if interact with neutrophils. Phagocytic rate of *Salmonella* by neutrophils is 2.94 ~ 12.94 *Salmonella*/neutrophil/tick. The maximum number of *Salmonella* interactions with an activated neutrophil is 17. 3. Phagocytized *Salmonella* are killed by neutrophils if the total number of ticks is higher than 2 ticks. 4. [Inactive] Released *Salmonella* are phagocytosed by monocyte-derived-macrophage type I. [Active] if interact with monocyte-derived-macrophage type I. Phagocytic rate of *Salmonella* by monocyte-derived-macrophage type I is 1.18 ~ 6.74 *Salmonella*/macrophage/tick. 5. Phagocytosed *Salmonella* are killed by monocyte-derived-macrophage type I if the total number of ticks is higher than 2 ticks. 6. *Salmonella* survive and replicate within apoptotic monocyte-derived-macrophage type I. The replication rate is 0.9 ~ 10.9/tick. The *Salmonella* population is computed with a logistic equation:   *Salmonella* [new] =replication rate * *Salmonella* [old] *(1-*Salmonella* [old]/*Salmonella* carrying capacity in macrophages). *Salmonella* carrying capacity in macrophages =4558000.   1. [Inactive] Released *Salmonella* are killed by neutrophil extracellular traps (NETs). [Active] if they interact with NETs. The NETs is a complex of Myeloperoxidase (MPO) and neutrophil elastase (NE). 22.79% of *Salmonella* are trapped and killed by 1µM NETs per tick. 2. [Inactive] Released *Salmonella* bind to mast cells. [Active] if they interact with mast cells. The chance of an interaction is determined by an estimated probability. 3. [With 20% probability] CRP-opsonized *Salmonella* are phagocytosed by phagocytic cells (Kupffer Cells, or mast cells, or neutrophils, or monocyte-derived-macrophage type I, or monocyte-derived-macrophage-type II). [Update] phagocytosis rate. 4. CRP-opsonized *Salmonella* die if the number of ticks is higher than 1 tick, the lifespan of *Salmonella*. Reset states of interacted agents. 5. [With 20% probability] Antibody-opsonized *Salmonella* are phagocytosed by phagocytic cells (Kupffer Cells, or mast cells, or neutrophils, or monocyte-derived-macrophage type I, or monocyte-derived-macrophage type II). [Update] phagocytosis rate. 6. [With 20% probability] Antibody-opsonized *Salmonella* die if the number of ticks is higher than 1 tick, the lifespan of *Salmonella*. Reset states of interacted agents. 7. [Inactive] *Salmonella* undergo apoptosis by other factors. [Active] if they bind to mast cells. Apoptosis rate is estimated.   Hepatocyte. (Called *Hepatocyte* in the ABM.)   1. [Inactive] Hepatocytes are infected by *Salmonella* and become apoptotic. [Active] if they interact with *Salmonella*. The chance of interaction is dependent on the random movement of *Salmonella*. 2. [Inactive] Hepatocytes become apoptotic by interacting with TNF-α. [Active] if they interact with TNF-α. The interaction between TNF-α and hepatocytes is dependent on the random diffusion of TNF-α. 3. [Inactive] Produce CRP. [Active] if monocyte infiltration is detected. The rate is 0.2pg/hepatocyte/tick. CRP production is computed with Michaelis-Menten Kinetics:   CRP [new] = ((maximum production rate * Hepatocyte [old])/(Hepatocyte count at which the reaction rate is half of maximum production rate +Hepatocyte [old])) × Hepatocyte [old]. Maximum production rate is equivalent to 0.2pg/hepatocyte/tick.   1. [Inactive] Apoptotic hepatocytes are cleaned up by neutrophils. [Active] if they interact with neutrophils. The phagocytosis rate of hepatocytes by neutrophils is 0.05 ~ 0.2 hepatocytes/neutrophil/tick. 2. Apoptotic hepatocytes become hepatocyte debris if the total number of ticks is higher than 2 or 3 ticks (2 or 3 is randomly assigned by a random generator). 3. Apoptotic hepatocytes release HMGB-1. The rate is 6.25×10^-5^pg/hepatocyte/tick. HMGB-1 production is computed with Michaelis-Menten Kinetics:   HMGB-1[new] = ((maximum production rate * Hepatocyte [apoptotic])/(Hepatocyte count at which the reaction rate is half of maximum production rate +Hepatocyte [apoptotic])) × Hepatocyte [apoptotic]. Maximum production rate is equivalent to 6.25×10^-5^ pg/hepatocyte/tick.   1. Apoptotic hepatocytes release TNF-α. The rate is 7.14×10^-5^ -9.18×10^-5^ pg/apoptotic hepatocyte/tick. TNF-α production is computed with Michaelis-Menten Kinetics:   TNF-α[new] = ((maximum production rate * Hepatocyte [apoptotic])/(Hepatocyte count at which the reaction rate is half of maximum production rate +Hepatocyte [apoptotic])) × Hepatocyte [apoptotic]. Maximum production rate is equivalent to 9.18×10^-5^ pg/apoptotic hepatocyte/tick.   1. Hepatocytes regenerate. The replication rate is 1.32×10^-3^ ~ 6.80×10^-3^ /tick. The hepatocyte population is computed with a logistic equation:   Hepatocyte [new] =replication rate * Hepatocyte [old] *(1-Hepatocyte [old]/hepatocyte carrying capacity). Hepatocyte carrying capacity = the initial number of hepatocytes  Hepatocyte debris. (Called *HepatocyteDebris* in the ABM.)   - 1. Hepatocyte debris is phagocytosed by phagocytic cells including Kupffer Cells, mast cells, neutrophils, monocyte-derived-macrophage type I, and monocyte-derived-macrophage type II. The phagocytic rate is dependent on random dispersion of hepatocyte debris. The radius of random dispersion is patch-size.   2. [With 20% probability] CRP interacts with hepatocyte debris. CRP-opsonized hepatocyte debris is phagocytosed by phagocytic cells including Kupffer Cells, mast cells, neutrophils, monocyte-derived-macrophage type I, and monocyte-derived-macrophage type II. The phagocytic rate is dependent on random movement of phagocytic cells.   3. Hepatocyte debris degrades naturally. The degredation rate of is estimated.   Resting neutrophil. (Called *RestingNeutrophil* in the ABM.)   1. Resting neutrophil migrate to the liver sinusoid from bone marrow upon infection. The influx rate to the liver sinusoid is 0.125 ~ 14/tick. The resting neutrophil population is computed with a logistic equation:   Resting neutrophil [new] =influx rate * Resting neutrophil [old] *(1-Resting neutrophil [old]/ resting neutrophil carrying capacity). Resting neutrophil carrying capacity = 6.9×10^3^.   1. [Inactive] Resting neutrophils get signals and move to SECs. [Active] if *Salmonella*, TNF-α, and HMGB-1 are detected after 2 ticks. [Set breed] Activated neutrophils. Activation rate is 0.09~0.46/hrs. Activated neutrophil population is computed with law of mass-action:   Activated neutrophil [new] =activation rate * Resting neutrophil population * (*Salmonella* population + TNF-α+ HMGB-1) / Total population. Total population = hepatocyteInitialNumber + mastCellInitialNumber + kupfferCellInitialNumber + SECsInitialNumber.   1. Resting neutrophils undergo aging and apoptosis. The rate of apoptosis is 0.05 ~ 0.092/tick.   Activated neutrophil. (Called *ActivatedNeutrophil* in the ABM.)   1. Activated neutrophils move towards the site of Kupffer Cells, or apoptotic hepatocytes or *Salmonella*. 2. Activated neutrophils undergo aging. State variable named “activatedNeutrophilBecomeApoptotic” is updated from 0 to 1 if apoptosis starts. The rate of apoptosis is 0.098/tick. 3. Apoptotic neutrophils interact with CRP. 4. [With 20% probability] CRP-opsonized apoptotic activated neutrophils are phagocytized by Kupffer Cells, or mast cells, or monocyte-derived-macrophage type I, or monocyte-derived-macrophage type II, or neutrophils.    1. If CRP-opsonized apoptotic activated neutrophils are phagocytosed by Kupffer Cells, [Inactive] secretes TNF-α. [Increase] phagocytic rate.    2. If CRP-opsonized apoptotic activated neutrophils are phagocytosed by monocyte-derived-macrophage type I, [Active] secretes TNF-α. [Increase] phagocytic rate.    3. If CRP-opsonized apoptotic activated neutrophils are phagcytosed by neutrophils, [Inactive] neutrophil adhere to SECs. 5. CRP-opsonized apoptotic activated neutrophils die if the number of ticks is higher than 1 tick, the lifespan of apoptotic activated neutrophils. Reset states of interacted agents. 6. [Inactive] Apoptotic neutrophils are ingested by monocyte-derived-macrophage type II. [Active] if neutrophils interact with monocyte-derived-macrophage type II. Apoptotic neutrophils die after 1 tick. 7. [Inactive] Activated neutrophils produce TNF-α. [Active] if they interact with *Salmonella*. The rate is 0.19 ~ 2.00 pg/neutrophil/tick.   TNF-α production is computed with Michaelis-Menten Kinetics:  TNF-α[new] = ((maximum production rate * Activated neutrophil [interact with *Salmonella*])/(Activated neutrophil count at which the reaction rate is half of maximum production rate +Activated neutrophil [interact with *Salmonella*])) × Activated neutrophil [interact with *Salmonella*]. Maximum production rate is equivalent to 2.00 pg/neutrophil/tick.   1. [Inactive] Activated neutrophils produce IL-10. [Active] if interact with *Salmonella*. The rate is 8.44×10^-5^ ~ 1.03×10^-4^ pg/neutrophil/tick. IL-10 production is computed with Michaelis-Menten Kinetics:   IL-10 [new] = ((maximum production rate * Activated neutrophil [interact with *Salmonella*])/(Activated neutrophil count at which the reaction rate is half of maximum production rate +Activated neutrophil [interact with *Salmonella*])) × Activated neutrophil [interact with *Salmonella*]. Maximum production rate is equivalent to 1.03×10^-4^ pg/neutrophil/tick.   1. [Inactive] Activated neutrophils release MPO, NE, and NETs by degranulation. [Active] if interact with *Salmonella*. New values are computed with the equation: NETs [new] = MPO [new] =NE [new] (NETs is a complex agent of MPO and NE) 2. [With 20% probability] Bind to IL-10. [Inactive] Produce TNF-α or IL-10. 3. [Inactive] Activated neutrophils that phagocytose *Salmonella* undergo apoptosis. [Active] If neutrophils interact with CD8^+^ T cells. Activated neutrophils die if the number of ticks is higher than 4 ticks.   Resting monocyte. (Called *RestingMonocyte* in the ABM.)   1. [Inactive] Resting monocytes migrate to the liver sinusoid from bone marrow upon infection. [Active] After 2 ticks. Influx rate to the liver sinusoid is 1 ~ 1.75/tick. The resting monocyte population is computed with a logistic equation:   Resting monocyte [new] =influx rate * Resting monocyte [old] *(1-Resting monocyte [old]/ resting monocyte carrying capacity). Resting monocyte carrying capacity = 1.4×10^3^.   1. [Inactive] Resting monocytes get a signal and move to SECs. [Active] if *Salmonella*, TNF-α, HMGB-1, and apoptotic neutrophils are detected. [Set breed] monocyte-derived-macrophage type I if interact with *Salmonella*. [Set breed] monocyte-derived-macrophage type II if interact with apoptotic neutrophils. Activation rate is 0.25~4.82/hrs. New population is computed with law of mass-action:   (Monocyte-derived-macrophage type I [new] + monocyte-derived-macrophage type II [New])=activation rate * Resting monocyte population * (*Salmonella* population + TNF-α + HMGB-1 + apoptotic neutrophils) / Total population. Total population = hepatocyteInitialNumber + mastCellInitialNumber + kupfferCellInitialNumber + SECsInitialNumber.   1. Resting monocytes age and undergo apoptosis. The rate of apoptosis is 6.90×10^-3^ ~ 2.10×10^-2^/tick.   Monocyte-derived-macrophage type I. (Called *MDMI* in the ABM.)   1. Phagocytose *Salmonella* [See *Salmonella* 11]. 2. [Inactive] Monocyte-derived-macrophage type I produce TNF-α. [Active] if they phagocytose *Salmonella*. The rate is 1.70×10^-4^ pg/macrophage/tick. TNF-α production is computed with Michaelis-Menten Kinetics:   TNF-α [new] = ((maximum production rate * Monocyte-derived-macrophage type I [interact with *Salmonella*])/(Monocyte-derived-macrophage type I count at which the reaction rate is half of maximum production rate + Monocyte-derived-macrophage type I [interact with *Salmonella*])) × Monocyte-derived-macrophage type I [interact with *Salmonella*]. Maximum production rate is equivalent to 1.70×10^-4^ pg/macrophage/tick.   1. [Inactive] Produce IL-10. [Active] if they phagocytose apoptotic T cells. The rate is 2.02×10^-5^pg/monocyte-derived-macrophage type I/tick. IL-10 production is computed with Michaelis-Menten Kinetics:   IL-10 [new] = ((maximum production rate * Monocyte-derived-macrophage type I [interact with apoptotic T cells])/(Monocyte-derived-macrophage type I count at which the reaction rate is half of maximum production rate + Monocyte-derived-macrophage type I [interact with apoptotic T cells])) × Monocyte-derived-macrophage type I [interact with apoptotic T cells]. Maximum production rate is equivalent to is 2.02×10^-5^pg/monocyte-derived-macrophage type I/tick.   1. Monocyte-derived-macrophage type I are killed by *Salmonella*. The killing rate is estimated. [Active] Monocyte-derived-macrophage type I are killed by *Salmonella*. [Inactive] Monocyte-derived-macrophage type I are killed by *Salmonella* if they interact with CD4^+^ T cells. 2. Monocyte-derived-macrophage type I undergo aging. The rate needs to be estimated. State variable named “MDMIBecomeApoptotic” is updated from 0 to 1. 3. [With 20% probability ] CRP-opsonized apoptotic monocyte-derived-macrophage type I are phagocytosed by Kupffer Cells, or mast cells, or monocyte-derived-macrophage type I, or monocyte-derived-macrophage type II, or neutrophils.    1. If CRP-opsonized apoptotic monocyte-derived-macrophage type I are phagocytosed by Kupffer Cells, [Inactive] they secrete TNF-α. [Increase] phagocytic rate.    2. If CRP-opsonized apoptotic monocyte-derived-macrophage type I are phagocytosed by monocyte-derived-macrophage type I, [Active] secretes TNF-α. [Increase] phagocytic rate.    3. If CRP-opsonized apoptotic monocyte-derived-macrophage type I are phagcytosed by neutrophils, [Inactive] the neutrophils adhere to SECs. 4. CRP-opsonized apoptotic monocyte-derived-macrophage type I die if the number of ticks is higher than 1 tick, the lifespan of apoptotic monocyte-derived-macrophage type I. Reset states of interacted agents. 5. [With 20% probability] Monocyte-derived-macrophage type I phagocytose CRP-opsonized *Salmonella*, CRP-opsonized apoptotic mast cells, CRP-opsonized apoptotic monocyte-derived-macrophage type I, CRP-opsonized apoptotic monocyte-derived-macrophage type II, CRP-opsonized apoptotic neutrophils, or CRP-opsonized apoptotic hepatocytes. [Active] secretes TNF-α. [Increase] phagocytic rate. 6. 0.63% ~ 0.79% of monocyte-derived-macrophage type I transform to Kupffer Cells every tick. [set breed] Kupffer Cells [See Kupffer Cell 7]. 7. [With 20% probability] Bind to IL-10. [Inactive] Produce TNF-α. 8. Monocyte-derived-macrophage type I activate T cell activation. 9. [Inactive] Monocyte-derived-macrophage type I who phagocytose *Salmonella* undergo apoptosis. [Active] If interact with CD8^+^ T cells. Monocyte-derived-macrophage type I die if the number of ticks is greater than 4 ticks.   Monocyte-derived-monocytes type II. (Called *MDMII* in the ABM.)   1. Phagocytose apoptotic neutrophils [See Activated neutrophil 6]. 2. [Inactive] Produce HMGB-1. [Active] if phagocytosing apoptotic neutrophils. The rate is 9.38×10^-3^ ~ 4.97×10^-1^ pg/monocyte-derived-macrophage type II/tick. HMGB-1 production is computed with Michaelis-Menten Kinetics:   HMGB-1 [new] = ((maximum production rate * Monocyte-derived-macrophage type II [interact with apoptotic neutrophils])/(Monocyte-derived-macrophage type II count at which the reaction rate is half of maximum production rate + Monocyte-derived-macrophage type II [interact with apoptotic neutrophils])) × Monocyte-derived-macrophage type II [interact with apoptotic neutrophils]. Maximum production rate is equivalent to 4.97×10^-1^ pg/monocyte-derived-macrophage type II/tick.   1. [Inactive] Produce IL-10. [Active] if phagocytosing apoptotic neutrophils or phagocytosing apoptotic T cells. The rate is 2.02×10^-5^pg/monocyte-derived-macrophage type II/tick. Assume they produce the same rate of IL-10 under both conditions. IL-10 production is computed with Michaelis-Menten Kinetics:   IL-10 [new] = ((maximum production rate * Monocyte-derived-macrophage type II [interact with apoptotic neutrophils or apoptotic T cells])/(Monocyte-derived-macrophage type II count at which the reaction rate is half of maximum production rate + Monocyte-derived-macrophage type II [interact with apoptotic neutrophils or apoptotic T cells])) × Monocyte-derived-macrophage type II [interact with apoptotic neutrophils or apoptotic T cells]. Maximum production rate is equivalent to is 2.02×10^-5^pg/monocyte-derived-macrophage type II/tick.   1. Phagocytose apoptotic T cells. 2. Undergo natural aging. The rate needs to be estimated. State variable named “MDMIIBecomeApoptotic” is updated from 0 to 1. 3. [With 20% probability] CRP-opsonized apoptotic monocyte-derived-macrophage type II are phagocytosed by Kupffer Cells, or mast cells, or monocyte-derived-macrophage type I, or monocyte-derived-macrophage type II, or neutrophils.    1. If CRP-opsonized apoptotic monocyte-derived-macrophage type II are phagocytosed by Kupffer Cells, [Inactive] secretes TNF-α. [Increase] phagocytic rate.    2. If CRP-opsonized apoptotic monocyte-derived-macrophage type II are phagocytosed by monocyte-derived-macrophage type I, [Active] secretes TNF-α. [Increase] phagocytic rate.    3. If CRP-opsonized apoptotic monocyte-derived-macrophage type II are phagcytosed by neutrophils, [Inactive] the neutrophils adhere to SECs. 4. CRP-opsonized apoptotic monocyte-derived-macrophage type II die if the number of ticks is higher than 1 tick, the lifespan of apoptotic monocyte-derived-macrophage type II. Reset states of interacted agents. 5. [With 20% probability] Monocyte-derived-macrophage type II phagocytose CRP-opsonized *Salmonella*, CRP-opsonized apoptotic mast cells, CRP-opsonized apoptotic monocyte-derived-macrophage type I, CRP-opsonized apoptotic monocyte-derived-macrophage type II, CRP-opsonized apoptotic neutrophils, or CRP-opsonized apoptotic hepatocytes. [Active] secretes TNF-α. [Increase] phagocytic rate. 6. 0.63% ~ 0.79% of monocyte-derived-macrophage type II transform to Kupffer Cells every tick. [set breed] Kupffer Cells [See Kupffer Cell 7]. 7. [With 20% probability] Bind to IL-10. [Inactive] Produce HMGB-1. 8. Monocyte-derived-macrophage type II activate T cell activation.   Mast cell. (Called *MastCell* in the ABM.)   1. Proliferate at a rate of 9.45×10^-4^ ~ 1.37×10^-2^/tick. 2. [Inactive] Produce histamine. [Active] If interact with antibody-opsonized *Salmonella*. The rate is 0.12-0.18 pg /mast cell/tick. Histamine production is computed with Michaelis-Menten Kinetics:   Histamine [new] = ((maximum production rate * Mast cell [interact with antibody-opsonized *Salmonella*])/(Mast cell count at which the reaction rate is half of maximum production rate + Mast cell [interact with antibody-opsonized *Salmonella*])) × Mast cell [interact with antibody-opsonized *Salmonella*]. Maximum production rate is equivalent to 0.18 pg /mast cell/tick.   1. [Inactive] Produce TNF-α. [Active] if bind to *Salmonella* or bind to antibody-opsonized *Salmonella* [See *Salmonella* 15]. The rate is 1.33×10^-7^ ~ 1.52×10^-7^ pg/mast cell/tick. TNF-α production is computed with Michaelis-Menten Kinetics:   TNF-α [new] = ((maximum production rate * Mast cell [bind to *Salmonella*])/(Mast cell count at which the reaction rate is half of maximum production rate + Mast cell [bind to *Salmonella*])) × Mast cell [bind to *Salmonella*]. Maximum production rate is equivalent to is 1.52×10^-7^ pg/mast cell/tick.   1. [Inactive] Recruit T cells to liver sinusoid. [Active] If both histamine and TNF-α are detected. 2. [With 20% probability] Bind to IL-10. [Inactive] Produce TNF-α. 3. [With 20% probability] Mast cells phagocytose CRP-opsonized *Salmonella*, CRP-opsonized apoptotic Kupffer Cells, CRP-opsonized apopototic hepatocyte debris, CRP-opsonized apoptotic neutrophils, CRP-opsonized monocyte-derived-macrophage type I, or CRP-opsonized monocyte-derived-macrophage type II. 4. Undergo natural aging. The apoptotic rate needs to be estimated.   CD4 T cell. (Called *CD4TCell* in the ABM.)   1. [Inactive] Migrate from the portal triad to the liver sinusoid. [Active] if MDMIs or MDMIIs or both histamine and TNF-α are detected. The migration rate is 1.24×10^-3^-2.75×10^-2^/hrs. CD4^+^ T cell population is computed with a logistic equation:   CD4^+^ T cell [new] =influx rate * CD4^+^ T cell [old] *(1-CD4^+^ T cell [old]/ CD4^+^ T cell carrying capacity). CD4^+^ T cell carrying capacity = 1373425.   1. [Inactive] Produce TNF-α. [Active] If interact with APCs including Kupffer Cells, neutrophils, or monocyte-derived-macrophage type I. The rate is 6.94×10^-7^pg/T cell/hrs. TNF-α production is computed with Michaelis-Menten Kinetics:   TNF-α [new] = ((maximum production rate * CD4^+^ T cell [bind to APCs])/( CD4^+^ T cell count at which the reaction rate is half of maximum production rate + CD4^+^ T cell [bind to APCs])) × CD4^+^ T cell [bind to APCs]. Maximum production rate is equivalent to is 6.94×10^-7^ pg/CD4^+^ T cell /tick.   1. [Inactive] Produce IL-10. [Active] If interact with APCs including Kupffer Cells, neutrophils, or monocyte-derived-macrophage type I. The rate is 8.33×10^-7^-9.69×10^-7^pg/T cell/hrs. IL-10 production is computed with Michaelis-Menten Kinetics:   IL-10 [new] = ((maximum production rate * CD4^+^ T cell [bind to APCs])/(CD4^+^ T cell count at which the reaction rate is half of maximum production rate + CD4^+^ T cell [bind to APCs])) × CD4^+^ T cell [bind to APCs]. Maximum production rate is equivalent to is 9.69×10^-7^ pg/CD4^+^ T cell /tick.   1. [Inactive] Apoptotic CD4^+^ T cells are phagocytosed by monocyte-derived-macrophage type I or monocyte-derived-macrophage type II. [Active] If they interact with monocyte-derived-macrophage type I or monocyte-derived-macrophage type II. 2. Undergo natural aging. The apoptotic rate needs to be estimated.   CD8 T cell. (Called *CD8TCell* in the ABM.)   1. [Inactive] Migrate from the portal triad to the liver sinusoid. [Active] if MDMIs or MDMIIs or both histamine and TNF-α are detected. The migration rate is 6.25×10^-2^/hrs. CD8^+^ T cell population is computed with a logistic equation:   CD8^+^ T cell [new] =influx rate * CD8^+^ T cell [old] *(1- CD8^+^ T cell [old]/ CD8^+^ T cell carrying capacity). CD8^+^ T cell carrying capacity = 250625.   1. [Inactivate] Apoptosis of Kupffer Cells, neutrophils, and monocyte-derived-macrophage type I who phagocytose *Salmonella*. [Active] If interact with APCs including Kupffer Cells, neutrophils, and monocyte-derived-macrophage type I. 2. [Inactive] Apoptotic CD8^+^ T cells are phagocytosed by monocyte-derived-macrophage type I or monocyte-derived-macrophage type II. [Active] If interact with monocyte-derived-macrophage type I or monocyte-derived-macrophage type II. 3. Undergo natural aging. The apoptotic rate needs to be estimated.   B cell. (Called *BCell* in the ABM.)   1. [Inactive] Migrate from the portal triad to the liver sinusoid. [Active] if MDMIs are detected. The migration rate is 4.30 ×10^-4^-2.40×10^-2^/hr. The B cell population is computed with a logistic equation:   B cell [new] =influx rate * B cell [old] *(1-B cell [old]/ B cell carrying capacity). B cell carrying capacity = 1433575.   1. [Inactivate] Produce antibody. [Activate] if they interact with CD4^+^ T cells. The rate is 4.88×10^-4^-2.81×10^-3^ pg/B cell/hr. 2. Undergo natural aging. The apoptotic rate needs to be estimated.   Antibody. (Called *Antibody* in the ABM.)   1. Antibodies are produced by B cells [See B cell 2]. 2. Interact with *Salmonella* to form antibody-opsonized *Salmonella*. The average amount of antibody binding to one *Salmonella* is 5.31 pg. 3. [With 20% probability] Antibody-opsonized *Salmonella* interact with mast cells [See Mast cell 2]. 4. [With 20% probability] Antibody-opsonized *Salmonella* interact with phagocytic cells including Kupffer Cells, or mast cells, or neutrophils, or monocyte-derived-macrophage type I, or monocyte-derived-macrophage type II. [Increase] Phagocytosis rate of if interaction with phagocytic cells is detected. [Set] Antibody decays if the interaction with phagocytic cells is detected.   CRP. (Called *CRP* in the ABM.)   1. [Inactive] Inhibits resting neutrophil recruitment. [Active] if CRP-opsonized apoptotic Kupffer Cells, CRP-opsonized apoptotic activated neutrophils, CRP-opsonized apoptotic MDMIs, or CRP-opsonized apoptotic MDMIIs. 2. CRP is released by hepatocytes [See Hepatocyte 3]. 3. [With 16.7% probability] CRP binds to *Salmonella*. 4. [With 20% probability] CRP-opsonized *Salmonella* are phagocytosed by phagocytic cells [See *Salmonella* 16] 5. [With 20% probability] CRP-opsonized apoptotic Kupffer Cells are phagocytosed by phagocytic cells [See Kupffer Cell 12]. 6. [With 20% probability] CRP-opsonized hepatocyte debris are phagocytosed by phagocytic cells [See Hepatocyte debris 2]. 7. [With 20% probability] CRP-opsonized apoptotic neutrophils are phagocytosed by phagocytic cells [See Activated neutrophil 4] 8. [With 20% probability] CRP-opsonized apoptotic monocyte-derived-macrophage type I are phagocytosed by phagocytic cells [See monocyte-derived-macrophage type I 6] 9. CRP-opsonized apoptotic monocyte-derived-macrophage type II are phagocytosed by phagocytic cells [See monocyte-derived-macrophage type II 6] 10. Undergo natural catabolism. The rate is 0.26/tick.   TNF-α. (Called *TN*F-αin the ABM.)   1. [See Kupffer Cell 5] 2. [See Monocyte-derived-macrophage type I 2] 3. [See Activated neutrophil 7] 4. [See Hepatocyte 7] 5. [See Mast cell 3] 6. Migrate to the nearest hepatocytes by random migration. 7. [See Hepatocyte 2] 8. Undergo natural catabolism. The catabolism rate needs to be estimated.   HMGB-1. (Called *HMGB-1* in the ABM.)   1. [See Monocyte-derived-macrophage type II 2] 2. [See Hepatocyte 6] 3. Undergo natural catabolism. The catabolism rate needs to be estimated.   IL-10. (Called *IL-10* in the ABM.)   1. [See Monocyte-derived-macrophage type II 3] 2. [See Activated neutrophil 8] 3. [See T cell 3] 4. [See Kupffer Cell 6, Activated neutrophil 10, Monocyte-derived-macrophage type I 10, Monocyte-derived-macrophage type II 10, Mast cell 5] 5. Undergo natural catabolism. The catabolism rate needs to be estimated.   Histamine. (Called *Histamine* in the ABM.)  [See Mast cell 2]  NETs. (Called *NETs* in the ABM.)  [See Activated neutrophil 9]  Sinusoid Endothelial Cell. (Called *SEC* in the model files.)  A auxiliary agent type which models edges of liver sinusoid. A boundary agent has a thin rectangular shape. *Salmonella* infect sinusoid endothelial cells with certain probability. The probability is determined by random movement of the *Salmonella* agent. SECs are infected by *Salmonella* [See *Salmonella* 5]  Signals. (Called *Signal* in the model files.)  An auxiliary agent type that help to recruit resting neutrophils or resting monocytes in the liver sinusoid. This represents all other factors not specifically represented in the model.  Anti-Signals. (Called *AntiSignal* in the model files.)  An auxiliary agent type that inhibit resting neutrophils or resting monocytes adhere to SECs. This represents all other factors not specifically represented in the model. |
