# Supplementary material for: An Agent-Based Model of a Hepatic Inflammatory Response to Salmonella: A Computational Study under a Large Set of Experimental Data
Source: PLoS One. 2016 Aug 24;11(8):e0161131. doi: 10.1371/journal.pone.0161131 (PMC4996536; doi:10.1371/journal.pone.0161131)
Supplement: S2 Table — (DOCX) [file pone.0161131.s004.docx]

Table S2. Agent Types and Agent Behaviors in IMMABM Based on Biological Behaviors (Agent types in “Agent Behavior(s)” are highlighted in Italic format, except terminology Salmonella enterica serovar Typhimurium (Salmonella) is Italic format in both “Biological Behavior(s)” and “Agent Behavior(s)”)

Note: biological behaviors that are lack of references (citations) indicate that the behaviors are implied from the literature in general.

| *Agent Type* (Biological Indictor) | Shape (s) in IMMABM | Biological Behavior(s) (BB) | Agent Behavior(s) (Netlogo process) | Agent Type(s) that the specific agent type interacts with |
| --- | --- | --- | --- | --- |
| 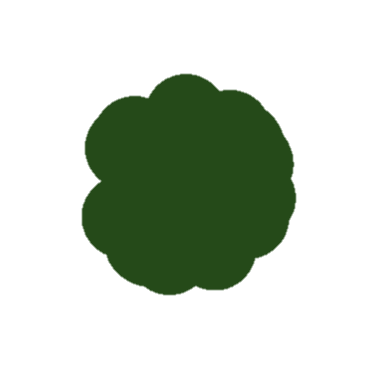*Salmonella* (*Salmonella*) |  | *Samonella* are phagocytized by Kupffer Cells [[1](#_ENREF_1), [2](#_ENREF_2)].  *Salmonella* are killed by Kupffer Cells [[1](#_ENREF_1), [2](#_ENREF_2)].  *Salmonella* replicate within apoptotic Kupffer Cells [[3](#_ENREF_3), [4](#_ENREF_4)].  *Salmonella* escape from apoptotic Kupffer Cells.  *Salmonella* infect SECs and replicate within SECs [[5](#_ENREF_5)].  *Salmonella* released from infected SECs persistently infect and replicate within neighboring cells.  Released *Salmonella* infect healthy hepatocytes [[5](#_ENREF_5), [6](#_ENREF_6)].  *Salmonella* replicate within infected hepatocytes [[5](#_ENREF_5), [6](#_ENREF_6)].  *Salmonella* escape from apoptotic hepatocytes [[7](#_ENREF_7)].  *Salmonella* released from infected hepatocytes and persistently infect and replicate within liver tissue.  Released *Salmonella* are phagocytized by neutrophils [[2](#_ENREF_2), [7](#_ENREF_7)].  *Salmonella* are killed by neutrophils [[2](#_ENREF_2), [7](#_ENREF_7)].  Escaped *Salmonella* are phagocytized by monocyte-derived-macrophage type I (MDMI) [[3](#_ENREF_3), [8](#_ENREF_8)].  *Salmonella* are killed by monocyte-derived-macrophage type I [[3](#_ENREF_3), [8](#_ENREF_8)].  *Salmonella* replicate within monocyte-derived-macrophage type I [[9](#_ENREF_9)].  *Salmonella* are trapped and killed by neutrophil extracellular traps (NETs) as a complex of Myeloperoxidase (MPO) and neutrophil elastase (NE). MPO and NE are released from neutrophil degranulation [[10-12](#_ENREF_10)].  *Salmonella* bind to mast cells [[13](#_ENREF_13), [14](#_ENREF_14)].  *Salmonella* that bind CRP are killed by Kupffer Cells [[15](#_ENREF_15)].  *Salmonella* that bind CRP are killed by mast cells [[15](#_ENREF_15)].  *Salmonella* that bind CRP are killed by neutrophils [[15](#_ENREF_15)].  *Salmonella* that bind CRP are killed by macrophages [[15](#_ENREF_15)].  *Salmonella* that bind by antibody are killed by Kupffer Cells [[16](#_ENREF_16)].  *Salmonella* that bind by antibody are killed by mast cells [[16](#_ENREF_16)].  *Salmonella* that bind by antibody are killed by neutrophils [[16](#_ENREF_16)].  *Salmonella* that bind by antibody are killed by macrophages [[16](#_ENREF_16)].  *Salmonella* growth is inhibited by other undefined mechanism [[17](#_ENREF_17)]. | *Salmonella*PhagocytizeByKupfferCellSubRoutine [BB.1]  *Salmonella*KillByKupfferCellSubRoutine [BB.2]  *Salmonella*ReplicateWithinKupfferCellSubRoutine [BB.3]  *Salmonella*ReplicateWithinSECsSubRoutine [BB. 5]  *Salmonella*ReplicateWithinHepatocyteSubRoutine [BB.7, BB.8]  *Salmonella*PhagocytizeByActivatedNeutrophilSubRoutine [BB. 11, BB. 12]  *Salmonella*PhagocytizeByMDMISubRoutine [BB. 13, BB.14]  *Salmonella*ReplicateWithinMDMISubRoutine [BB. 15]  newlyReleased*Salmonella*FromApoptoticCellsInteractWithSECsOrHepatocyteOrMastCellSubRoutine [BB. 4, BB.6, BB.9, BB. 10, BB. 17]  *Salmonella*GetTrappedByNETsSubRoutine [BB. 16]  For BB. 18, see CRP Behaviors(s). 3  For BB. 19, see CRP Behaviors(s). 4  For BB. 20, see CRP Behaviors(s). 5  For BB. 21, see CRP Behaviors(s). 6 and 7  For BB. 22, see Antibody Behaviors(s). 4  For BB. 23, see Antibody Behaviors(s). 5  For BB. 24, see Antibody Behaviors(s). 6  For BB. 25, see Antibody Behaviors(s). 7 and 8  *Salmonella*DieBecauseOtherChemicalsReleaseByMastCellSubRoutine [BB. 26] | 1. *KupfferCell*  2. *Hepatocyte*  3. *SEC*  4. *ActivatedNeutrophil*  5. *NET*  6. *MDMI*  7. *MastCell*  8. *Antibody*  9. *CRP* |
| 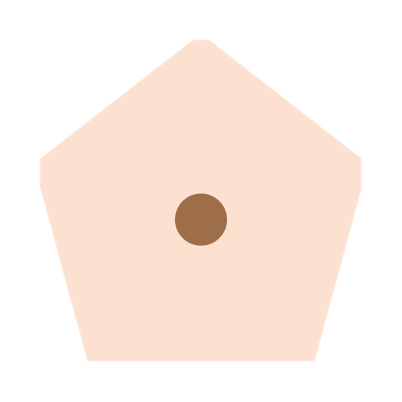*Hepatocyte* (Hepatocyte) |  | Hepatocytes are infected by *Salmonella* and undergo apoptosis [[2](#_ENREF_2), [5](#_ENREF_5)]. Apoptotic hepatocytes become hepatocyte debris.  Hepatocytes are activated with TNF-α and become apoptotic [[18](#_ENREF_18)]. Apoptotic hepatocytes become hepatocyte debris.  CRP is released by hepatocytes in response to IL-6 released by macrophages [[19](#_ENREF_19), [20](#_ENREF_20)].  *Salmonella*-infected hepatocytes interact with activated neutrophils which accelerates the apoptosis [[21-23](#_ENREF_21)].  Apoptotic hepatocytes release TNF-α [[24](#_ENREF_24)].  Apoptotic hepatocytes release HMGB1 [[25](#_ENREF_25)].  Hepatocytes regenerate [[26](#_ENREF_26)]. | hepatocyteBecomeHepatocyteDebrisInducedBy*Salmonella*SubR-outine [BB. 1]  hepatocyteBecomeHepatocyteDebrisInducedByTNFAlphaSubRo-utine [BB. 2]  hepatocyteReleaseCRPIfAnyMDMIWasDetectedSubRoutine [BB. 3]  apoptoticHepatocyteKillByNeutrophilSubRoutine [BB. 4]  apoptoticHepatocyteProduceTNFAlphaSubRoutine [BB. 5]  apoptoticHepatocyteProduceHMGB1SubRoutine [BB. 6]  hepatocyteRegenerateSubRoutine [BB. 7] | 1*. Salmonella*  2. *TNF-α*  3. *ActivatedNeutrophil* |
| 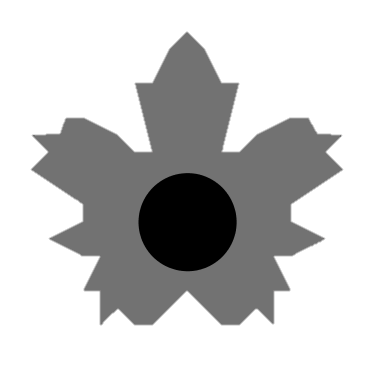*HepatocyteDebris* (Hepatocyte debris) |  | Hepatocyte debris is phagocytized by Kupffer Cells [[18](#_ENREF_18)].  Hepatocyte debris is phagocytized by mast cells.  Hepatocyte debris can also be phagocytized by neutrophils [[21](#_ENREF_21)].  Hepatocyte debris can also be phagocytized by monocyte-derived-macrophage type I [[27](#_ENREF_27), [28](#_ENREF_28)].  Hepatocyte debris can also be phagocytized by monocyte-Derived-Macrophage Type II (MDMII) [[27](#_ENREF_27), [28](#_ENREF_28)].  Hepatocyte debris bind to CRP [[15](#_ENREF_15)].  Hepatocyte debris binding to CRP are phagocytized by inflammatory cells including Kupffer Cells, neutrophils, mast cells, MDMI, and MDMII [[15](#_ENREF_15)].  Hepatocyte debris undergo natural degradation. | hepatocyteDebrisPhagocytizeByInflammatoryCellSubRoutine [BB. 1, BB.2, BB. 3, BB. 4, and BB. 5]  For BB. 6, see CRP Behaviors(s). 14  For BB. 7, see CRP Behaviors(s). 15, 16, 17, 18, and 19  hepatocyteDebrisDieByNatureSubRoutine [BB. 8] | 1. *KupfferCell*  2. *MastCell*  2. *ActivatedNeutrophil*  3. *MDMI*  4. *MDMII*  5. *CRP* |
| 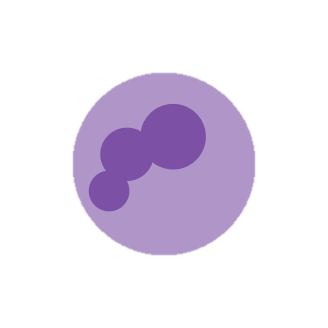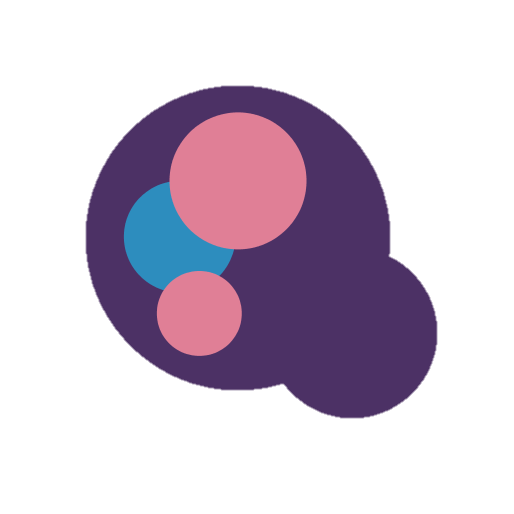*RestingNeutrophil* (Circulating neutrophil) |  | Circulating neutrophil numbers increase after infection [[29](#_ENREF_29)].  Circulating neutrophils roll along sinusoidal endothelial cells [[9](#_ENREF_9), [30](#_ENREF_30)].  Circulating neutrophils get signals from sinusoidal endothelial cells [[9](#_ENREF_9), [30](#_ENREF_30)].  Circulating neutrophils are activated by TNF-α, *Salmonella*, and HMGB-1 [[21](#_ENREF_21), [31-34](#_ENREF_31)].  Circulating neutrophils undergo aging and undergo apoptosis [[35](#_ENREF_35)]. | restingNeutrophilInfluxToLiverSinusoidFromBoneMarrowSubRou-tine [BB. 1]  restingNeutrophilMoveToSECsFollowingSignalSentFromCytokineAndGetActivatedSubRoutine [BB. 2, BB. 3, and BB. 4]  restingNeutrophilUndergoAgingByNatureSubRoutine [BB. 5] | *SEC* |
| *ActivatedNeutrophil* (Activated neutrophil) |  | Activated circulating neutrophils adhere to sinusoidal endothelial cells [[9](#_ENREF_9), [30](#_ENREF_30)].  Activated neutrophils migrate to liver Kupffer Cells [[36](#_ENREF_36)].  Activated neutrophils interact with *Salmonella* [[7](#_ENREF_7), [36](#_ENREF_36), [37](#_ENREF_37)].  Activated neutrophils migrate to apoptotic hepatocytes which are infected by *Salmonella* [[7](#_ENREF_7)].  Activated neutrophils undergo natural aging [[38](#_ENREF_38), [39](#_ENREF_39)].  Apoptotic neutrophils interact with CRP [[15](#_ENREF_15)].  Apoptotic neutrophils interacted with CRP are phagocytized by inflammatory cells [[15](#_ENREF_15)]. Apoptotic neutrophils die after phagocytosis.  Activated neutrophils inhibit neutrophil movement to the site of infection by taking up CRP that has bound to cell debris [see CRP 1].  Apoptotic neutrophils are engulfed by MDMII [[9](#_ENREF_9)].  Activated neutrophils release cytokines such as TNF-α and IL-10 after neutrophil simulation by bacterial LPS [[40-42](#_ENREF_40)].  Activated neutrophils undergo degranulation and release MPO and NE after neutrophils simulation by bacterial LPS [[10](#_ENREF_10)].  Activated neutrophils release NETs [[11](#_ENREF_11)].  Activated neutrophils bind to IL-10 [[43](#_ENREF_43)] [see model assumption 16].  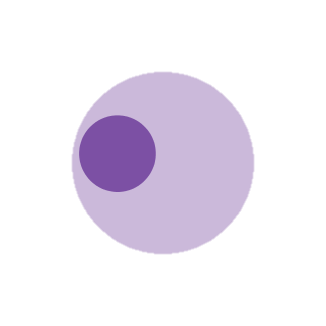Activated neutrophils that phagocytize *Salmonella* undergo apoptosis if bind to CD8^+^ T cells [[16](#_ENREF_16)]. | restingNeutrophilMoveToSECsFollowingSignalSentFromCytokineAndGetActivatedSubRoutine [BB. 1, BB. 8]  activatedNeutrophilInteractWithKupfferCellSubRoutine [BB. 2]  activatedNeutrophilPhagocytizeByKupfferCellSubRoutine [BB. 2]  activatedNeutrophilMoveToSiteOf*Salmonella*SubRoutine [BB. 3]  activatedNeutrophilMoveToSiteOfApoptoticHepatocyteSubRoutine [BB. 4]  activatedNeutrophilUndergoAgingByNatureSubRoutine [BB. 5]  For BB. 6, see CRP Behaviors(s). 20  For BB. 7, see CRP Behaviors(s). 21, 22, 23, 24, and 25  apoptoticActivatedNeutrophilPhagocytizeByMDMII [BB. 9]  activatedNeutrophilProduceTNFAlphaSubRoutine [BB. 10]  activatedNeutrophilProduceIL10SubRoutine [BB. 11]  activatedNeutrophilProduceNETsSubRoutine [BB. 12]  For BB. 13, see IL-10 Behavior(s). 4 and 5  activatedNeutrophilWhoPhagocytize*Salmonella*UndergoApoptosisByInteractWithCD8TCellSubRoutine [BB. 14] | 1. *SEC*  2. *KupfferCell*  3. *Salmonella*  4. *Hepatocyte*  5. *CRP*  6. *IL-10*  7. *MDMI*  8*. MDMII*  9*. MastCell*  10*. ActivatedNeutrophil* |
| *RestingMonocyte* (Circulating monocyte) |  | Circulating monocytes are released after infection [[44](#_ENREF_44)].  Circulating monocytes roll in the blood vessel [[9](#_ENREF_9), [45](#_ENREF_45)].  Circulating monocytes get signals and are activated to adhere to endothelial cells [[9](#_ENREF_9), [45](#_ENREF_45), [46](#_ENREF_46)].  Circulating monocytes are activated by TNF-α, *Salmonella*, HMGB-1, and T_H_1 effector cells and apoptotic activated neutrophils [[9](#_ENREF_9), [29](#_ENREF_29), [32](#_ENREF_32), [36](#_ENREF_36), [45](#_ENREF_45), [47-51](#_ENREF_47)].  Monocytes become MDMIs when they encounter *Salmonella* or TNF-α [[9](#_ENREF_9), [52](#_ENREF_52)].  Monocytes become MDMIIs when they encounter apoptotic activated neutrophils [[9](#_ENREF_9), [52](#_ENREF_52)].  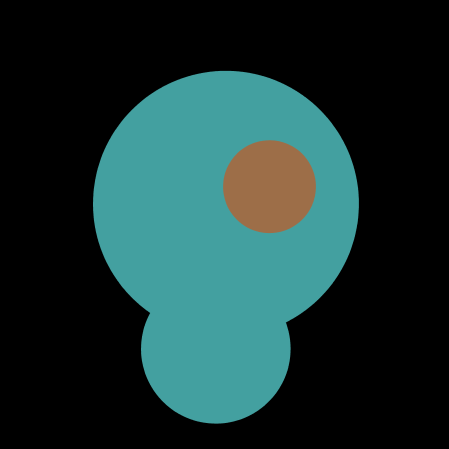Circulating monocytes undergo aging and undergo apoptosis [[53](#_ENREF_53)]. | restingMonocyteInfluxToLiverSinusoidFromBoneMarrowSubRou-tine [BB. 1]  restingMonocyteMoveToSECsFollowingSignalSentFromCytokineAndGetActivatedSubRoutine [BB. 2, BB. 3, and BB. 4]  restingMonocyteBecomeMDMIOrMDMIIByInteractWithCorresp-ondingCellO-rCytokineSubRoutine [BB. 5, BB. 6]  restingMonocyteUndergoAgingByNatureSubRoutine [BB. 7] | 1. *Salmonella*  2. *TNF-α*  3. *ActivatedNeutrophil*  4. *SEC* |
| *MDMI* (monocyte-Derived-Macrophage Type I) |  | MDMIs migrate to the site of *Salmonella* [[9](#_ENREF_9), [52](#_ENREF_52)].  MDMIs phagocytize (kill) *Salmonella* at certain rate [[9](#_ENREF_9), [52](#_ENREF_52)].  MDMIs release TNF-ɑ [[9](#_ENREF_9)].  MDMIs are killed by *Salmonella* [[3](#_ENREF_3), [8](#_ENREF_8)].  MDMIs undergo natural aging [[54](#_ENREF_54)].  Aging MDMIs interact with CRP [[15](#_ENREF_15)].  Aging MDMIs interacted with CRP are phagocytized by inflammatory cells [[15](#_ENREF_15)].  MDMIs phagocytize CRP bound to cell debris and release cytokines such as TNF-α [[15](#_ENREF_15), [55](#_ENREF_55)].  MDMIs (partially) transform to Kupffer Cells [[56-58](#_ENREF_56)].  MDMIs bind to IL-10 [See model assumption 16].  MDMIs, as APCs, attract T cells from lymph node to the site of infection [[59](#_ENREF_59)].  MDMIs phagocytize apoptotic T cells [[60](#_ENREF_60)].  MDMIs release IL-10 by phagocytizing apoptotic T cells [[60](#_ENREF_60)].  MDMIs that phagocytize *Salmonella* undergo apoptosis if they bind to CD8^+^ T cells [[16](#_ENREF_16)]. | restingMonocyteBecomeMDMIOrMDMIIByInteractWithCorresp-ondingCellO-rCytokineSubRoutine [BB. 1]  MDMIPhagocytize*Salmonella*SubRoutine [BB. 2]  MDMIProduceTNFAlphaByPhagocytize*Salmonella*OrCRPTypeComplexSubRoutine [BB. 3, BB. 8]  MDMIKillBy*Salmonella*SubRoutine [BB. 4]  MDMIUndergoAgingByNatureSubRoutine [BB. 5]  For BB. 6, see CRP Behavior(s). 26  For BB. 7, see CRP Behavior(s). 27, 28, 29, 30, and 31  MDMITransformToKupfferCellSubRoutine [BB. 9]  For BB. 10, see IL-10 Behavior (s). 4 and 5  For BB. 11, see CD4TCellBehavior(s). 1 and CD8TCellBehavior (s). 1  For BB. 12, see CD4TCellBehavior(s). 4 and CD8TCellBehavior (s). 3  MDMIProduceIL10ByPhagocytizeApoptoticCD4TCellOrCD8TCellS-ubRoutine [BB. 13]  MDMIWhoPhagocytize*Salmonella*UndergoApoptosisByInteract-WithCD8TCellSubRoutine [BB. 14] | 1. *Salmonella*  2. *CRP*  3. *IL-10*  4*. KupfferCell*  5*. ActivatedNeutrophil*  6*. MastCell*  7*. MDMI*  8*. MDMII*  9*. CD8TCell*  10. Hepatocyte |
| 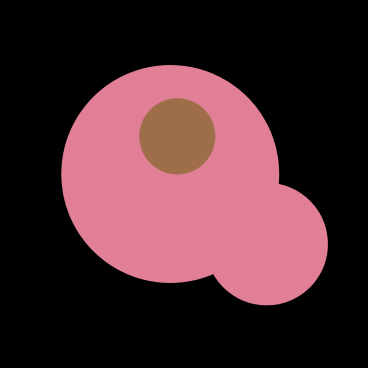*MDMII* (monocyte-Derived-Macrophage Type II) |  | MDMIIs migrate to apoptotic activated neutrophils [[9](#_ENREF_9), [47](#_ENREF_47), [48](#_ENREF_48)].  MDMIIs phagocytize apoptotic activated neutrophils [[9](#_ENREF_9), [47](#_ENREF_47), [48](#_ENREF_48)].  MDMIIs release HMGB-1 after phagocytizing apoptotic activated neutrophils [[9](#_ENREF_9)].  MDMIIs release IL-10 after phagocytizing apoptotic activated neutrophils [[9](#_ENREF_9)].  MDMIIs phagocytize apoptotic T cells [[60](#_ENREF_60)].  MDMIIs release IL-10 after phagocytizing apoptotic T cells [[60](#_ENREF_60)].  MDMIIs undergo natural aging [[54](#_ENREF_54)].  Aging MDMIIs bind CRP [[15](#_ENREF_15)].  Aging MDMIIs that bind CRP are phagocytized by inflammatory cells [[15](#_ENREF_15)].  MDMIIs phagocytize CRP bound to cell debris and release cytokines [[15](#_ENREF_15)].  MDMIIs (partially) transform to Kupffer Cells [[56-58](#_ENREF_56)].  MDMIIs *bind* IL-10 [See model assumption 16].  MDMIIs, as APCs, attract T cells from lymph node to the site of infection [[59](#_ENREF_59)]. | restingMonocyteBecomeMDMIOrMDMIIByInteractWithCorresp-ondingCellO-rCytokineSubRoutine [BB. 1]  MDMIIPhagocytizeApoptoticNeutrophilSubRoutine [BB. 2]  MDMIIProduceHMGB1ByPhagocytizeApoptoticNeutrophilSubRoutine [BB. 3]  MDMIIProduceIL10ByPhagocytizeApoptoticNeutrophilOrApoptoticTCellSubRoutine [BB. 4, BB. 6]  For BB. 5, see CD4TCellBehavior(s). 4 and CD8TCellBehavior(s). 3  MDMIIUndergoAgingByNatureSubRoutine [BB. 7]  For BB. 8, see CRP Behavior(s). 32  For BB. 9, see CRP Behavior(s). 33, 34, 35, 36 and 37  For BB. 10, see CRP Behavior(s). 7  MDMIITransformToKupfferCellSubRoutine [BB. 11]  For BB. 12, see IL-10 Behavior(s). 4 and 5  For BB. 13, see CD4TCellBehavior(s). 1 and CD8TCellBehavior(s). 1 | 1. *ActivatedNeutrophil*  2. *CRP*  3. *IL-10*  4. *CD4TCell*  5. *CD8TCell*  6*. KupfferCell*  7*. MastCell*  8*. MDMI*  9*. MDMII*  10. *Hepatocyte* |
| *TNF-ɑ* (Tumor necrosis 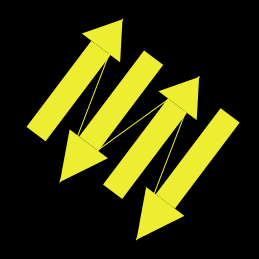factor alpha) |  | TNF-α is released by Kupffer Cells upon interacting with *Salmonella* [[36](#_ENREF_36)].  TNF-α is released by Kupffer Cells upon interacting with hepatocyte debris [[18](#_ENREF_18)].  TNF-α is released by Kupffer Cells upon interacting with activated neutrophils [[36](#_ENREF_36)].  TNF-α is released by MDMIs [[9](#_ENREF_9), [52](#_ENREF_52)].  TNF-α is released by activated neutrophils [[42](#_ENREF_42)].  TNF-α is released by apoptotic hepatocytes [[61](#_ENREF_61)].  TNF-α is released by mast cells [[62](#_ENREF_62), [63](#_ENREF_63)].  TNF- α migrates to hepatocytes [[18](#_ENREF_18)].  TNF- α damages healthy hepatocytes [[18](#_ENREF_18)].  TNF-ɑ undergoes natural catabolism [[64-66](#_ENREF_64)]. | For BB. 1, see KupfferCell Behavior(s). 2  For BB. 2, see KupfferCell Behavior(s). 2  For BB. 3, see KupfferCell Behavior(s). 2  For BB. 4, see MDMI Behavior(s). 3  For BB. 5, see ActivatedNeutrophil Behavior(s). 10  For BB. 6, see Hepatocyte Behavior(s). 5  For BB. 7, see MastCell Behavior(s). 3 and 4  For BB. 8, see Hepatocyte Behavior(s). 2  For BB. 9, see Hepatocyte Behavior(s). 2  TNFAlphaUndergoCatabolismByNatureSubRoutine [BB. 10] | *Hepatocyte* |
| *HMGB-1* (High mobility group protein B1) | 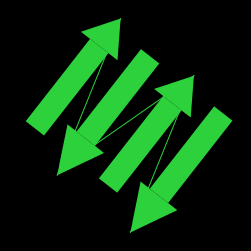 | HMGB-1 is released by MDMIIs in response to apoptotic neutrophils [[67](#_ENREF_67), [68](#_ENREF_68)].  HMGB-1 is released by apoptotic hepatocytes [[32](#_ENREF_32)].  HMGB-1 undergoes natural catabolism [[69-71](#_ENREF_69)]. | For BB. 1, see MDMII Behavior(s). 3  For BB. 2, see Hepatocyte Behavior(s). 6  HMGB1UndergoCatabolismByNatureSubRoutine [BB. 3] |  |
| 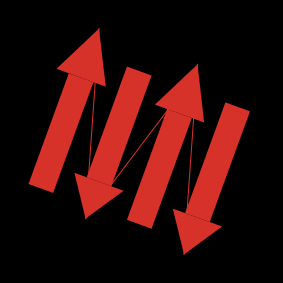*IL-10* (Interleukin 10) |  | IL-10 is produced by MDMIIs [[9](#_ENREF_9), [52](#_ENREF_52), [71](#_ENREF_71)].  IL-10 is released by activated neutrophils [[40](#_ENREF_40), [41](#_ENREF_41)].  IL-10 is produced by T cells [[34](#_ENREF_34)].  IL-10 diffuses to the site of Kupffer Cells and inhibits the release of TNF-ɑ.  IL-10 diffuses to the site of activated neutrophils and inhibits the release of TNF-α [[43](#_ENREF_43)].  IL-10 diffuses to the site of MDMI*s* and inhibits the release of TNF-ɑ [[71](#_ENREF_71)].  IL-10 diffuses to the site of MDMIIs and inhibits the release of HMGB-1 [[72-75](#_ENREF_72)].  IL-10 diffuses to the site of MDMIIs and inhibits the release of IL-10 [[72-75](#_ENREF_72)].  IL-10 diffuses to the site of mast cells and inhibits the release of TNF-ɑ [see model assumption 16].  IL-10 undergoes natural catabolism [[76-78](#_ENREF_76)]. | For BB. 1, see MDMII Behavior(s). 4  For BB. 2, see ActivatedNeutrophil Behavior(s). 11  For BB. 3, see CD4TCell Behavior(s). 3  IL10InteractWithInflammatoryCellsSubRoutine [BB. 4, BB. 5, BB. 6, BB. 7, BB. 8, and BB. 9]  IL10BindToInflammatoryCellsSubRoutine [BB. 4, BB. 5, BB. 6, BB. 7, BB. 8, and BB. 9]  IL10UndergoCatabolismByNatureSubRoutine [BB. 10] | 1. *KupfferCell*  2. *MDMI*  3. *MDMII*  4. *ActivatedNeutrophil*  5*. MastCell* |
| *KupfferCell* (Kupffer Cell) | 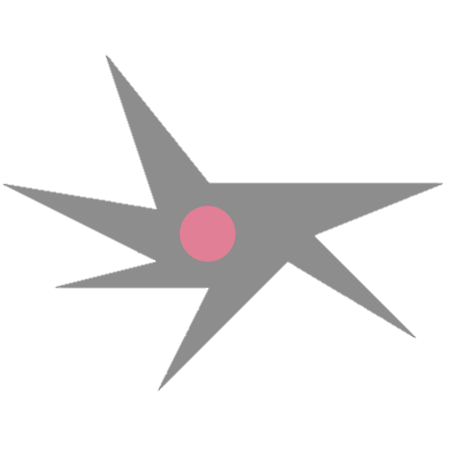 | Kupffer Cells are killed by *Salmonella* [[3](#_ENREF_3), [4](#_ENREF_4)].  Kupffer Cells release TNF-α after interacting with *Salmonella* [[79](#_ENREF_79)].  Kupffer Cells release TNF-ɑ after phagocytizing hepatocyte debris [[18](#_ENREF_18)].  Kupffer Cells release TNF-α after interacting with activated neutrophils [[36](#_ENREF_36), [80](#_ENREF_80)].  Kupffer Cells bind to IL-10 [[72](#_ENREF_72)] [See model assumption 16].  Kupffer Cells are replaced by monocyte-Derived MDMIs/MDMIIs [[56-58](#_ENREF_56)].  Kupffer Cells undergo natural apoptosis [[57](#_ENREF_57), [58](#_ENREF_58)].  Apoptotic Kupffer Cells interact with CRP [[15](#_ENREF_15)].  Apoptotic Kupffer Cells decorated with CRP are phagocytized by inflammatory cells [[15](#_ENREF_15)]. Apoptotic Kupffer Cells die after phagocytosis.  Kupffer Cells phagocytize CRP-opsonized cell debris and inhibit the production of TNF-α. The phagocytic ability of Kupffer Cells is enhanced by CRP-opsonized particles [[15](#_ENREF_15)] [see model assumption 21].  Kupffer Cells that phagocytize *Salmonella* undergo apoptosis if they bind to CD8^+^ T cells [[16](#_ENREF_16)].  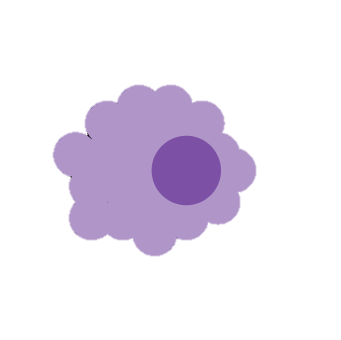Kupffer Cells Release IL-10 [[20](#_ENREF_20)]. | kupfferCellKillBy*Salmonella*SubRoutine [BB. 1]  kupfferCellProduceTNFAlphaInteractWith*Salmonella*OrHepatocyteDebrisOrActivatedNeutrophilSubRoutine [BB. 2, BB. 3, and BB. 4]  For BB. 5, see IL-10 Behavior(s). 4 and 5  For BB. 6, see MDMI Behavior(s). 8 and MDMII Behavior(s). 10  kupfferCellUndergoAgingByNature [BB. 7]  For BB. 8, see CRP Behavior(s). 8  For BB. 9, see CRP Behavior(s). 9, 10, 11, 12, and 13  For BB. 10, see KupfferCell Behavior(s). 2  kupfferCellWhoPhagocytize*Salmonella*UndergoApoptosisByInteractWithCD8TCellSubRoutine [BB. 11]  kupfferCellProduceIL10ByIngestApoptoticHepatocyteSubRoutine [BB. 12] | 1. *Salmonella*  2. *HepatocyteDebris*  3*. Hepatocyte*  4. *ActivatedNeutrophil*  5. *IL-10*  6. *CRP*  7*. KupfferCell*  8*. MastCell*  9*. MDMI*  10*. MDMII*  11*. CD8TCell* |
| *MastCell* (Mast cell) |  | Mast cells self-renewal [[13](#_ENREF_13), [81](#_ENREF_81)].  Mast cell degranulation is activated by interacting with a complex of antibody and *Salmonella* [[82](#_ENREF_82)].  Mast cells release histamine by degranulation [[82](#_ENREF_82), [83](#_ENREF_83)].  Mast cells binding to *Salmonella* release TNF-α [[82](#_ENREF_82)].  Mast cells release TNF-α upon interacting with antibody-opsonized *Salmonella* [[62](#_ENREF_62), [63](#_ENREF_63)].  TNF-α and histamine help to recruit T cells from lymph node to the site of infection [[82](#_ENREF_82), [84](#_ENREF_84)].  Mast cells bind to IL-10 and fail to release TNF-α [see model assumption 16].  Mast cells undergo natural aging [[16](#_ENREF_16)]. | mastCellProliferateSubRoutine [BB. 1]  mastCellProduceHistamineByInteractWithAntibody*Salmonella*ComplexSubRoutine [BB. 2, BB. 3]  mastCellProduceTNFAlphaByBindingTo*Salmonella*SubRoutine [BB. 4]  mastCellProduceTNFAlphaByInteractWithAntibody*Salmonella*ComplexSubRoutine [BB. 5]  For BB. 6, see CD4TCell Behavior(s). 1 and CD8TCellBehavior (s). 2  For BB. 7, see IL-10 Behavior(s). 4 and 5  mastCellUndergoApoptosisByNatureSubRoutine [BB. 8] | 1. *Salmonella*  2. *Antibody*  3. *ActivatedNeutrophil*  4*. Hepatocyte*  5*. MastCell*  6*. MDMI*  7*. MDMII*  8. *CD4Tcell*  9. *CD8Tcell* |
| *CD4TCell* (CD4 T cell) |  | CD4^+^ T cells in lymph node [[16](#_ENREF_16)].  CD4^+^ T cells are activated by APCs to proliferate 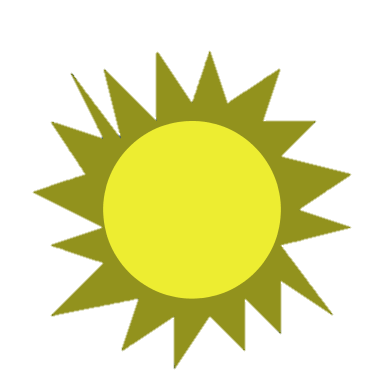and differentiate into T_H_1 effector cells to release TNF-α [[34](#_ENREF_34), [60](#_ENREF_60)].  CD4^+^ T cells are activated by APCs to proliferate and differentiate into T_H_2 effector cells to release IL-10 [[34](#_ENREF_34)].  T_H_1 effector cells activate infected Kupffer Cells to kill *Salmonella* [[34](#_ENREF_34)].  T_H_1 effector cells activate infected macrophages to kill *Salmonella* [[34](#_ENREF_34)].  Apoptotic CD4^+^ T cells are phagocytized by MDMIIs [[59](#_ENREF_59)].  CD4^+^ T cells (fails to bind to APCs) undergo apoptosis [[34](#_ENREF_34)]. | CD4TCellMigrateFromPortalTriadToLiverSinusoidSubRoutine [BB. 1]  CD4TCellProduceTNFAlphaByInteractWithAPCsSubRoutine [BB. 2, BB. 4, and BB. 5]  CD4TCellProduceIL10ByInteractWithAPCsSubRoutine [BB. 3]  apoptoticCD4TCellPhagocytizeByMDMI0rMDMIISubRoutine [BB. 6]  CD4TCellUndergoAgingByNatureSubRoutine [BB. 7] | 1. *MastCell*  2. *ActivatedNeutrophil*  3. *KupfferCell*  4. *MDMII* |
| 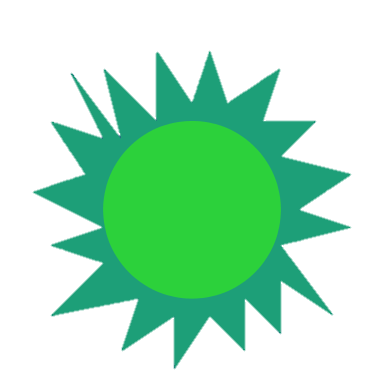 *CD8TCell* (CD8 T cell) |  | CD8^+^ T cells in lymph node [[16](#_ENREF_16)].  CD8^+^ T cells induce the apoptosis of infected cells (infected cells are the inflammatory cells that *Salmonella* inhabit) [[16](#_ENREF_16), [34](#_ENREF_34), [60](#_ENREF_60)].  Apoptotic CD8^+^ T cells are phagocytized by MDMIIs [[59](#_ENREF_59)].  CD8^+^ T cells (fails to bind to APCs) undergo apoptosis [[34](#_ENREF_34)]. | CD8TCellMigrateFromPortalTriadToLiverSinusoidSubRoutine [BB. 1]  CD8TCellInduceApoptosisOfKupfferCellOrNeutrophilOrMDMIWhoPhagocytize*Salmonella*SubRoutine [BB. 2]  apoptoticCD8TCellPhagocytizeByMDMIOrMDMIISubRoutine [BB. 3]  CD8TCellUndergoAgingByNatureSubRoutine [BB. 4] | 1. *MastCell*  2. *ActivatedNeutrophil*  3. *KupfferCell*  4. *MDMII* |
| 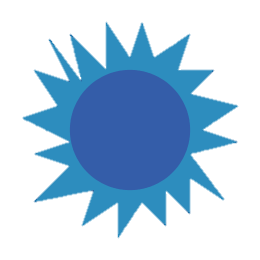*BCell* (B cell) |  | B cells in lymph node [[85](#_ENREF_85)].  B cells are activated by T_H_1 or T_H_2 cells to proliferate and release antibody [[34](#_ENREF_34)].  B cell (fails to bind to T helper cells or antigen) undergo apoptosis [[34](#_ENREF_34)]. | BCellMigrateFromPortalTriadToLiverSinusoidSubRoutine [BB. 1]  BCellProduceAntibodySubRoutine [BB. 2]  BCellUndergoAgingByNatureSubRoutine [BB. 3] | 1. *CD4TCell* |
| 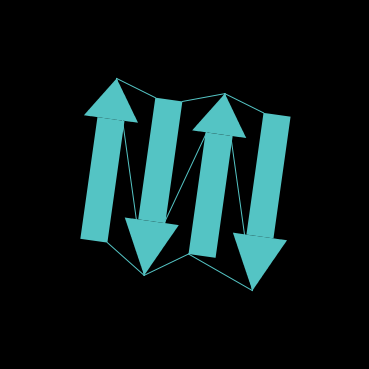*Antibody* (Antibody) | 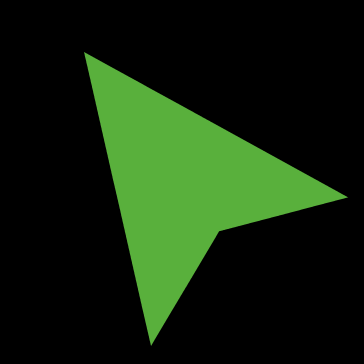 | Antibody is released from activated B cells [[34](#_ENREF_34)].  Antibody Interacts with *Salmonella* (multiple antibodies to one *Salmonella*) [[16](#_ENREF_16)].  Antibody-opsonized *Salmonella* interacts with mast cells and initiates the degranulation of mast cells [[82](#_ENREF_82)].  Antibody-bound to *Salmonella* is phagocytized by Kupffer Cells (higher phagocytosis rate here). This process refers to opsonization [[16](#_ENREF_16)].  Antibody-bound to *Salmonella* is phagocytized by mast cells (higher phagocytosis rate here). This process refers to opsonization [[16](#_ENREF_16)].  Antibody-bound to *Salmonella* is phagocytized by neutrophils (higher phagocytosis rate here) [[16](#_ENREF_16)].  Antibody-bound to *Salmonella* is phagocytized by macrophage-derived-macrophage type I (higher phagocytosis rate here) [[16](#_ENREF_16)].  Antibody-bound by *Salmonella* is phagocytized by macrophage-derived-macrophage type II (higher phagocytosis rate here) [[16](#_ENREF_16)]. | For BB. 1, see BCell Behavior(s). 2  antibodyInteractWith*Salmonella*SubRoutine [BB. 2]  For BB. 3, see MastCell Behavior(s). 4  antibody*Salmonella*ComplexPhagocytizeByKupfferCellSubRoutine [BB. 4]  antibody*Salmonella*ComplexPhagocytizeByMastCellSubRoutine [BB. 5]  antibody*Salmonella*ComplexPhagocytizeByNeutrophilSubRoutine [BB. 6]  antibody*Salmonella*ComplexPhagocytizeByMDMISubRoutine [BB. 7]  antibody*Salmonella*ComplexPhagocytizeByMDMIISubRoutine [BB. 8] | 1. *MastCell*  2. *Salmonella*  3. *KupfferCell*  4. *ActivatedNeutrophil*  5. *MDMI*  6*. MDMII* |
| *CRP* (C-reactive protein) | 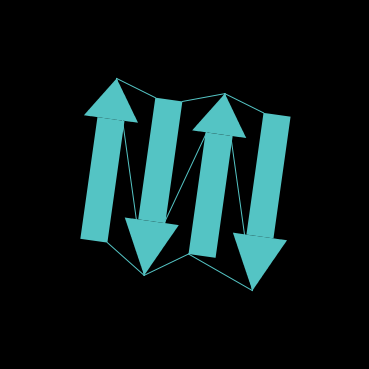 | CRP inhibits neutrophil movement to the site of infection in a human model with adult respiratory distress syndrome [[86](#_ENREF_86), [87](#_ENREF_87)].  CRP is released by hepatocytes in response to IL-6 released by macrophages [[19](#_ENREF_19), [20](#_ENREF_20)].  CRP-bound *Salmonella* are phagocytized by Kupffer Cells [[15](#_ENREF_15)].  CRP-bound *Salmonella* are phagocytized by mast cells [[15](#_ENREF_15)].  CRP-bound *Salmonella* are phagocytized by neutrophils [[15](#_ENREF_15)].  CRP-bound *Salmonella* are phagocytized by MDMIs [[15](#_ENREF_15)].  CRP-bound *Salmonella* are phagocytized by MDMIIs [[15](#_ENREF_15)].  CRP binds to apoptotic Kupffer Cells [[15](#_ENREF_15)].  CRP-bound apoptotic Kupffer Cells are phagocytized by inflammatory cells [[15](#_ENREF_15)].  CRP binds to apoptotic hepatocytes [[15](#_ENREF_15)].  CRP-bound apoptotic hepatocytes are phagocytized by inflammatory cells [[15](#_ENREF_15)].  CRP binds to apoptotic neutrophils [[15](#_ENREF_15)].  CRP-bound apoptotic neutrophils are phagocytized by inflammatory cells [[15](#_ENREF_15)].  CRP binds to apoptotic monocyte-derived-macrophage type I [[15](#_ENREF_15)].  CRP-bound apoptotic monocyte-derived-macrophage type I are phagocytized by inflammatory cells [[15](#_ENREF_15)].  CRP binds to apoptotic monocyte-derived-macrophage type II [[15](#_ENREF_15)].  CRP-bound apoptotic monocyte-derived-macrophage type II are phagocytized by inflammatory cells [[15](#_ENREF_15)].  CRP undergo natural catabolism [[88](#_ENREF_88)]. | For BB. 1, see RestingNeutrophil Behavior(s). 2  For BB. 2, see Hepatocyte Behavior(s). 3  CRP*Salmonella*ComplexPhagocytizeBykupfferCellSubRoutine [BB. 3]  CRP*Salmonella*ComplexPhagocytizeByMastCellSubRoutine [BB. 4]  CRP*Salmonella*ComplexPhagocytizeByNeutrophilSubRoutine [BB. 5]  CRP*Salmonella*ComplexPhagocytizeByMDMISubRoutine [BB. 6]  CRP*Salmonella*ComplexPhagocytizeByMDMIISubRoutine [BB. 7]  CRPBindToApoptoticKupfferCellSubRoutine [BB. 8]  CRPOpsonizedApoptoticKupfferCellPhagocytizeByKupfferCellSubRoutine [BB. 9]  CRPOpsonizedApoptoticKupfferCellPhagocytizeByMastCellSubRoutine [BB. 9]  CRPOpsonizedApoptoticKupfferCellPhagocytizeByNeutrophilSubRoutine [BB. 9]  CRPOpsonizedApoptoticKupfferCellPhagocytizeByMDMISubRoutine [BB. 9]  CRPOpsonizedApoptoticKupfferCellPhagocytizeByMDMIISubRoutine [BB. 9]  CRPBindToHepatocyteDebrisSubRoutine [BB. 10]  CRPOpsonizedHepatocyteDebrisPhagocytizeByKupfferCellSubRoutine [BB. 11]  CRPOpsonizedHepatocyteDebrisPhagocytizeByMastCellSubRoutine [BB. 11]  CRPOpsonizedHepatocyteDebrisPhagocytizeByNeutrophilSubRoutine [BB. 11]  CRPOpsonizedHepatocyteDebrisPhagocytizeByMDMISubRoutine [BB. 11]  CRPOpsonizedHepatocyteDebrisPhagocytizeByMDMIISubRoutine [BB. 11]  CRPBindToApoptoticNeutrophilSubRoutine [BB. 12]  CRPOpsonizedApoptoticNeutrophilPhagocytizeByKupfferCellSubRoutine [BB. 13]  CRPOpsonizedApoptoticNeutrophilPhagocytizeByMastCellSubRoutine [BB. 13]  CRPOpsonizedApoptoticNeutrophilPhagocytizeByNeutrophilSubRoutine [BB. 13]  CRPOpsonizedApoptoticNeutrophilPhagocytizeByMDMISubRoutine [BB. 13]  CRPOpsonizedApoptoticNeutrophilPhagocytizeByMDMIISubRoutine [BB. 13]  CRPBindToApoptoticMDMISubRoutine [BB. 14]  CRPOpsonizedApoptoticMDMIPhagocytizeByKupfferCellSubRoutine [BB. 15]  CRPOpsonizedApoptoticMDMIPhagocytizeByMastCellSubRoutine [BB. 15]  CRPOpsonizedApoptoticMDMIPhagocytizeByNeutrophilSubRoutine [BB. 15]  CRPOpsonizedApoptoticMDMIPhagocytizeByMDMISubRoutine [BB. 15]  CRPOpsonizedApoptoticMDMIPhagocytizeByMDMIISubRoutine [BB. 15]  CRPBindToApoptoticMDMIISubRoutine [BB. 16]  CRPOpsonizedApoptoticMDMIIPhagocytizeByKupfferCellSubRoutine [BB. 17]  CRPOpsonizedApoptoticMDMIIPhagocytizeByMastCellSubRoutine [BB. 17]  CRPOpsonizedApoptoticMDMIIPhagocytizeByNeutrophilSubRoutine [BB. 17]  CRPOpsonizedApoptoticMDMIIPhagocytizeByMDMISubRoutine [BB. 17]  CRPOpsonizedApoptoticMDMIIPhagocytizeByMDMIISubRoutine [BB. 17]  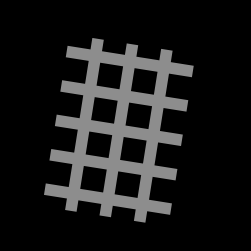CRPUndergoCatabolismByNatureSubRoutine [BB. 18] | 1. *Salmonella*  2. *ActivatedNeutrophil*  3. *KupfferCell*  4. *MDMI*  5. *MDMII*  6. *MastCell*  7*. HepatocyteDebris* |
| *NETs* (Neutrophil extracellular traps) |  | NETs are released by neutrophils [[11](#_ENREF_11)].  NETs interact with *Salmonella* [[10-12](#_ENREF_10)].  NET undergoes natural catabolism. | For BB. 1, see ActivatedNeutrophil Behavior(s). 12  NETTrapp*Salmonella*SubRoutine [BB. 2]  NETUndergoApoptosisByNatureSubRoutine [BB.3] | *Salmonella* |
| *SECs* (Sinusoid endothelial cells) | 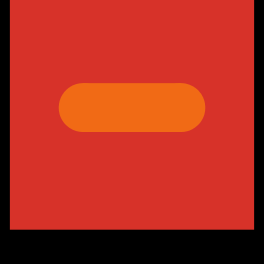 | SECs are infected by *Salmonella* [[5](#_ENREF_5)]. | For BB. 1, see *Salmonella* Behavior(s). 9 | *Salmonella* |
| *Signals* (Signal) | 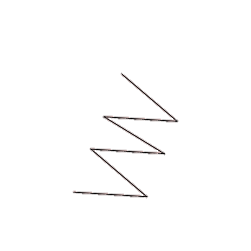 | Signals are sent by *Salmonella*, cytokines such as TNF-α and HMGB-1, or cells such as activated neutrophils and CD4^+^ T cells to recruit circulating neutrophil or circulating monocytes to the site of infection. | For BB. 1, see RestingNeutrophil Behavior(s). 2 and RestingMonocyte Behavior(s). 2 | *RestingNeutrophil*  *RestingMonocyte* |
| *AntiSignals* (Anti-signal) | 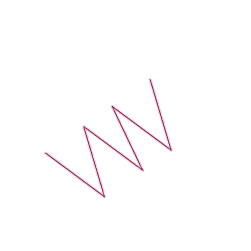 | Anti-Signals are sent by activated neutrophils bind to CRP [[86](#_ENREF_86)]. | For BB. 1, see ActivatedNeutrophil Behavior(s). 2 | *RestingNeutrophil*  *RestingMonocyte* |

**Model Assumption:**

1. We assume that resting neutrophils (circulating neutrophils) proliferate in the bone borrow and traffic to the vessel near the site of infection when the infection occurs. This assumption is based on observations in a study [[29](#_ENREF_29)] which showed massive neutrophil infiltration into peritoneum after 2 hrs of infection.
2. We assume MDMI and MDMII will conduct some similar functions such as phagocytosis of apoptotic T cells [[60](#_ENREF_60)]. Some observations failed to differentiate the behaviors of MDMI and MDMII [[60](#_ENREF_60)].
3. We assume that HMGB-1 contributes to activation of neutrophils and monocytes. This assumption is based on recent identified biological activities of HMGB-1 including upregulation of endothelial adhesion molecules during the infection [[32](#_ENREF_32)].
4. We assume that resting monocytes (circulating monocytes) develop in the bone borrow and are circulating in the vessel near the site of infection when the infection occurs. This assumption is based on observations in a study [[9](#_ENREF_9)] which showed massive monocyte infiltration following neutrophil infiltration.
5. We assume that activated neutrophils produce IL-10 after their interaction with *Salmonella* because it was observed that a rapid IL-10 production occurred during the early stages of sepsis [[41](#_ENREF_41)].
6. We assume the level of apoptotic activated neutrophils contributes to the activation process of resting monocytes (circulating monocytes) based on studies [[36](#_ENREF_36), [47](#_ENREF_47), [48](#_ENREF_48)] which showed activated macrophages phagocytize and ingest apoptotic neutrophils during infection.
7. We assume that TNF-α contributes to activation of neutrophils and monocytes. This assumption is based on recent identified biological evidence that TNF-α up-regulates endothelial adhesion molecules during the infection [[29](#_ENREF_29)] and improved endothelial adhesion to induce monocyte trafficking to hepatic sites of Gram-positive bacterial infection [[49](#_ENREF_49)]. Other studies supporting this assumption are cited [[45](#_ENREF_45), [50](#_ENREF_50), [51](#_ENREF_51)].
8. We assume that *Salmonella* contributes to activation of monocytes. This assumption is based on evidence showing that monocyte-derived-macrophages phagocytize Gram-negative bacteria [[52](#_ENREF_52)].
9. We assume resting monocytes (circulating monocytes) undergo a constant basal level of apoptosis (a constant decay rate per hr). This assumption is based on data showing that circulating monocytes are programmed to undergo apoptosis in the absence of stimulation [[53](#_ENREF_53)].
10. We assume TNF-α undergoes a degradation process. Experimental studies (various biological models) demonstrated TNF-α has a decay rate during infection [[64-66](#_ENREF_64)].
11. We assume HMGB-1 undergoes a degradation process. Experimental studies (various biological models) demonstrated HMGB-1 has a decay rate during infection [[69-71](#_ENREF_69)].
12. We assume IL-10 production inhibits the secretion of inflammatory cytokines (including TNF-α, HMGB-1 and IL-10) in our model. This assumption is based on biological evidence showing that IL-10 production inhibited the secretion of inflammatory cytokines [[72-75](#_ENREF_72)].
13. We assume IL-10 undergo a natural catabolism since experimental studies demonstrated IL-10 had a decay rate during infection [[76-78](#_ENREF_76)].
14. We assume each agent type has an equal chance of interacting with other agent types by following the agent rule.
15. We assume activated neutrophils can phagocytize multiple *Salmonella* (one ligand to multiple receptor mechanisms) at a time. However, Salmonella can’t interact with multiple activated neutrophils at a time. Similarly, MDMIs can phagocytize multiple *Salmonella* at a time. However, *Salmonella* can’t interact with multiple MDMIs at a time.
16. There is controversy about the replenishment of Kupffer Cells [[56](#_ENREF_56), [57](#_ENREF_57), [89](#_ENREF_89), [90](#_ENREF_90)]; however, we assume Kupffer Cells are only replaced by bone marrow-derived macrophages due to the fact only bone marrow-derived KCs engaged in inflammatory responses [[58](#_ENREF_58)].
17. We assume Kupffer Cells, mast cells, neutrophils, monocyte-derived-macrophage type I, and monocyte-derived-macrophage type II, interact with IL-10 since we assume IL-10 inhibits the production of pro-inflammatory cytokines in assumption 12.
18. We assume Kupffer Cells undergo apoptosis in the context of Kupffer cell turnover being observed [[57](#_ENREF_57), [58](#_ENREF_58)].
19. We assume one MDMII can phagocytize more than one apoptotic activated neutrophil as observed previously [[38](#_ENREF_38)].
20. We model T cell progenitors and B cell progenitors in the lymph node instead of thymus to generate mature CD4^+^ T cells, CD8^+^ T cells and B cells in our agent-based model.
21. We assume Kupffer Cells stop producing TNF-α by phagocytizing CRP-opsonized particles based on observations that synthetic CRP greatly decreased the production of TNF-α in a mouse model stimulated with *E.coli* [[20](#_ENREF_20)].
22. Mast cells were observed to present high-affinity FcγR receptor [[91](#_ENREF_91)]. We assume mast cells can phagocytize CRP-opsonized particles based on phagocytosis of CRP-opsonized particles proceeds through FcγRI in a mouse model [[92](#_ENREF_92), [93](#_ENREF_93)].
23. We determined NETs secretion from neutrophil degranulation is dependent on the quantity of secreted neutrophil elastase (NE) from neutrophil degranulation. This assumption is based on the observation that NETs is a complex of NE [[11](#_ENREF_11)]. Since we failed to directly find a quantitation of NETs secretion from neutrophils, we believe that using secreted NE level to represent secreted NETs could be a reasonable assumption.
24. The activation of macrophages is related to IFN-gamma released by T cells [[94-96](#_ENREF_94)]. Since we didn’t calibrate IFN-γ in our model, we simplify to calculate macrophage activation process using CD4^+^ T cell and CD8^+^ T cell count in our model instead of IFN-γ count.

Since we didn’t incorporate IL-6 in our model, we assume that CRP is released by hepatocytes [[97](#_ENREF_97)] in response to monocyte infiltration because data show that monocytes are the main producer for IL-6 [[98](#_ENREF_98)].

**References:**

1. Benacerraf B, Sebestyen MM, Schlossman S. A quantitative study of the kinetics of blood clearance of P32-labelled Escherichia coli and staphylococci by the reticulo-endothelial system. The Journal of Experimental Medicine. 1959;110(1):27-48.

2. Conlan JW, North RJ. Early pathogenesis of infection in the liver with the facultative intracellular bacteria Listeria monocytogenes, Francisella tularensis, and Salmonella typhimurium involves lysis of infected hepatocytes by leukocytes. Infection and Immunity. 1992;60(12):5164-5171.

3. Lindgren SW, Stojiljkovic I, Heffron F. Macrophage killing is an essential virulence mechanism of Salmonella typhimurium. Proceedings of the National Academy of Sciences. 1996;93(9):4197-4201.

4. Helaine S, Thompson JA, Watson KG, Liu M, Boyle C, Holden DW. Dynamics of intracellular bacterial replication at the single cell level. Proceedings of the National Academy of Sciences. 2010;107(8):3746-3751.

5. Nnalue N, Shnyra A, Hultenby K, Lindberg A. Salmonella choleraesuis and Salmonella typhimurium associated with liver cells after intravenous inoculation of rats are localized mainly in Kupffer cells and multiply intracellularly. Infection and Immunity. 1992;60(7):2758-2768.

6. Richter-Dahlfors A, Buchan AM, Finlay BB. Murine salmonellosis studied by confocal microscopy: Salmonella typhimurium resides intracellularly inside macrophages and exerts a cytotoxic effect on phagocytes in vivo. The Journal of Experimental Medicine. 1997;186(4):569-580.

7. Conlan JW. Neutrophils prevent extracellular colonization of the liver microvasculature by Salmonella typhimurium. Infection and Immunity. 1996;64(3):1043-1047.

8. Slauch JM. How does the oxidative burst of macrophages kill bacteria? Still an open question. Molecular Microbiology. 2011;80(3):580-583.

9. Liaskou E, Wilson DV, Oo YH. Innate immune cells in liver inflammation. Mediators of Inflammation. 2012;2012:949157. doi: 10.1155/2012/949157

10. Lacy P. Mechanisms of degranulation in neutrophils. Allergy Asthma Clin Immunol. 2006;2(3):98-108.

11. Papayannopoulos V, Metzler KD, Hakkim A, Zychlinsky A. Neutrophil elastase and myeloperoxidase regulate the formation of neutrophil extracellular traps. The Journal of Cell Biology. 2010;191(3):677-691.

12. Brinkmann V, Reichard U, Goosmann C, Fauler B, Uhlemann Y, Weiss DS, et al. Neutrophil extracellular traps kill bacteria. Science. 2004;303(5663):1532-1535.

13. Abraham SN, Malaviya R. Mast cells in infection and immunity. Infection and Immunity. 1997;65(9):3501-3508.

14. Dietrich N, Rohde M, Geffers R, Kröger A, Hauser H, Weiss S, et al. Mast cells elicit proinflammatory but not type I interferon responses upon activation of TLRs by bacteria. Proceedings of the National Academy of Sciences. 2010;107(19):8748-8753.

15. Black S, Kushner I, Samols D. C-reactive protein. Journal of Biological Chemistry. 2004;279(47):48487-48490.

16. Janeway CA, Travers P, Walport M, Shlomchik, MJ. Immunobiology: the immune system in health and disease. 6th ed. New York, Garland Science Publishing; 2005.

17. Kovach MA, Ballinger MN, Newstead MW, Zeng X, Bhan U, Yu FS, et al. Cathelicidin-related antimicrobial peptide is required for effective lung mucosal immunity in Gram-negative bacterial pneumonia. The Journal of Immunology. 2012;189(1):304-311.

18. Malhi H, Guicciardi ME, Gores GJ. Hepatocyte death: a clear and present danger. Physiological Reviews. 2010;90(3):1165-1194.

19. Du Clos TW. Function of C-reactive protein. Annals of Medicine. 2000;32(4):274-278.

20. Inatsu A, Kinoshita M, Nakashima H, Shimizu J, Saitoh D, Tamai S, et al. Novel mechanism of C-reactive protein for enhancing mouse liver innate immunity. Hepatology. 2009;49(6):2044-2054.

21. Ramaiah SK, Jaeschke H. Role of neutrophils in the pathogenesis of acute inflammatory liver injury. Toxicologic Pathology. 2007;35(6):757-766.

22. Jaeschke H, Bajt ML. Intracellular signaling mechanisms of acetaminophen-induced liver cell death. Toxicological Sciences. 2006;89(1):31-41.

23. Jaeschke H, Smith CW. Mechanisms of neutrophil-induced parenchymal cell injury. Journal of Leukocyte Biology. 1997;61(6):647-653.

24. Santos SAD, Andrade Júnior DRD, Andrade DRD. Tnf-a production and apoptosis in hepatocytes after listeria monocytogenes and salmonella typhimurium invasion. Revista do Instituto de Medicina Tropical de São Paulo. 2011;53(2):107-112.

25. Huang H, Nace GW, McDonald KA, Tai S, Klune JR, Rosborough BR, et al. Hepatocyte specific HMGB1 deletion worsens the injury in liver ischemia/reperfusion: a role for intracellular HMGB1 in cellular protection. Hepatology. 2014;59(5):1984-1997.

26. Guicciardi M, Gores G. Apoptosis: a mechanism of acute and chronic liver injury. Gut. 2005;54(7):1024-1033.

27. Neumann H, Kotter M, Franklin R. Debris clearance by microglia: an essential link between degeneration and regeneration. Brain. 2009;132(2):288-295.

28. Holt MP, Cheng L, Ju C. Identification and characterization of infiltrating macrophages in acetaminophen-induced liver injury. Journal of Leukocyte Biology. 2008;84(6):1410-1421.

29. Bian Z, Guo Y, Ha B, Zen K, Liu Y. Regulation of the inflammatory response: enhancing neutrophil infiltration under chronic inflammatory conditions. The Journal of Immunology. 2012;188(2):844-853.

30. Van Furth R, Diesselhoff-den Dulk MM, Mattie H. Quantitative study on the production and kinetics of mononuclear phagocytes during an acute inflammatory reaction. The Journal of Experimental Medicine. 1973;138(6):1314-1330.

31. Witthaut R, Farhood A, Smith CW, Jaeschke H. Complement and tumor necrosis factor-alpha contribute to Mac-1 (CD11b/CD18) up-regulation and systemic neutrophil activation during endotoxemia in vivo. Journal of Leukocyte Biology. 1994;55(1):105-111.

32. Wang H, Yang H, Tracey K. Extracellular role of HMGB1 in inflammation and sepsis. Journal of Internal Medicine. 2004;255(3):320-331.

33. Yang KK, Dorner BG, Merkel U, Ryffel B, Schütt C, Golenbock D, et al. Neutrophil influx in response to a peritoneal infection with Salmonella is delayed in lipopolysaccharide-binding protein or CD14-deficient mice. The Journal of Immunology. 2002;169(8):4475-4480.

34. Alberts B, Johnson A, Lewis J, Raff M, Roberts K, Walter P. Molecular biology of the cell. 4th ed. New York: Garland Science; 2002.

35. Brown SB, Savill J. Phagocytosis triggers macrophage release of Fas ligand and induces apoptosis of bystander leukocytes. The Journal of Immunology. 1999;162(1):480-485.

36. Gregory SH, Wing EJ. Neutrophil-Kupffer cell interaction: a critical component of host defenses to systemic bacterial infections. Journal of Leukocyte Biology. 2002;72(2):239-248.

37. Smith JA. Neutrophils, host defense, and inflammation: a double-edged sword. Journal of Leukocyte Biology. 1994;56(6):672-686.

38. Cox G, Crossley J, Xing Z. Macrophage engulfment of apoptotic neutrophils contributes to the resolution of acute pulmonary inflammation in vivo. American Journal of Respiratory Cell and Molecular Biology. 1995;12(2):232-237.

39. Savill J, Haslett C. Granulocyte clearance by apoptosis in the resolution of inflammation. Seminars in Cell Biology. 1995;6(6):385-393.

40. Aziz M, Jacob A, Yang WL, Matsuda A, Wang P. Current trends in inflammatory and immunomodulatory mediators in sepsis. Journal of Leukocyte Biology. 2013;93(3):329-342.

41. Kasten KR, Muenzer JT, Caldwell CC. Neutrophils are significant producers of IL-10 during sepsis. Biochemical and Biophysical Research Communications. 2010;393(1):28-31.

42. Kumar V, Sharma A. Neutrophils: cinderella of innate immune system. International Immunopharmacology. 2010;10(11):1325-1334.

43. Gao B. Hepatoprotective and anti-inflammatory cytokines in alcoholic liver disease. Journal of Gastroenterology and Hepatology. 2012;27(Suppl 2):89-93.

44. Gog JR, Murcia A, Osterman N, Restif O, McKinley TJ, Sheppard M, et al. Dynamics of Salmonella infection of macrophages at the single cell level. Journal of The Royal Society Interface. 2012;9(75):2696-2707.

45. Shi C, Pamer EG. Monocyte recruitment during infection and inflammation. Nature Reviews Immunology. 2011;11(11):762-774.

46. Schenkel AR, Mamdouh Z, Muller WA. Locomotion of monocytes on endothelium is a critical step during extravasation. Nature Immunology. 2004;5(4):393-400.

47. Savill J, Wyllie A, Henson J, Walport M, Henson P, Haslett C. Macrophage phagocytosis of aging neutrophils in inflammation. Programmed cell death in the neutrophil leads to its recognition by macrophages. Journal of Clinical Investigation. 1989;83(3):865-875.

48. Silva MT. Macrophage phagocytosis of neutrophils at inflammatory/infectious foci: a cooperative mechanism in the control of infection and infectious inflammation. Journal of Leukocyte Biology. 2011;89(5):675-683.

49. Shi C, Velázquez P, Hohl TM, Leiner I, Dustin ML, Pamer EG. Monocyte trafficking to hepatic sites of bacterial infection is chemokine independent and directed by focal intercellular adhesion molecule-1 expression. The Journal of Immunology. 2010;184(11):6266-6274.

50. Helk E, Bernin H, Ernst T, Ittrich H, Jacobs T, Heeren J, et al. TNFα-mediated liver destruction by Kupffer cells and Ly6Chi monocytes during Entamoeba histolytica infection. PLoS Pathogens. 2013;9(1):e1003096.

51. Indramohan M, Sieve AN, Break TJ, Berg RE. Inflammatory monocyte recruitment is regulated by interleukin-23 during systemic bacterial infection. Infection and Immunity. 2012;80(12):4099-4105.

52. Sindrilaru A, Peters T, Wieschalka S, Baican C, Baican A, Peter H, et al. An unrestrained proinflammatory M1 macrophage population induced by iron impairs wound healing in humans and mice. The Journal of Clinical Investigation. 2011;121(3):985-997.

53. Zhang Y, Morgan MJ, Chen K, Choksi S, Liu ZG. Induction of autophagy is essential for monocyte-macrophage differentiation. Blood. 2012;119(12):2895-2905.

54. Zen K, Masuda J, Ogata J. Monocyte-derived macrophages prime peripheral T cells to undergo apoptosis by cell-cell contact via ICAM-1/LFA-1-dependent mechanism. Immunobiology. 1996;195(3):323-333.

55. Ballou SP, Lozanski G. Induction of inflammatory cytokine release from cultured human monocytes by C-reactive protein. Cytokine. 1992;4(5):361-368.

56. Diesselhoff-den Dulk M, Crofton R, Van Furth R. Origin and kinetics of Kupffer cells during an acute inflammatory response. Immunology. 1979;37(1):7-14.

57. Crofton R, Diesselhoff-den Dulk MM, Furth Rv. The origin, kinetics, and characteristics of the Kupffer cells in the normal steady state. The Journal of Experimental Medicine. 1978;148(1):1-17.

58. Klein I, Cornejo JC, Polakos NK, John B, Wuensch SA, Topham DJ, et al. Kupffer cell heterogeneity: functional properties of bone marrow–derived and sessile hepatic macrophages. Blood. 2007;110(12):4077-4085.

59. Bellingan GJ, Caldwell H, Howie S, Dransfield I, Haslett C. In vivo fate of the inflammatory macrophage during the resolution of inflammation: inflammatory macrophages do not die locally, but emigrate to the draining lymph nodes. The Journal of Immunology. 1996;157(6):2577-2585.

60. Kasten KR, Tschöp J, Adediran SG, Hildeman DA, Caldwell CC. T cells are potent early mediators of the host response to sepsis. Shock. 2010;34(4):327-336.

61. Abe T, Arai T, Ogawa A, Hiromatsu T, Masuda A, Matsuguchi T, et al. Kupffer cell–derived interleukin 10 is responsible for impaired bacterial clearance in bile duct–ligated mice. Hepatology. 2004;40(2):414-423.

62. Bardadin KA, Scheuer PJ. Mast cells in acute hepatitis. The Journal of Pathology. 1986;149(4):315-325.

63. Yamashiro M, Kouda W, Kono N, Tsuneyama K, Matsui O, Nakanuma Y. Distribution of intrahepatic mast cells in various hepatobiliary disorders. Virchows Archiv. 1998;433(5):471-479.

64. Losser MR, Bernard C, Beaudeux JL, Pison C, Payen D. Glucose modulates hemodynamic, metabolic, and inflammatory responses to lipopolysaccharide in rabbits. Journal of Applied Physiology. 1997;83(5):1566-1574.

65. Xaus J, Comalada M, Valledor AF, Lloberas J, López-Soriano F, Argilés JM, et al. LPS induces apoptosis in macrophages mostly through the autocrine production of TNF-α. Blood. 2000;95(12):3823-3831.

66. Spencer NY, Zhou W, Li Q, Zhang Y, Luo M, Yan Z, et al. Hepatocytes produce TNF-α following hypoxia-reoxygenation and liver ischemia-reperfusion in a NADPH oxidase-and c-Src-dependent manner. American Journal of Physiology-Gastrointestinal and Liver Physiology. 2013;305(1):G84-G94.

67. Chen G, Li J, Ochani M, Rendon-Mitchell B, Qiang X, Susarla S, et al. Bacterial endotoxin stimulates macrophages to release HMGB1 partly through CD14-and TNF-dependent mechanisms. Journal of Leukocyte Biology. 2004;76(5):994-1001.

68. Tsung A, Klune JR, Zhang X, Jeyabalan G, Cao Z, Peng X, et al. HMGB1 release induced by liver ischemia involves Toll-like receptor 4–dependent reactive oxygen species production and calcium-mediated signaling. The Journal of Experimental Medicine. 2007;204(12):2913-2923.

69. Qin S, Wang H, Yuan R, Li H, Ochani M, Ochani K, et al. Role of HMGB1 in apoptosis-mediated sepsis lethality. The Journal of Experimental Medicine. 2006;203(7):1637-1642.

70. Gardella S, Andrei C, Ferrera D, Lotti LV, Torrisi MR, Bianchi ME, et al. The nuclear protein HMGB1 is secreted by monocytes via a non‐classical, vesicle‐mediated secretory pathway. EMBO Reports. 2002;3(10):995-1001.

71. Haveman J, Kobold AM, Tervaert JC, Van den Berg A, Tulleken J, Kallenberg C, et al. The central role of monocytes in the pathogenesis of sepsis: consequences for immunomonitoring and treatment. The Netherlands Journal of Medicine. 1999;55(3):132-141.

72. Geng Y, Gulbins E, Altman A, Lotz M. Monocyte deactivation by interleukin 10 via inhibition of tyrosine kinase activity and the Ras signaling pathway. Proceedings of the National Academy of Sciences. 1994;91(18):8602-8606.

73. Wang P, Wu P, Siegel MI, Egan RW, Billah MM. IL-10 inhibits transcription of cytokine genes in human peripheral blood mononuclear cells. The Journal of Immunology. 1994;153(2):811-816.

74. Wang P, Wu P, Siegel MI, Egan RW, Billah MM. Interleukin (IL)-10 inhibits nuclear factor B (NFB) activation in human monocytes IL-10 and IL-4 suppress cytokine synthesis by different mechanisms. Journal of Biological Chemistry. 1995;270(16):9558-9563.

75. Schottelius AJ, Mayo MW, Sartor RB, Baldwin AS. Interleukin-10 signaling blocks inhibitor of κB kinase activity and nuclear factor κB DNA binding. Journal of Biological Chemistry. 1999;274(45):31868-31874.

76. Yin S, Wang H, Park O, Wei W, Shen J, Gao B. Enhanced liver regeneration in IL-10–deficient mice after partial hepatectomy via stimulating inflammatory response and activating hepatocyte STAT3. The American Journal of Pathology. 2011;178(4):1614-1621.

77. Thompson K, Maltby J, Fallowfield J, McAulay M, Millward‐Sadler H, Sheron N. Interleukin‐10 expression and function in experimental murine liver inflammation and fibrosis. Hepatology. 1998;28(6):1597-1606.

78. Zhong J, Deaciuc IV, Burikhanov R, de Villiers WJ. Lipopolysaccharide-induced liver apoptosis is increased in interleukin-10 knockout mice. Biochimica et Biophysica Acta (BBA)-Molecular Basis of Disease. 2006;1762(4):468-477.

79. Chensue S, Terebuh P, Remick D, Scales W, Kunkel S. In vivo biologic and immunohistochemical analysis of interleukin-1 alpha, beta and tumor necrosis factor during experimental endotoxemia. Kinetics, Kupffer cell expression, and glucocorticoid effects. The American Journal of Pathology. 1991;138(2):395-402.

80. Hewett JA, Jean PA, Kunkel SL, Roth RA. Relationship between tumor necrosis factor-alpha and neutrophils in endotoxin-induced liver injury. American Journal of Physiology-Gastrointestinal and Liver Physiology. 1993;265(6):G1011-G1015.

81. Sugihara A, Tsujimura T, Fujita Y, Nakata Y, Terada N. Evaluation of role of mast cells in the development of liver fibrosis using mast cell-deficient rats and mice. Journal of Hepatology. 1999;30(5):859-867.

82. Abraham SN, John ALS. Mast cell-orchestrated immunity to pathogens. Nature Reviews Immunology. 2010;10(6):440-452.

83. Seeley EJ, Sutherland RE, Kim SS, Wolters PJ. Systemic mast cell degranulation increases mortality during polymicrobial septic peritonitis in mice. Journal of Leukocyte Biology. 2011;90(3):591-597.

84. Mekori YA, Metcalfe DD. Mast cell–T cell interactions. Journal of Allergy and Clinical Immunology. 1999;104(3):517-523.

85. Kindt TJ, Osborne BA, Goldsby RA. Kuby immunology. 6th ed. London: W.H. Freeman & Company; 2006.

86. Kew R, Hyers T, Webster R. Human C-reactive protein inhibits neutrophil chemotaxis in vitro: possible implications for the adult respiratory distress syndrome. The Journal of Laboratory and Clinical Medicine. 1990;115(3):339-345.

87. Zhong W, Zen Q, Tebo J, Schlottmann K, Coggeshall M, Mortensen RF. Effect of human C-reactive protein on chemokine and chemotactic factor-induced neutrophil chemotaxis and signaling. The Journal of Immunology. 1998;161(5):2533-2540.

88. Pepys MB, Hirschfield GM. C-reactive protein: a critical update. Journal of Clinical Investigation. 2003;111(12):1805-1812.

89. Naito M, Umeda S, Yamamoto T, Moriyama H, Umezu H, Hasegawa G, et al. Development, differentiation, and phenotypic heterogeneity of murine tissue macrophages. Journal of Leukocyte Biology. 1996;59(2):133-138.

90. Jenkins SJ, Ruckerl D, Cook PC, Jones LH, Finkelman FD, van Rooijen N, et al. Local macrophage proliferation, rather than recruitment from the blood, is a signature of TH2 inflammation. Science. 2011;332(6035):1284-1288.

91. Tkaczyk C, Okayama Y, Metcalfe DD, Gilfillan AM. Fcgamma receptors on mast cells: activatory and inhibitory regulation of mediator release. International Archives of Allergy and Immunology. 2004;133(3):305-315.

92. Mold C, Gresham HD, Du Clos TW. Serum amyloid P component and C-reactive protein mediate phagocytosis through murine FcγRs. The Journal of Immunology. 2001;166(2):1200-1205.

93. Mold C, Baca R, Du Clos TW. Serum amyloid P component and C-reactive protein opsonize apoptotic cells for phagocytosis through Fcγ receptors. Journal of Autoimmunity. 2002;19(3):147-154.

94. Kasahara T, Hooks J, Dougherty S, Oppenheim J. Interleukin 2-mediated immune interferon (IFN-gamma) production by human T cells and T cell subsets. The Journal of Immunology. 1983;130(4):1784-1789.

95. Mosser DM. The many faces of macrophage activation. Journal of Leukocyte Biology. 2003;73(2):209-212.

96. Held TK, Weihua X, Yuan L, Kalvakolanu DV, Cross AS. Gamma interferon augments macrophage activation by lipopolysaccharide by two distinct mechanisms, at the signal transduction level and via an autocrine mechanism involving tumor necrosis factor alpha and interleukin-1. Infection and Immunity. 1999;67(1):206-212.

97. Lau DC, Dhillon B, Yan H, Szmitko PE, Verma S. Adipokines: molecular links between obesity and atheroslcerosis. American Journal of Physiology-Heart and Circulatory Physiology. 2005;288(5):H2031-H2041.

98. Seshadri S, Kannan Y, Mitra S, Parker-Barnes J, Wewers MD. MAIL regulates human monocyte IL-6 production. The Journal of Immunology. 2009;183(8):5358-5368.
