# Supplementary material for: An Agent-Based Model of a Hepatic Inflammatory Response to Salmonella: A Computational Study under a Large Set of Experimental Data
Source: PLoS One. 2016 Aug 24;11(8):e0161131. doi: 10.1371/journal.pone.0161131 (PMC4996536; doi:10.1371/journal.pone.0161131)
Supplement: S3 Table — (DOCX) [file pone.0161131.s005.docx]

Table S3 Experimental data and value of system parameters in IMMABM

| *Agent Type* (Biological Indicator) | Experimental Data [ED] | System Parameter Value in IMMABM |
| --- | --- | --- |
| *Salmonella* (*Salmonella*) | *Salmonella* carrying capacity in Kupffer Cells is 4,558,000 bacteria in a rat model [[1](#_ENREF_1)].  *Salmonella* carrying capacity in hepatocytes is 817,000 bacteria in a rat model [[1](#_ENREF_1)].  *Salmonella* carrying capacity in liver endothelial cells is 67,000 bacteria in a rat model [[1](#_ENREF_1)].  *Salmonella* carrying capacity in macrophages is 4,558,000 bacteria in a rat model [an estimate based on *Salmonella* carrying capacity in Kupffer Cells].  *Salmonella* growth rate in macrophages is 0.9 fold/hr from 0 to 10hrs, and 10.9 fold/hr from 10 to 16 hrs, and 2.7 fold/hr from 16 to 25 hrs (measured in the spleen of mice) [[2](#_ENREF_2)].  *Salmonella* growth rate in epithelial cells is 0.32 fold/hr from 48 to 72 hrs (measured in the liver of rat) [[1](#_ENREF_1)].  *Salmonella* growth rate in Kupffer Cells is 0.42-1.04 fold/hr in rat model [[1](#_ENREF_1)].  *Salmonella* growth rate in hepatocytes is 0.05-0.26 fold/hr in rat model [[3](#_ENREF_3)].  22.79% of *E. coli* (Gram-negative bacteria) are killed by 1µM neutrophil elastase (NE) per hour [[4](#_ENREF_4)]. The rate at which *Salmonella* are trapped by NETs is not available [[5](#_ENREF_5)]. Since the NETs were a complex of Myeloperoxidase (MPO) and NE, we extrapolated the data above to estimate the rate of *Salmonella* killed by NETs.  There are controversial observations about whether *Salmonella* replicates within neutrophils. One study [[6](#_ENREF_6)] stated that *Salmonella* were rarely found inside neutrophils in control mice. However, others [[7](#_ENREF_7)] made an argument that neutrophils and macrophages were at the main site for *Salmonella* proliferation in the mouse because they found >95% of the *Salmonella* colocalized to the neutrophils and macrophages at any time point examined. Our model reflects that *Salmonella* replication rarely occurs within neutrophils because we failed to find any appropriate papers to support the concept that *Salmonella* replicates within neutrophils.  The Phagocytosis rate of *Salmonella* by Kupffer Cells is 90-95% of *Salmonella* (90-95% *Salmonella* are ingested by Kupffer Cells within first 6 hrs by phagocytosis) [[6](#_ENREF_6)].  Phagocytosis by macrophages stimulated with LPS takes approximately 2.5 hrs (90min for phagosome maturation + 60 min for engulfing process) [[8](#_ENREF_8)].  The maximum number of *Salmonella* phagocytized by one neutrophil is 17 [[9](#_ENREF_9)].  The maximum number of *Salmonella* phagocytized by one monocyte-derived macrophage is 30 [[9](#_ENREF_9)].  The maximum number of *Salmonella* phagocytized by one Kupffer Cell is 50 [[1](#_ENREF_1)].  The maximum number of *Salmonella* resides within one SEC is 3 [[1](#_ENREF_1)].  The maximum number of *Salmonella* resides within one hepatocyte is 15 [[10](#_ENREF_10)].  Phagocytosis of apoptotic cells by macrophages takes approximately 1 hr (on average), and the phagocytosis of necrotic cells by macrophages takes approximately 3 hrs (on average) [[11](#_ENREF_11)]. | *Salmonella*CarryCapacityInKupfferCell = 4558000  *Salmonella*CarryCapacityInHepatocyte =817000  *Salmonella*CarryCapacityInSECs = 67000  *Salmonella*CarryCapacityInMDMI = 4558000  *Salmonella*ReplicationRateInMDMIFrom0To10 = 0.9  *Salmonella*ReplicationRateInMDMIFrom10To16 = 10.9  *Salmonella*ReplicationRateInMDMIFrom16To25 = 2.7  *Salmonella*ReplicationRateInSECsPerHour = 0.32  *Salmonella*ReplicationRateInKupfferCellLowerLevelPerHour =0.42  *Salmonella*ReplicationRateInKupfferCellUpperLevelPerHour =1.04  *Salmonella*ReplicationRateInHepatocyteLowerLevelPerHour = 0.05  *Salmonella*ReplicationRateInHepatocyteUpperLevelPerHour = 0.26  percentageOf*Salmonella*BeingTrappedByNETPerHour = 0.2279  percentageOf*Salmonella*PhagocytizeByKupfferCellLowerLevel = 0.90  percentageOf*Salmonella*PhagocytizeByKupfferCellUpperLevel = 0.95  timeOf*Salmonella*KillByKupfferCell = 6  timeOf*Salmonella*DieInduceByNeutrophil = 2  timeOf*Salmonella*DieInduceByMDMI = 2  maximumNumberOf*Salmonella*BeingKilledByNeutrophil = 17  maximumNumberOf*Salmonella*BeingKilledByMDMI = 30  maximumNumberOf*Salmonella*BeingKilledByKupfferCell = 50  maximumNumberOf*Salmonella*ResideWithinSECs = 3  maximumNumberOf*Salmonella*ResideWithinHepatocyte = 15  timeOf*Salmonella*CRPComplexDie = 1 |
| *KupfferCell* (Kupffer cell) | Kupffer Cell machinery is disrupted and cells die 6-14 hrs after *Salmonella* infection [[12](#_ENREF_12)].  By direct cell contact with target cells, T cells could deliver a cytotoxic signal that induces apoptosis in target cells in approximately 4 hrs (on average) [[13](#_ENREF_13)].  Rate of TNF-α secreted by Kupffer Cells in Sham-operated mice upon injection of *E.coli* is 2.16×10^-4^-2.30×10^-4^pg/Kupffer Cell/hr from 0-3 hrs [[14](#_ENREF_14)].  Rate of TNF-α secreted by Kupffer Cells in Sham-operated mice upon injection of *E.coli* is 4.88×10^-5^-8.36×10^-5^ pg/Kupffer Cell/hr from 3-6 hrs [[14](#_ENREF_14)].  Rate of TNF-α secreted by Kupffer Cells in Sham-operated mice upon injection of *E.coli* is 2.09×10^-5^ pg/Kupffer Cell/hr from 6-10 hrs [[14](#_ENREF_14)].  The average amount of TNF-α damage one hepatocyte is 2.82×10^-5^pg in a mouse model [[15](#_ENREF_15)].  Rate of IL-10 secretion by Kupffer Cells is 6.15×10^-4^ -7.38×10^-4^pg/Kupffer Cell/hr in a mouse model injected with *E.coli* [[14](#_ENREF_14)].  The binding rate of IL-10 to one cell is approximately 1.23×10^-5^ pg/cell [[16](#_ENREF_16)].  Phagocytosis of apoptotic cells by macrophages takes approximately 1 hr (on average), and the phagocytosis of necrotic cells by macrophages takes approximately 3 hrs (on average) [[11](#_ENREF_11)]. | lowerTimeOfKupfferCellKillBy*Salmonella* = 6  upperTimeOfKupfferCellKillBy*Salmonella* = 14  timeOfKupfferCellWhoPhagocytize*Salmonella*DieByInteractWithCD8TCell = 4  maximumReleaseRateOfTNFAlphaFromKupfferCellPerHourFrom0To3 = 2.30E-4  maximumReleaseRateOfTNFAlphaFromKupfferCellPerHourFrom3To6 = 8.36E-5  maximumReleaseRateOfTNFAlphaFromKupfferCellPerHourFrom6To10 = 2.09×10^-5^  amountOfTNFAlphaBeingRepresentedByOneAgent = 2.82E-5  maximumReleaseRateOfIL10ByKupfferCellPerHour = 7.38E-4  amountOfIL10BeingRepresentedByOneAgent = 1.23E-5  timeOfApoptoticKupfferCellCRPComplexDie = 1 |
| *RestingNeutrophil* (Circulating neutrophil) | The neutrophil influx into blood vessels from bone marrow stores starts at approximately 1 hr [[17](#_ENREF_17)].  Neutrophil influx rates from bone marrow to blood vessel in a human model [[17](#_ENREF_17)].  Neutrophil influx rate from bone marrow into blood vessel is 14 fold /hr over a period of 1-1.5 hrs.  Neutrophil influx rate from bone marrow into blood vessel is 0.39 fold/hr over a period of 1.5-4 hrs.  Neutrophil influx rate from bone marrow into blood vessel is 0.125 fold/hr over a period of 4-6 hrs.  Neutrophil counts in blood vessels in a mouse model anesthetized with ketamine and xylazine was 6.9×10^3^ cells/ul [[18](#_ENREF_18), [19](#_ENREF_19)]  Circulating neutrophils undergo apoptosis at a rate of 0.05-0.092 fold/hr (half-life is 6-10 hrs) [[20](#_ENREF_20), [21](#_ENREF_21)] | timeOfNeutrophilStartToInfluxIntoLiverSinusoid = 1  influxRateOfRestingNeutrophilToLiverSinusoidFrom0To2 = 14  influxRateOfRestingNeutrophilToLiverSinusoidFrom2To4 = 0.39  influxRateOfRestingNeutrophilToLiverSinusoidFrom4ToEnd = 0.125  restingNeutrophilCarryCapacityInLiverSinusoid = 6900  rateOfRestingNeutrophilUndergoAgingPerHourLowerLevel = 0.05  rateOfRestingNeutrophilUndergoAgingPerHourLowerLevel = 0.095 |
| *RestingMonocyte* (Circulating monocyte) | Monocytes infiltration into blood vessels begins at approximately 2 hrs after infection in a mouse lung model infected with *Escherichia coli* [[22](#_ENREF_22)].  Influx rate of monocytes into blood vessels in a mouse lung model infected with *Escherichia coli* is 1-1.75 fold/hr [[22](#_ENREF_22)].  Monocyte carrying capacity in blood vessel was 1.4×10^3^ cells/ul [[18](#_ENREF_18), [19](#_ENREF_19)].  The influx rate of Ly6C^high^ monocytes into the liver in a rat infected with *L. monocytogenes* (Gram-positive bacteria) is 0.25-4.82 fold/hr [[23](#_ENREF_23)].  Circulating monocytes undergo apoptosis at a rate of 6.90×10^-3^ -2.10×10^-2^ fold/hr (this is based on data showing circulating monocytes have a half-life about one to three days) [[24](#_ENREF_24)]. | timeOfRestingMonocyteInfluxIntoLiverSinusoid = 2  influxRateOfRestingMonocyteToLiverSinusoidPerHourLowerLevel = 1  influxRateOfRestingMonocyteToLiverSinusoidPerHourUpperLevel = 1.75  restingMonocyteCarryCapacityInLiverSinusoid = 1400  activationRateOfRestingMonocytePerHourLowerLevel = 0.25  activationRateOfRestingMonocytePerHourUpperLevel = 4.82  apoptoticRateOfRestingMonocyteByAgingPerHourLowerLevel = 0.0069  apoptoticRateOfRestingMonocyteByAgingPerHourUpperLevel = 0.021 |
| *Hepatocyte* (Hepatocyte) | Hepatocyte replication rates after partial hepatectomy [[25](#_ENREF_25)].  Hepatocyte replication rate is 2.65×10^-3^ - 3.17×10^-3^ fold/hr over a period of 0-98 hrs.  Hepatocyte replication rate is 4.08×10^-3^ -6.80×10^-3^ fold/hr over a period of 98-135 hrs.  Hepatocyte replication rate is 1.32×10^-3^ -3.95×10^-3^ fold/hr over a period of 135-173 hrs.  Hepatocyte replication rate is 4.08×10^-3^ -5.27×10^-3^ fold/hr over a period of 173-247 hrs.  Hepatocyte replication rate is 2.12×10^-3^ -2.65×10^-3^ fold/hr over a period of 247-336 hrs.  Hepatocyte are infected by *Salmonella* at an infected rate is 0.003 *Salmonella*/hepatocyte/hr [[10](#_ENREF_10)].  The time from initiation of apoptosis by hepatocytes to completion ranges from 2-3 hrs. These data were inferred from a general model that didn’t specify apoptosis rates for various organs [[26](#_ENREF_26)].  Mouse circulating CRP level increases from 0.3mg/ml to 6 mg/ml by 24 hrs after endotoxin injection [[27](#_ENREF_27)]. The rate of CRP released from hepatocytes is approximately 2×10^-7^ µg/hepatocyte/hr [[19](#_ENREF_19), [27](#_ENREF_27)]. This is the only paper that we could find that measured circulating CRP levels in mice. Most of CRP levels are measured in human models.  The binding rate of CRP to one phagocytic cell is approximately 1.25×10^-5^µg/cell [[28](#_ENREF_28)].  Rate of TNF-α secreted by hepatocytes infected with *Salmonella* is 7.14×10^-5^ -9.18×10^-5^ pg/hepatocyte/hr [[3](#_ENREF_3)].  Rate of HMGB-1 secretion by apoptotic hepatocytes in Sham-operated mice is approximately 6.25×10^-5^pg/hepatocyte/hr [[19](#_ENREF_19), [29](#_ENREF_29)]. | hepatocyteReplicationRatePerHourFrom0To98LowerLevel = 2.65E-3  hepatocyteReplicationRatePerHourFrom0To98UpperLevel = 3.17E-3  hepatocyteReplicationRatePerHourFrom98To135LowerLevel = 4.08E-3  hepatocyteReplicationRatePerHourFrom98To135UpperLevel = 6.8E-3  hepatocyteReplicationRatePerHourFrom135To173LowerLevel = 1.32E-3  hepatocyteReplicationRatePerHourFrom135To173UpperLevel = 3.95E-3  hepatocyteReplicationRatePerHourFrom173To247LowerLevel = 4.08E-3  hepatocyteReplicationRatePerHourFrom173To247UpperLevel = 5.27E-3  hepatocyteReplicationRatePerHourFrom247ToEndLowerLevel = 2.12E-3  hepatocyteReplicationRatePerHourFrom247ToEndUpperLevel = 2.65E-3  rateOfHepatocyteBeingInfectedBy*Salmonella*PerHour = 0.003  timeOfHepatocyteBecomeDebrisLowerLevel = 2  timeOfHepatocyteBecomeDebrisUpperLevel = 3  maximumReleaseRateOfCRPByHepatocytePerHour = 2.00E-7  amountOfCRPBeingRepresentedByOneAgent = 1.25E-5  maximumReleaseRateOfTNFAlphaByApoptoticHepatocytePerHour = 9.18E-5  maximumReleaseRateOfHMGB1ByApoptoticHepatocytePerHour = 6.25E-5 |
| *HepatocyteDebris* (Hepatocyte Debris) | Phagocytosis of apoptotic cells by macrophages takes approximately 1 hr (on average), and the phagocytosis of necrotic cells by macrophages takes approximately 3 hrs (on average) [[11](#_ENREF_11)]. | timeOfHepatocyteDebrisCRPComplexDie = 1 |
| *ActivatedNeutrophil* (Activated neutrophil, mostly focus on neutrophils at the site of infection) | Influx rate of circulating neutrophils in a rat model of acute pulmonary inflammation stimulated with LPS of *Escherichia coli* is 0.21-0.46 fold/hr [[30](#_ENREF_30)]. Another study [[31](#_ENREF_31)] showed that activated neutrophils infiltrate into the site of infection at a rate of 0.09-0.16 fold/hr (influx rate is measured as the influx rate of neutrophils into the peritoneum).  Massive neutrophils infiltration into the peritoneum occurred after 2 hrs [[32](#_ENREF_32)]. Activated neutrophils infiltrate into the site of infection by 2 hrs after infection in mice infected in the peritoneum with *Salmonella* [[31](#_ENREF_31)].  Killing rate of *Escherichia coli* (*E. coli* is recognized as a Gram-negative bacteria) by neutrophils is 2.94-12.94 *E.coli*/neutrophil/hr [[9](#_ENREF_9)].  It takes 5-20 neutrophils to injury one hepatocyte by cell-cell contact [[33](#_ENREF_33), [34](#_ENREF_34)]. Also, activated neutrophils accelerate the killing process of apoptotic hepatocytes [[35](#_ENREF_35)].  30% of circulating activated neutrophils are phagocytized by Kupffer Cells in a mouse model by 6 hrs after LPS injection [[36](#_ENREF_36)].  Activated neutrophils undergo apoptosis at a rate of 0.098 fold/hr (assuming a constant decrease)(the apoptosis was based on mice with meningitis) [[21](#_ENREF_21)].  By direct cell contact with target cells, T cells could deliver a cytotoxic signal that induces apoptosis in target cells in approximately 4 hrs (on average) [[13](#_ENREF_13)].  Rate of IL-10 secretion by neutrophils in spetic mice upon CLP is 8.44×10^-5^-1.03×10^-4^ pg/neutrophil/hr [[37](#_ENREF_37)].  Rate of TNF-α secretion by neutrophils in a mouse model injected with *E.coli* LPS is 0.19-0.27pg/neutrophil/hr over a period of 0-1 hr [[38-42](#_ENREF_38)].  Rate of TNF-α secretion by neutrophils in a mouse model injected with *E.coli* LPS is 1.47-2.00 pg/neutrophil/hr over a period of 1-1.5 hrs [[38-42](#_ENREF_38)].  Phagocytosis of apoptotic cells by macrophages takes approximately 1 hr (on average), and the phagocytosis of necrotic cells by macrophages takes approximately 3 hrs (on average) [[11](#_ENREF_11)].  Phagocytosis by macrophages stimulated with LPS takes approximately 2.5 hrs (90min for phagosome maturation + 60 min for engulfing process) [[8](#_ENREF_8)].  Phagocytosis of apoptotic cells by macrophages takes approximately 1 hr (on average), and the phagocytosis of necrotic cells by macrophages takes approximately 3 hrs (on average) [[11](#_ENREF_11)]. | activationRateOfRestingNeutrophilPerHourLowerLevel = 0.21  activationRateOfRestingNeutrophilPerHourUpperLevel = 0.46  timeOfNeutrophilStartToInfluxIntoSiteOfInfection = 2  phagocytizeRateOf*Salmonella*ByActivatedNeutrophilPerHourLowerLevel = 2.94  phagocytizeRateOf*Salmonella*ByActivatedNeutrophilPerHourUpperLevel = 12.94  killingRateOfApoptoticHepatocyteByNeutrophilPerHourLowerLevel = 0.05  killingRateOfApoptoticHepatocyteByNeutrophilPerHourUpperLevel = 0.2  percentageOfNeutrophilBeingKilledByKupfferCell = 0.05  apoptoticRateOfActivatedNeutrophilByNaturePerHour = 0.098  timeOfActivatedNeutrophilWhoPhagocytize*Salmonella*DieByInteractWithCD8TCell = 4  maximumReleaseRateOfIL10ByNeutrophilPerHour = 1.03E-4  maximumReleaseRateOfTNFAlphaByNeutrophilFrom0To1 = 0.27  maximumReleaseRateOfTNFAlphaByNeutrophilFrom1ToEnd = 2  timeOfApoptoticNeutrophilCRPComplexDie = 1  timeOfNeutrophilKillByKupfferCell = 2  timeOfApoptoticNeutrophilKillByMDMII = 1 |
| *NET* (NET) | Rate of NE secretion by neutrophils was 3.2×10^-7^ µM /neutrophil during the first 1 hr, and the stopped [[43](#_ENREF_43)]. | amountOfNETBeingRepresentedByOneAgent = 3.2E-7 |
| *MDMI* (monocyte-Derived-Macrophage Type I) | Ly6C^high^ monocytes are recruited to the liver in a rat model by 6 hrs after infection with *L. monocytogenes* (Gram-positive bacteria) [[23](#_ENREF_23)]. Also, monocyte infiltration to peritoneum was detected to increase at 6 hrs after infection in a mouse model with Zymosan-induced peritonitis [[32](#_ENREF_32)].  Kupffer Cells are replenished hourly by 0.63 - 0.79% of monocyte-derived-macrophage type I or monocyte-derived-macrophage type II upon zymosan injection in the mouse model [[44](#_ENREF_44)].  By direct cell contact with target cells, T cells could deliver a cytotoxic signal that induces apoptosis in target cells in approximately 4 hrs (on average) [[13](#_ENREF_13)].  Rate of TNF-α secretion by peritoneal macrophages in Sham-operated mice upon injection of *E.coli* is 1.70×10^-4^ pg/ peritoneal macrophage/hr over a period of 0-3 hrs [[14](#_ENREF_14)].  Rate of IL-10 secretion by peritoneal macrophages in Sham-operated mice upon injection of *E.coli* is 2.02×10^-5^ pg/ peritoneal macrophage /hr over a period of 0-3 hrs [[14](#_ENREF_14)].  Phagocytosis of apoptotic cells by macrophages takes approximately 1 hr (on average), and the phagocytosis of necrotic cells by macrophages takes approximately 3 hrs (on average) [[11](#_ENREF_11)]. | timeOfMonocyteInfluxIntoSiteOfInfection = 6  rateOfMDMITransformToKupfferCellLowerLevel = 0.0063  rateOfMDMITransformToKupfferCellUpperLevel = 0.0079  timeOfMDMIWhoPhagocytize*Salmonella*DieByInteractWithCD8TCell = 4  maximumReleaseRateOfTNFAlphaByMDMIPerHour = 1.7E-4  maximumReleaseRateOfIL10ByMDMIPerHour = 2.02E-5  timeOfApoptoticMDMICRPComplexDie = 1 |
| *MDMII* (monocyte-Derived-Macrophage Type II) | Kupffer Cells are hourly replenished by 0.63% - 0.79% of monocyte-derived-macrophage type I or monocyte-derived-macrophage type II upon zymosan injection in mice model [[44](#_ENREF_44)].  Rate of IL-10 secretion by peritoneal macrophages in Sham-operated mice upon injection of *E.coli* is 2.02×10^-5^ pg/ peritoneal macrophage/hr over a period of 0-3 hrs [[14](#_ENREF_14)].  Rate of HMGB-1 secretion by peritoneal macrophages in a rat model is 9.38×10^-3^-3.8×10^-2^pg/peritoneal macrophage/hr over a period of 8-12 hrs [[45](#_ENREF_45), [46](#_ENREF_46)].  Rate of HMGB-1 secretion by peritoneal macrophages in a rat model is 1.03×10^-1^-1.69×10^-1^pg/peritoneal macrophage/hr over a period of 12-16 hrs [[45](#_ENREF_45), [46](#_ENREF_46)].  Rate of HMGB-1 secretion by peritoneal macrophages in a rat model is 2.72×10^-1^-4.97×10^-1^pg/peritoneal macrophage/hr over a period of 16-24 hrs [[45](#_ENREF_45), [46](#_ENREF_46)].  Phagocytosis of apoptotic cells by macrophages takes approximately 1 hr (on average), and the phagocytosis of necrotic cells by macrophages takes approximately 3 hrs (on average) [[11](#_ENREF_11)]. | rateOfMDMIITransformToKupfferCellLowerLevel = 0.0063  rateOfMDMIITransformToKupfferCellUpperLevel = 0.0079  maximumReleaseRateOfIL10ByMDMIIPerHour = 2.02E-5  maximumReleaseRateOfHMGB1ByMDMIIFrom8To12 = 3.8E-2  maximumReleaseRateOfHMGB1ByMDMIIFrom12To16 = 1.69E-1  maximumReleaseRateOfHMGB1ByMDMIIFrom16ToEnd = 4.97E-1  timeOfApoptoticMDMIICRPComplexDie = 1 |
| *MastCell* (Mast cell) | Mast cells undergo self-renewal after CCI_4_ injection in a rat model, the proliferation rate is 9.45×10^-4^-3.10×10^-3^ /hr [[47](#_ENREF_47)].  Rate of TNF-α secretion by mast cells in a mouse model injected with CLP ranges from 1.33×10^-7^ to 1.52×10^-7^ pg/mast cell/hr [[48](#_ENREF_48)].  Rate of TNF-α secretion by peritoneal mast cells stimulated with antigen (a collection of soluble excretory and secretory products of adult *N. brasiliensis*) was 1.48×10^-4^-1.76×10^-4^ pg/mast cell/hr [[49](#_ENREF_49)]. We use these rates to estimate the rates of TNF-α secretion by mast cells during degranulation in IMMAB.  Mast cells release histamine during systemic degranulation induced by polymicrobial septic peritonitis in a mouse model. The rate of histamine release by mast cells is 0.12-0.18 pg /mast cell/hr [[19](#_ENREF_19), [50](#_ENREF_50)]. | proliferateRateOfMastCellPerHourLowerLevel = 9.45E-4  proliferateRateOfMastCellPerHourUpperLevel = 0.0031  maximumReleaseRateOfTNFAlphaByMastCellPerHour = 1.52E-7  maximumReleaseRateOfTNFAlphaByMastCellIfInteractWithAntibody*Salmonella*ComplexPerHour = 1.76E-4  maximumReleaseRateOfHistamineByMastCellIfInteractWithAntibody*Salmonella*ComplexPerHour = 0.18 |
| *CD4TCell* (CD4 T cell) | The activation rate of CD4 T cells in the spleen of mice injected with *E. coli* ranges from 1.24×10^-3^ to 2.75×10^-2^ fold/hr [[51](#_ENREF_51)].  The CD4 T cells carrying capacity is approximately 27.4×10^6^ cells in the first 7 days after infection [[51](#_ENREF_51)].  Rate of IL-10 secretion by Th2 cells stimulated with IL-4 is 8.33×10^-7^-9.69×10^-7^pg/Th2 cell/hr [[52](#_ENREF_52)].  Rate of TNF-α secretion by T cells in a mouse model infected with *E. coli* was 6.94×10^-7^pg/T cell/hr [[51](#_ENREF_51)]. | influxRateOfCD4TCellToLiverSinusoidPerHourLowerLevel = 1.24E-3  influxRateOfCD4TCellToLiverSinusoidPerHourUpperLevel = 2.75E-2  CD4TCellCarryCapacityInLiverSinusoid = 27.4E6  maximumReleaseRateOfIL10ByCD4TCellPerHour = 9.69E-7  maximumReleaseRateOfTNFAlphaByCD4TCellPerHour = 6.94E-7 |
| *CD8TCell* (CD8 T cell) | The activation rate of CD8 T cells in the spleen of mice injected with *E. coli* is approximately 6.25×10^-2^ fold/hr [[51](#_ENREF_51)].  The CD8 T cells carrying capacity is approximately 5×10^6^ cells in the first 7 days after infection [[51](#_ENREF_51)]. | InfluxRateOfCD8TCellToLiverSinusoidPerHour = 6.25E-2  CD8TCellCarryCapacityInLiverSinusoid = 5000000 |
| *BCell* (B cell) | The activation rate of B cells in the spleen of mice injected with *E. coli* ranges from 4.30 ×10^-4^ to 2.40×10^-2^ fold/hr [[51](#_ENREF_51)].  The B cells carrying capacity is approximately 28.6×10^6^ in the first 7 days after infection [[51](#_ENREF_51)]. | influxRateOfBCellToLiverSinusoidPerHourLowerLevel = 4.3E-4  influxRateOfBCellToLiverSinusoidPerHourUpperLevel = 2.4E-2  BCellCarryCapacityInLiverSinusoid = 28600000 |
| *TNFAlpha* (Tumor necrosis factor alpha) | Kupffer Cells secrete TNF-a  Rate of TNF-α secreted by Kupffer Cells in Sham-operated mice upon injection of *E.coli* is 2.16×10^-4^-2.30×10^-4^pg/Kupffer Cell/hr from 0-3 hrs [[14](#_ENREF_14)].  Rate of TNF-α secreted by Kupffer Cells in Sham-operated mice upon injection of *E.coli* is 4.88×10^-5^-8.36×10^-5^ pg/Kupffer Cell/hr from 3-6 hrs [[14](#_ENREF_14)].  Rate of TNF-α secreted by Kupffer Cells in Sham-operated mice upon injection of *E.coli* is 2.09×10^-5^ pg/Kupffer Cell/hr from 6-10 hrs [[14](#_ENREF_14)].  Rate of TNF-α secretion by neutrophils in a mouse model injected with *E.coli* LPS is 0.19-0.27pg/neutrophil/hr over a period of 0-1 hr [[38-42](#_ENREF_38)].  Rate of TNF-α secretion by neutrophils in a mouse model injected with *E.coli* LPS is 1.47-2.00 pg/neutrophil/hr over a period of 1-1.5 hrs [[38-42](#_ENREF_38)].  Rate of TNF-α secretion by peritoneal macrophages in Sham-operated mice upon injection of *E.coli* is 1.70×10^-4^ pg/ peritoneal macrophage/hr over a period of 0-3 hrs [[14](#_ENREF_14)].  Rate of TNF-α secretion by mast cells in a mouse model injected with CLP ranges from 1.33×10^-7^ to 1.52×10^-7^ pg/mast cell/hr [[48](#_ENREF_48)].  Rate of TNF-α secretion by peritoneal mast cells stimulated with antigen (a collection of soluble excretory and secretory products of adult *N. brasiliensis*) was 1.48×10^-4^-1.76×10^-4^ pg/mast cell/hr [[49](#_ENREF_49)]. We use these rates to estimate the rates of TNF-α secretion by mast cells during degranulation in IMMABM.  Rate of TNF-α secretion by T cells in a mouse model infected with *E. coli* was 6.94×10^-7^pg/T cell/hr [[51](#_ENREF_51)]. | maximumReleaseRateOfTNFAlphaFromKupfferCellPerHourFrom0To3 = 2.30E-4  maximumReleaseRateOfTNFAlphaFromKupfferCellPerHourFrom3To6 = 8.36E-5  maximumReleaseRateOfTNFAlphaFromKupfferCellPerHourFrom6To10 = 2.09×10^-5^  maximumReleaseRateOfTNFAlphaByNeutrophilFrom0To1 = 0.27  maximumReleaseRateOfTNFAlphaByNeutrophilFrom1ToEnd = 2  maximumReleaseRateOfTNFAlphaByMDMIPerHour = 1.7E-4  maximumReleaseRateOfTNFAlphaByMastCellPerHour = 1.52E-7  maximumReleaseRateOfTNFAlphaByMastCellIfInteractWithAntibody*Salmonella*ComplexPerHour = 1.76E-4  maximumReleaseRateOfTNFAlphaByCD4TCellPerHour = 6.94E-7 |
| *HMGB1* (High-Mobility Group Box 1) | Rate of HMGB-1 secretion by apoptotic hepatocytes in Sham-operated mice is approximately 6.25×10^-5^pg/hepatocyte/hr [[19](#_ENREF_19), [29](#_ENREF_29)].  Rate of HMGB-1 secretion by peritoneal macrophages in a rat model is 9.38×10^-3^-3.8×10^-2^pg/peritoneal macrophage/hr over a period of 8-12 hrs [[45](#_ENREF_45), [46](#_ENREF_46)].  Rate of HMGB-1 secretion by peritoneal macrophages in a rat model is 1.03×10^-1^-1.69×10^-1^pg/peritoneal macrophage/hr over a period of 12-16 hrs [[45](#_ENREF_45), [46](#_ENREF_46)].  Rate of HMGB-1 secretion by peritoneal macrophages in a rat model is 2.72×10^-1^-4.97×10^-1^pg/peritoneal macrophage/hr over a period of 16-24 hrs [[45](#_ENREF_45), [46](#_ENREF_46)]. | maximumReleaseRateOfHMGB1ByApoptoticHepatocytePerHour = 6.25E-5  maximumReleaseRateOfHMGB1ByMDMIIFrom8To12 = 3.8E-2  maximumReleaseRateOfHMGB1ByMDMIIFrom12To16 = 1.69E-1  maximumReleaseRateOfHMGB1ByMDMIIFrom16ToEnd = 4.97E-1 |
| *IL10* (Interleukin 10) | Rate of IL-10 secretion by Kupffer Cells is 6.15×10^-4^ -7.38×10^-4^pg/Kupffer Cell/hr in a mouse model injected with *E.coli* [[14](#_ENREF_14)].  Rate of IL-10 secretion by neutrophils in spetic mice upon CLP is 8.44×10^-5^-1.03×10^-4^ pg/neutrophil/hr [[37](#_ENREF_37)].  Rate of IL-10 secretion by peritoneal macrophages in Sham-operated mice upon injection of *E.coli* is 2.02×10^-5^ pg/ peritoneal macrophage /hr over a period of 0-3 hrs [[14](#_ENREF_14)].  Rate of IL-10 secretion by peritoneal macrophages in Sham-operated mice upon injection of *E.coli* is 2.02×10^-5^ pg/ peritoneal macrophage/hr over a period of 0-3 hrs [[14](#_ENREF_14)].  Rate of IL-10 secretion by Th2 cells stimulated with IL-4 is 8.33×10^-7^-9.69×10^-7^pg/Th2 cell/hr [[52](#_ENREF_52)]. | maximumReleaseRateOfIL10ByKupfferCellPerHour = 7.38E-4  maximumReleaseRateOfIL10ByNeutrophilPerHour = 1.03E-4  maximumReleaseRateOfIL10ByMDMIPerHour = 2.02E-5  maximumReleaseRateOfIL10ByMDMIIPerHour = 2.02E-5  maximumReleaseRateOfIL10ByCD4TCellPerHour = 9.69E-7 |
| *CRP* (C-reactive protein) | CRP undergoes degradation at an estimated rate of 0.26 fold/hr (Plasma half-life of CRP is about 19 hrs and is constant under all conditions of health and disease, this data was extrapolated from a human model) [[53](#_ENREF_53)]. | rateOfCRPUndergoApoptosisByNaturePerHour = 0.26 |
| *Antibody* (antibody) | The antibody production amount by one B cell in a human model infected by *Salmonella* is 4.88×10^-4^-2.81×10^-3^pg/B cell/hr [[54](#_ENREF_54)].  The binding amount of antibody to one *Salmonella* is 5.31 pg/*Salmonella* [[17](#_ENREF_17), [55](#_ENREF_55)]. | maximumReleaseRateOfAntibodyByBCellLowerLevelPerHour = 4.88E-4  maximumReleaseRateOfAntibodyByBCellUpperLevelPerHour = 0.00281  amountOfAntibodyBeingRepresentedByOneAgent = 5.31 |

**Data assumptions:**

1. Hepatocytes account for 60% of liver cells, Kupffer Cells account for 15% of liver cells, SECs account for 20% of liver cells [[56](#_ENREF_56)], and mast cells account for 7.5% of liver cells [[57](#_ENREF_57)].
2. In general, we assume the change in rate is constant because we observed changes in data of interests in most of experimental studies following linear curves.
3. For some experimental data, we used multiple rates of synthesis or secretion. These multiple rates are explained in our experimental data table above.
4. Some experimental data is comprised of multiple linear segments, and therefore we calibrated rates for each linear segment to measure various rates for multiple responding time periods.
5. The release/secretion rates of various cytokines (TNF-α, HMGB-1 and IL-10) by inflammatory cells such as neutrophils, Kupffer Cells and monocyte-derived-macrophages are described as a function of time, by possibly incorporating the effect of decay/catabolism.
6. Experimental data are integrated into our agent-based model as inputs by ignoring different experimental conditions/settings such as different initial loads of bacteria injection, different bacteria strains, different animal models, etc. This limitation could be reduced by additional experiments done under the same experimental conditions/settings.
7. It was not possible to extrapolate the data for our agent-based model from one simple experimental model. The strategy we used was to focus on mouse Salmonella infection studies that were published in papers available in the NCBI. When necessary, we used data from broader systems such as Gram-negative infections (i.e. E. coli) or even Gram positive bacterial infections. Therefore, we are aware that some of these assumptions may not be correct.

**References:**

1. Nnalue N, Shnyra A, Hultenby K, Lindberg A. Salmonella choleraesuis and Salmonella typhimurium associated with liver cells after intravenous inoculation of rats are localized mainly in Kupffer cells and multiply intracellularly. Infection and Immunity. 1992;60(7):2758-2768.

2. Beuzón CR, Salcedo SP, Holden DW. Growth and killing of a Salmonella enterica serovar Typhimurium sifA mutant strain in the cytosol of different host cell lines. Microbiology. 2002;148(9):2705-2715.

3. Santos SAD, Andrade Júnior DRD, Andrade DRD. Tnf-a production and apoptosis in hepatocytes after listeria monocytogenes and salmonella typhimurium invasion. Revista do Instituto de Medicina Tropical de São Paulo. 2011;53(2):107-112.

4. azzaq Belaaouaj A, Kim KS, Shapiro SD. Degradation of outer membrane protein A in Escherichia coli killing by neutrophil elastase. Science. 2000;289(5482):1185-1187.

5.Weinrauch Y, Drujan D, Shapiro SD, Weiss J, Zychlinsky A. Neutrophil elastase targets virulence factors of enterobacteria. Nature. 2002;417(6884):91-94.

6. Conlan JW, North RJ. Early pathogenesis of infection in the liver with the facultative intracellular bacteria Listeria monocytogenes, Francisella tularensis, and Salmonella typhimurium involves lysis of infected hepatocytes by leukocytes. Infection and Immunity. 1992;60(12):5164-5171.

7. Richter-Dahlfors A, Buchan AM, Finlay BB. Murine salmonellosis studied by confocal microscopy: Salmonella typhimurium resides intracellularly inside macrophages and exerts a cytotoxic effect on phagocytes in vivo. The Journal of Experimental Medicine. 1997;186(4):569-580.

8. Russell DG, VanderVen BC, Glennie S, Mwandumba H, Heyderman RS. The macrophage marches on its phagosome: dynamic assays of phagosome function. Nature Reviews Immunology. 2009;9(8):594-600.

9. Nagl M, Kacani L, Müllauer B, Lemberger EM, Stoiber H, Sprinzl GM, et al. Phagocytosis and killing of bacteria by professional phagocytes and dendritic cells. Clinical and Diagnostic Laboratory Immunology. 2002;9(6):1165-1168.

10. Lajarin F, Rubio G, Galvez J, Garcia-Peñarrubia P. Adhesion, invasion and intracellular replication ofSalmonella typhimuriumin a murine hepatocyte cell line. Effect of cytokines and LPS on antibacterial activity of hepatocytes. Microbial Pathogenesis. 1996;21(5):319-329.

11. Brouckaert G, Kalai M, Krysko DV, Saelens X, Vercammen D, Ndlovu M, et al. Phagocytosis of necrotic cells by macrophages is phosphatidylserine dependent and does not induce inflammatory cytokine production. Molecular Biology of the Cell. 2004;15(3):1089-1100.

12. Lindgren SW, Stojiljkovic I, Heffron F. Macrophage killing is an essential virulence mechanism of Salmonella typhimurium. Proceedings of the National Academy of Sciences. 1996;93(9):4197-4201.

13. Ju ST, Cui H, Panka DJ, Ettinger R, Marshak-Rothstein A. Participation of target Fas protein in apoptosis pathway induced by CD4+ Th1 and CD8+ cytotoxic T cells. Proceedings of the National Academy of Sciences. 1994;91(10):4185-4189.

14. Abe T, Arai T, Ogawa A, Hiromatsu T, Masuda A, Matsuguchi T, et al. Kupffer cell–derived interleukin 10 is responsible for impaired bacterial clearance in bile duct–ligated mice. Hepatology. 2004;40(2):414-423.

15. Leist M, Gantner F, Jilg S, Wendel A. Activation of the 55 kDa TNF receptor is necessary and sufficient for TNF-induced liver failure, hepatocyte apoptosis, and nitrite release. The Journal of Immunology. 1995;154(3):1307-1316.

16. Tan JC, Indelicato SR, Narula SK, Zavodny PJ, Chou C. Characterization of interleukin-10 receptors on human and mouse cells. Journal of Biological Chemistry. 1993;268(28):21053-21059.

17. Gallin JI, Goldstein IM, Snyderman R. Inflammation: basic principles and clinical correlates. 2nd ed. New York: Raven Press; 1992.

18. Nemzek J, Bolgos G, Williams B, Remick D. Differences in normal values for murine white blood cell counts and other hematological parameters based on sampling site. Inflammation Research. 2001;50(10):523-527.

19. Grubb SC, Maddatu TP, Bult CJ, Bogue MA. Mouse phenome database. Nucleic Acids Research. 2009;37(Suppl 1):D720-D30.

20. Boxio R, Bossenmeyer-Pourié C, Steinckwich N, Dournon C, Nüße O. Mouse bone marrow contains large numbers of functionally competent neutrophils. Journal of Leukocyte Biology. 2004;75(4):604-611.

21. Coxon A, Tang T, Mayadas TN. Cytokine-activated endothelial cells delay neutrophil apoptosis in vitro and in vivo a role for granulocyte/macrophage colony-stimulating factor. The Journal of Experimental Medicine. 1999;190(7):923-934.

22. O'Dea KP, Wilson MR, Dokpesi JO, Wakabayashi K, Tatton L, van Rooijen N, et al. Mobilization and margination of bone marrow Gr-1high monocytes during subclinical endotoxemia predisposes the lungs toward acute injury. The Journal of Immunology. 2009;182(2):1155-1166.

23. Shi C, Velázquez P, Hohl TM, Leiner I, Dustin ML, Pamer EG. Monocyte trafficking to hepatic sites of bacterial infection is chemokine independent and directed by focal intercellular adhesion molecule-1 expression. The Journal of Immunology. 2010;184(11):6266-6274.

24. Yang J, Zhang L, Yu C, Yang XF, Wang H. Monocyte and macrophage differentiation: circulation inflammatory monocyte as biomarker for inflammatory diseases. Biomark Res. 2014;2(1):1. doi:  [10.1186/2050-7771-2-1](http://dx.doi.org/10.1186%2F2050-7771-2-1)

25. Takeishi T, Hirano K, Kobayashi T, Hasegawa G, Hatakeyama K, Naito M. The role of Kupffer cells in liver regeneration. Archives of Histology and Cytology. 1999;62(5):413-422.

26. Elmore S. Apoptosis: a review of programmed cell death. Toxicologic Pathology. 2007;35(4):495-516.

27. Patterson L, Higginbotham R. Mouse C-reactive protein and endotoxin-induced resistance. Journal of Bacteriology. 1965;90(6):1520-1524.

28. Bharadwaj D, Stein MP, Volzer M, Mold C, Du Clos TW. The major receptor for C-reactive protein on leukocytes is Fcγ receptor II. The Journal of Experimental Medicine. 1999;190(4):585-590.

29. Huang H, Nace GW, McDonald KA, Tai S, Klune JR, Rosborough BR, et al. Hepatocyte specific HMGB1 deletion worsens the injury in liver ischemia/reperfusion: a role for intracellular HMGB1 in cellular protection. Hepatology. 2014;59(5):1984-1997.

30. Cox G, Crossley J, Xing Z. Macrophage engulfment of apoptotic neutrophils contributes to the resolution of acute pulmonary inflammation in vivo. American Journal of Respiratory Cell and Molecular Biology. 1995;12(2):232-237.

31. Yang KK, Dorner BG, Merkel U, Ryffel B, Schütt C, Golenbock D, et al. Neutrophil influx in response to a peritoneal infection with Salmonella is delayed in lipopolysaccharide-binding protein or CD14-deficient mice. The Journal of Immunology. 2002;169(8):4475-4480.

32. Bian Z, Guo Y, Ha B, Zen K, Liu Y. Regulation of the inflammatory response: enhancing neutrophil infiltration under chronic inflammatory conditions. The Journal of Immunology. 2012;188(2):844-853.

33. Jaeschke H, Smith CW. Mechanisms of neutrophil-induced parenchymal cell injury. Journal of Leukocyte Biology. 1997;61(6):647-653.

34. Harbrecht BG, Billiar TR, Curran RD, Stadler J, Simmons RL. Hepatocyte injury by activated neutrophils in vitro is mediated by proteases. Annals of Surgery. 1993;218(2):120-128.

35. Jaeschke H. Mechanisms of liver injury. II. Mechanisms of neutrophil-induced liver cell injury during hepatic ischemia-reperfusion and other acute inflammatory conditions. American Journal of Physiology-Gastrointestinal and Liver Physiology. 2006;290(6):G1083-G1088.

36. Shi J, Gilbert GE, Kokubo Y, Ohashi T. Role of the liver in regulating numbers of circulating neutrophils. Blood. 2001;98(4):1226-1230.

37. Kasten KR, Muenzer JT, Caldwell CC. Neutrophils are significant producers of IL-10 during sepsis. Biochemical and Biophysical Research Communications. 2010;393(1):28-31.

38. Noel P, Nelson S, Bokulic R, Bagby G, Lippton H, Lipscomb G, et al. Pentoxifylline inhibits lipopolysaccharide-induced serum tumor necrosis factor and mortality. Life Sciences. 1990;47(12):1023-1029.

39. Retrieved from (2016, Apirl 5): https://www.shenandoah-bt.com/Mouse_Tumor_Necrosis_Factor_alpha.html.

40. Retrieved from (2016, Apirl 5): https://www.peprotech.com/en-US/Pages/Product/300-01A.

41. Retrieved from (2016, Apirl 5): https://www.rndsystems.com/products/recombinant-mouse-tnf-alpha-aa-80-235-protein_410-mt.

42. Retrieved from (2016, April 5) : http://www.prospecbio.com/TNF-alpha_Mouse_3_9/.

43. Sklar LA, McNeil V, Jesaitis A, Painter R, Cochrane C. A continuous, spectroscopic analysis of the kinetics of elastase secretion by neutrophils. The dependence of secretion upon receptor occupancy. Journal of Biological Chemistry. 1982;257(10):5471-5475.

44. Diesselhoff-den Dulk M, Crofton R, Van Furth R. Origin and kinetics of Kupffer cells during an acute inflammatory response. Immunology. 1979;37(1):7-14.

45. Calandra T, Bernhagen J, Mitchell RA, Bucala R. The macrophage is an important and previously unrecognized source of macrophage migration inhibitory factor. The Journal of Experimental Medicine. 1994;179(6):1895-1902.

46. Liu A, Fang H, Dirsch O, Jin H, Dahmen U. Oxidation of HMGB1 causes attenuation of its pro-inflammatory activity and occurs during liver ischemia and reperfusion. PloS One. 2012;7(4):e35379.

47. Sugihara A, Tsujimura T, Fujita Y, Nakata Y, Terada N. Evaluation of role of mast cells in the development of liver fibrosis using mast cell-deficient rats and mice. Journal of Hepatology. 1999;30(5):859-867.

48. Piliponsky AM, Chen CC, Grimbaldeston MA, Burns-Guydish SM, Hardy J, Kalesnikoff J, et al. Mast cell-derived TNF can exacerbate mortality during severe bacterial infections in C57BL/6-Kit W-sh/W-sh mice. The American Journal of Pathology. 2010;176(2):926-938.

49. Bissonnette E, Enciso J, Befus A. Inhibition of tumour necrosis factor‐alpha (TNF-α) release from mast cells by the anti‐inflammatory drugs, sodium cromoglycate and nedocromil sodium. Clinical & Experimental Immunology. 1995;102(1):78-84.

50. Seeley EJ, Sutherland RE, Kim SS, Wolters PJ. Systemic mast cell degranulation increases mortality during polymicrobial septic peritonitis in mice. Journal of Leukocyte Biology. 2011;90(3):591-597.

51. van Schaik SM, Abbas AK. Role of T cells in a murine model of Escherichia coli sepsis. European Journal of Immunology. 2007;37(11):3101-3110.

52. Schmidt-Weber C, Alexander S, Henault L, James L, Lichtman A. IL-4 enhances IL-10 gene expression in murine Th2 cells in the absence of TCR engagement. The Journal of Immunology. 1999;162(1):238-244.

53. Pepys MB, Hirschfield GM. C-reactive protein: a critical update. Journal of Clinical Investigation. 2003;111(12):1805-1812.

54. Souwer Y, Griekspoor A, Jorritsma T, de Wit J, Janssen H, Neefjes J, et al. B cell receptor-mediated internalization of salmonella: a novel pathway for autonomous B cell activation and antibody production. The Journal of Immunology. 2009;182(12):7473-7481.

55. Chung KH, Hwang HS, Lee KY. Kinetics of Salmonella typhimurium binding with antibody by immunoassays using a surface plasmon resonance biosensor. Journal of Industrial and Engineering Chemistry. 2010;16(1):115-118.

56. Malarkey DE, Johnson K, Ryan L, Boorman G, Maronpot RR. New insights into functional aspects of liver morphology. Toxicologic Pathology. 2005;33(1):27-34.

57. Farrell DJ, Hines JE, Walls AF, Kelly PJ, Bennett MK, Burt AD. Intrahepatic mast cells in chronic liver diseases. Hepatology. 1995;22(4):1175-1181.
